# Do authors of systematic reviews of epidemiological observational studies assess the methodologies of the included primary studies? An empirical examination of methodological tool use in the literature

**DOI:** 10.1186/s12874-024-02349-5

**Published:** 2024-10-08

**Authors:** Fabian Kemper, Clovis Mariano Faggion

**Affiliations:** Department of Periodontology and Operative Dentistry, Faculty of Dentistry, University Hospital Münster, Waldeyerstraße 30, 48149 Münster, Germany

**Keywords:** Observational study, Methods, Systematic review, Study characteristics

## Abstract

**Background:**

The procedures used to assess the methodological quality and risk of bias (RoB) of systematic reviews of observational dental studies have not been investigated. The purpose of this research was to examine the way that authors of systematic reviews of epidemiological observational studies published in dentistry conducted the methodological assessment of those primary studies. In the present article, we aimed to assess the characteristics and the level of reporting of tools used to assess the methodologies of these reviews.

**Methods:**

We searched Scopus and the Web of Science from their inceptions to June 2023 for systematic reviews with meta-analyses of observational studies published in dentistry. Document selection and data extraction were performed in duplicate and independently by two authors. In a random sample of 10% of the systematic reviews, there was an agreement of more than 80% between the reviewers; data selection and extraction were conducted in the remaining 90% of the sample by one author. Data on the article and systematic review characteristics were extracted and recorded for descriptive reporting.

**Results:**

The search in the two databases resulted in the inclusion of 3,214 potential documents. After the elimination of duplicates and the application of the eligibility criteria, a total of 399 systematic reviews were identified and included. A total of 368 systematic reviews reported a methodological tool, of which 102 used the Newcastle–Ottawa scale. Additionally, 76 systematic reviews stated the use of a modified methodological tool. Information about the approach of assessing the methodological quality or RoB of primary studies but reporting no tool or tool name occurred in 25 reviews.

**Conclusions:**

The majority of authors of systematic reviews of epidemiological observational studies published in dentistry reported the tools used to assess the methodological quality or RoB of the included primary studies. Modifying existing tools to meet the individual characteristics of various studies should be considered.

**Supplementary Information:**

The online version contains supplementary material available at 10.1186/s12874-024-02349-5.

## Introduction

Systematic reviews are important research that compile evidence from primary studies, such as those that address clinical interventions [1]. A systematic review can be accompanied by a meta-analysis that provides information to support clinical decisions [2]. However, a systematic review conducted with the highest methodological quality possible can still unfortunately include unreliable evidence. This is not necessarily the fault of systematic review authors but could be because of several methodological issues with the primary studies that are included. Therefore, it is mandatory that systematic reviews on interventions present a comprehensive assessment of the quality or risk of bias (RoB) of the primary studies included in these reviews. In this way, readers and decision-makers can adequately interpret the evidence coming from these reviews to make appropriate decisions.

Systematic reviews can also be applied to observational studies, for example, in the case of systematic reviews on the prevalence of diseases or conditions [3]. The same rationale for assessing the quality or risk of bias (RoB) of the primary studies should be followed with systematic reviews of observational studies. For example, a systematic review of the prevalence of a specific disease should contain information on the quality of the studies that provided information on this prevalence.

Several tools have been proposed to assess the methodological quality or RoB of primary studies included in systematic reviews [4]. For systematic reviews of randomised controlled trials (RCTs) of interventions, the RoB tool from Cochrane is likely the most widely used [5]. For observational studies, there is, for example, the Quality Assessment of Diagnostic Accuracy Studies (QUADAS) tool [6]. In dentistry, it is unclear whether a mapping of the types of methodological tools used to assess observational studies has been performed. Therefore, the present research had the following objectives: (a) to report the characteristics of tools used to assess the methodologies of epidemiological observational studies included in dental systematic reviews, and (b) to describe the level of reporting of these tools in the systematic reviews.

## Materials and methods

### Eligibility criteria

We included systematic reviews with meta-analyses of epidemiological observational studies published in dentistry. These meta-analyses included primary studies on prevalence, incidence, prognosis, diagnosis and aetiology. Systematic reviews of non-randomized intervention-related studies in clinical setting were excluded. Meta-analyses from interventional studies were excluded as well as any other type of study. Meta-analyses with outcomes on harm after any interventions were applied were also excluded. Meta-analyses involving areas other than the maxillofacial (for example, head and neck) were excluded.

### Definition of systematic reviews of epidemiological observational studies

In this meta-research, we defined systematic reviews of epidemiological observational studies as those including only studies where no interference or manipulation of the research subject had been conducted [7]. In other words, only systematic reviews of studies that assessed the exposure of a defined population (and not the intervention) were examined in this research.

### Definition of the term “methodologies” in this study

There are different concepts related to the methodological assessment of studies: reporting assessment measures how detailed the reporting of a study is; methodological quality assessment measures the quality of the study, but without assessing the impact of this quality on the results of the study; and RoB measures the risk of the study results being biased [8]. In the context of the present study, we used the term “methodology” as a proxy for methodological quality and RoB.

### Data search and selection

We searched the Scopus and Web of Science (WoS) databases on 16 June 2023 for systematic reviews published from each database’s inception to June 2023. The search strategy is reported in the supplementary file.

Articles reporting systematic reviews were selected by strictly following the eligibility criteria. First, titles/abstracts were assessed for inclusion, and if they did not meet the eligibility criteria, they were excluded, and the reasons for exclusion were recorded. The full text of the titles/abstracts initially included documents were then assessed for final inclusion. Again, if the full texts did not meet the eligibility criteria, they were excluded, and the reasons for exclusion were recorded. The selection was conducted independently and in duplicate by two assessors (FK, CMF) from the sample of publications. After agreement between the assessors was achieved (more than 80%), one assessor (FK) elected the remaining documents [9].

### Data extraction and analysis

The following data were directly extracted and recorded in an Excel spreadsheet: (a) title of the systematic review, (b) year of publication, (c) number of citations, (d) country, (e) number of authors, (f) H-index of the first author, (g) H-index of the last author, (h) dental journal, (i) journal impact factor (IF), (j) dental discipline, (k) type of systematic review, (l) number of included primary studies, (m) statement reporting conflict of interest, (n) sponsorship classification, (o) statement reporting registration in public database, (p) report of protocol number, (q) report of methodological tool, (r) name of the methodological tool, (s) number of reported tools, (t) report of validity of methodological tools, (u) report of modification of methodological tools, (v) type of modification of methodological tools, (w) number of authors who assessed the methodology and (x) duplication of assessment of methodology.

Data extraction was conducted independently and in duplicate by two assessors (FK, CMF) from the sample of documents. After agreement between the assessors was achieved (more than 80%) with 10% of the sample, one assessor (FK) extracted the remaining 90% of the sample [9].

As appropriate, descriptive results were presented as a mean with standard deviation (SD) or median with interquartile range (IQR).

## Results

### Selection process

**Fig. 1:**
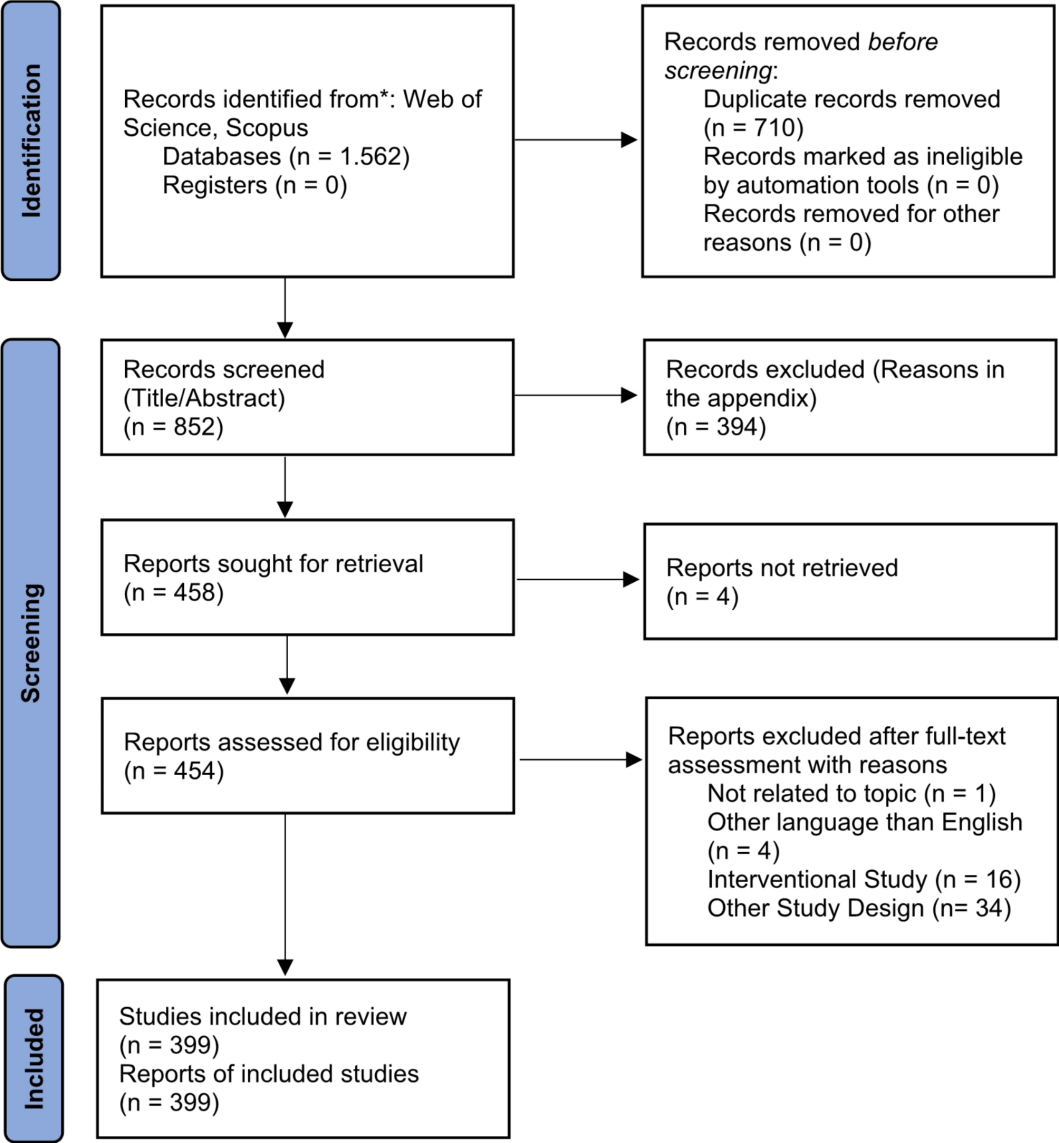
Flow of the selection process

The initial search retrieved 1,562 documents: 751 in Web of Science and 811 in Scopus. After removing duplicates from the different databases, the titles/abstracts of 852 unique documents were assessed. At its conclusion, 394 documents were excluded. After analysis of the full text of the remaining documents, 399 articles were included in this research. The search and selection processes are illustrated in detail in Fig. 1. A full list of documents not selected along with the specific exclusion reasons is reported in the supplementary file.

### Characteristics of the studies

**Fig. 2:**
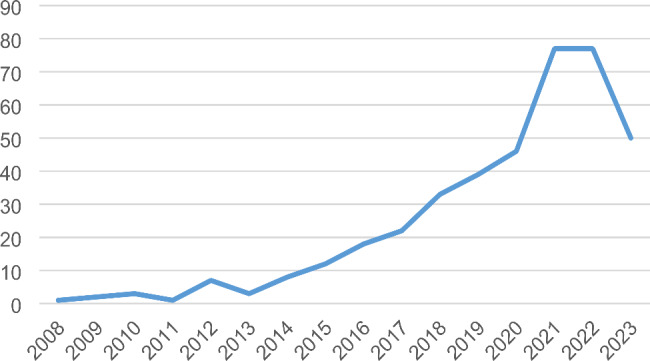
Publication of studies over the years

More than 50% (*N* = 204) of the systematic reviews included in this assessment were published between 2021 and 2023 (Fig. 2). Authors from Asia and South America were responsible for the greatest number of documents in the sample, with 132 (33.08%) and 122 (30.58%), respectively. The most prevalent country with which a corresponding author was affiliated was Brazil (*N* = 119, 29.82%).

The systematic reviews were published in many different journals, but 21.5% (*N* = 86) of them were published in *Clinical Oral Investigations* (*N* = 25), *Oral Diseases* (*N* = 24), *Oral Oncology* (*N* = 20), and the *Journal of Oral Pathology and Medicine* (*N* = 17). The mean journal IF was 3.11 (SD = 1.65). Systematic reviews of prevalence studies were the most frequent in the sample, with *N* = 169 (42.36%).

A statement of a potential conflict of interest for authors of the systematic reviews was reported in 336 (84.21%) reviews, and sponsorship information was reported in 243 (60.09%) reviews. The authors of 181 (45.36%) systematic reviews reported that the methodological assessment of primary studies was conducted in duplicate, and the median number of reported authors assessing the methodology was 2.11 (SD = 0.64). The characteristics of the systematic reviews are reported in Table 1.

**Table 1:** Characteristics of the studies

**Study Characteristics**	**Mean (standard deviation)**
Number of citations	50.33 (94.08)
Number of authors	5.68 (2.34)
H-index of first author	9.13 (10.88)
H-index of last author	28.49 (17.28)
Journal Impact Factor	3.11 (1.65)
Number of included primary studies	27.23 (26.66)
**Study Characteristics**	**Frequency (%)**
**Year of publication**	
2008	1 (0.25)
2009	2 (0.50)
2010	3 (0.75)
2011	1 (0.25)
2012	7 (1.75)
2013	3 (0.75)
2014	8 (2.01)
2015	12 (3.01))
2016	18 (4.51)
2017	22 (5.51)
2018	33 (8.27)
2019	39 (9.77)
2020	46 (11.53)
2021	77 (19.30)
2022	77 (19.30)
2023	50 (12.53)
**Continent of the corresponding author**	
South America	122 (30.58)
Asia	132 (33.08)
Europe	108 (27.07)
Africa	4 (1.00)
North America	19 (4.76)
Oceania	14 (3.51)
**Dental journal**	
Clinical Oral Investigations	25 (6.27)
Oral Diseases	24 (6.01)
Oral Oncology	20 (5.01)
Journal of Oral Pathology and Medicine	17 (4.26)
Journal of Dentistry	15 (3.76)
Archives of Oral Biology	13 (3.26)
Journal of Oral Rehabilitation	13 (3.26)
Dental Traumatology	12 (3.01)
Dentomaxillofacial Radiology	11 (2.76)
BMC Oral Health	11 (2.76)
Journal of Clinical Periodontology	11 (2.76)
Journal of Endodontics	10 (2.51)
International Journal of Paediatric Dentistry	10 (2.51)
Other	207 (51.88)
**Dental discipline**	
Oral and Maxillofacial Pathology	135 (33.83
Oral and Maxillofacial Radiology	47 (11.78)
Periodontics	42 (10.53)
Endodontics	40 (10.03)
Prosthodonticsy	39 (9.77)
Pediatric Dentistry	28 (7,02)
Dental Public Health	22 (5.51)
Orthodontics	17 (4.26)
Oral and Maxillofacial Surgery	15 (3.76)
Other	13 (3.26)
Oral Medicine	1 (0.25)
**Type of systematic review**	
Prevalence	169 (42.36)
Risk Factor	86 (21.55)
Diagnostic Test Accuracy	83 (20.80)
Etiology	33 (8.27)
Prognosis	17 (4.26)
Incidence	11 (2.76)
**Statement reporting conflict of interest**	
Statement reported	336 (84.21)
Statement not reported	63 (15.79)
**Sponsorship classification**	
No sponsor	106 (26.57)
Industry sponsorship	3 (0.75)
Non-industry sponsorship	133 (33.34)
Industry and non-industry sponsorship	1 (0.25)
Unclear or not reported	156 (39.10)
**Registration of Systematic Review reported**	
Reported	233 (58.40)
Not reported	166 (41.60)
**Report of Protocol Number**	
Reported	224 (56.14)
Not reported	175 (43.86)
**Report of methodological tool**	
Reported	368 (92.23)
Not Reported	31 (7.77)
**Number of reported tools**	
0	31 (7.77)
1	349 (87.47)
2	18 (4.51)
3	1 (0.25)

Report of methodological tool: Number of studies where authors reported the use of a methodological tool for assessing the quality of primary studies

Number of reported tools: Number of methodological tools reported in studies for assessing the quality of primary studies

### Methodological tools

In 368 (92.23%) systematic reviews the authors reported the use of a methodological tool. More than 10 different methodological tools were used by the authors of the systematic reviews to analyse the quality of the included primary studies. A combination of two or more tools was reported in 19 (4.76%) of the reviews.

The most commonly used methodological tools were the different versions of the Newcastle–Ottawa scale (*N* = 102, 25.56%) and the Joanna Briggs Institute Critical Appraisal Checklist (*N* = 87, 21.8%). In 76 (19.50%) systematic reviews, the authors reported the use of a modified tool, in which the scoring, the number of items/questions/domains or even both were adjusted to the individual aims of their systematic review. In only 9 (2.26%) systematic reviews, the authors reported the validity of the methodological tools used. A total of 25 (6.27%) reviews reported an approach to quality assessment but did not report a tool or the name of the tool that was used. No information about assessing the methodological quality of primary studies was reported in 19 (4.76%) of the systematic reviews. Detailed information on the methodological tools are reported in Table 2.

**Table 2:** Methodological tools

**Methodological tool**	**Frequency (%)**
Newcastle Ottawa scale	102 (25.56)
Joanna Briggs Institute critical appraisal checklist	87 (21.80)
Quality assessment of diagnostic accuracy studies tool (QUADAS-2)	69 (17.29)
Strengthening the reporting of observational studies in epidemiology (STROBE)	11 (2.76)
Quality assessment checklist for prevalence studies (Hoy et al.)	11 (2.76)
Fowkes and Fulton critical appraisal checklist	9 (2.25)
Quality in prognosis studies (QUIPS)	8 (2.01)
Meta-Analysis of Statistics Assessment and Review Instruments (MAStARI)	8 (2.01)
Cochrane risk-of-bias tool	6 (1.50)
Quality assessment based on modified criteria of Loney et al.	4 (1.00)
Information about approach but no tool or tool name reported	25 (6.27)
No information provided	19 (4.76)
Others	40 (10.03)
**Second methodological tool**	
Strengthening the reporting of observational studies in epidemiology (STROBE)	2 (0.5)
Cochrane risk-of-bias-tool	2 (0.5)
Quality in prognosis studies (QUIPS)	2 (0.5)
Only one methodological tool	380 (95.24)
**Report of validity of methodological tools**	
Reported	9 (2.26)
Not reported	359 (89.97)
Not applicable	31 (7.77)
**Report of modification of methodological tools**	
Reported	76 (19.05)
Not reported	292 (73.18)
Not applicable	31 (7.77)
**Type of modification of methodological tools**	
Number of items/questions/domains	15 (3.76)
Way of scoring/rating	9 (2.26)
Both (number and way)	5 (1.25)
Unclear	45 (11.27)
Not applicable	325 (81.45)
**Number of authors who assessed methodology**	
1	5 (1.25)
2	224 (56.14)
3	13 (3.26)
4	3 (0.75)
5	1 (0.25)
6	1 (0.25)
9	1 (0.25)
Unclear	120 (30.07)
Not applicable	31 (7.77)
**Duplication of assessment of methodology**	
Duplicated	181 (45.36)
Partially duplicated	3 (0.75)
Unclear	184 (46.12)
Not applicable	31 (7.77)

Second methodological tool: Name of the second methodological tool when authors used not only one tool to assess the quality of primary studies

Only one methodological tool: Number of studies where only one methodological tool was reported for assessing the quality of primary studies

## Discussion

### Main findings

The aim of this research was to examine if and how authors of systematic reviews of epidemiological observational studies published in the field of dentistry assessed the methodologies of the primary studies included in their reviews. Within the extensive sample of reviews examined, the majority of authors (92.23%) reported the use of a methodological tool. The most frequently reported tool used for quality assessment was the Newcastle–Ottawa scale (*N* = 102, 25.56%). Systematic reviews published from 2020 onwards showed better reporting of the methodologies assessment of their primary studies than those published between 2008 and 2019.

### Interpretation of results

Our findings showed that there were still authors who did not report the assessment of the methodologies of primary studies at all (7.77%). Although some of the authors (6.27%) of the systematic reviews stated that they examined the methodologies of the primary studies, they either did not provide the name of the methodological tool used or did not use one. Possible explanations for this could be that there was no single obvious candidate tool for assessing the quality of observational epidemiological studies [8], and authors were unable to adapt the criteria for assessment tools commonly employed for their studies [10]. In 19 systematic reviews, no information was provided on the methodology assessment of the primary studies.

It is noteworthy that a great number of different tools were used to examine the methodological quality of the epidemiological observational studies: We identified more than 35 different tools, checklists, guidelines and scales, but the largest proportion of systematic reviews was spread across 10 different tools. Another systematic review of observational studies in epidemiology in general identified 86 tools that were used for assessing quality and susceptibility to bias [11]. In our study, by far the most used tools were the Newcastle–Ottawa scale, the Joanna Briggs Institute Critical Appraisal Checklist and the Quality Assessment of Diagnostic Accuracy tool (QUADAS-2), which together accounted for 64.65%.

The Newcastle–Ottawa scale is a collaboration between the University of Newcastle (Australia) and the University of Ottawa (Canada). It contains eight items, categorised into three dimensions: selection, comparability and outcome (for cohort studies) or exposure (for case-control studies). It is based on a star system in which a study is rated from three perspectives [12]. Through this, it is possible to semi-quantitatively evaluate the study quality, where the highest-quality studies are awarded with a maximum of one star for each item, except for the item related to comparability, which allows two stars. The range of the Newcastle–Ottawa scale is zero to nine stars [13].

The Critical Appraisal Checklist developed by the Joanna Briggs Institute is a checklist with (depending on the study type) nine to eleven different questions, all of which must be answered with ‘yes’, ‘no’, ‘unclear’ or ‘not applicable’ [14]. This helps determine in which way a study has addressed potential bias in its design, conduct and analysis [15].

The Quality Assessment of Diagnostic Accuracy tool (QUADAS-2) consists of four key domains: patient selection, index test, reference standard and flow and timing. The tool is completed in four phases: (1) state the review question, (2) develop review-specific guidance, (3) review the published flow diagram for the primary study or construct a flow diagram if none is reported and (4) judgement of bias and applicability. For every single domain, an assessment is conducted in terms of RoB and, for the first three domains, is also assessed in terms of concerns regarding applicability. Signalling questions are also part of the tool to assist in reaching a judgement on the RoB [6].

In addition, we found that the number of reviews of epidemiological observational studies in the field of dentistry has increased significantly in recent years. While the number of published systematic reviews in 2010, for example, was only three, by 2022, it was already 77. It is striking that the authors of the older systematic reviews did not specify a tool more often; of 31 reviews that did not report a methodological tool, 18 were published between 2008 and 2019, which seems even more significant when you consider that only 37.34% of the total number of reviews analysed were published during that period. One possible reason for the improvement in the quality of the investigation of primary studies is the recent publication of guidance on conducting systematic reviews and meta-analyses of observational studies which emphasizes the assessment of the methodology of studies as a crucial element of any systematic review [16].

A methodology assessment should be conducted by two independent reviewers to ensure that the results of the systematic review are accurate [17]. A total of 224 (56.14%) systematic reviews reported assessment by two reviewers, and 181 (45.36%) stated that the assessment was conducted in duplicate. The number of systematic reviews in which it was unclear how many reviewers checked the methodology of primary studies was 120 (30.07%). The number of reviews in which it was unclear if the assessment was carried out in duplicate was even higher: 184 (46.12%) reviews did not provide information about assessing in duplicate. The reviewers may have performed the highest-quality systematic reviews, but the primary studies included in their reviews might still contain a high risk of bias [17]; this makes it critically important to provide the best possible report of quality assessment.

Epidemiological observational studies are studies that answer a research question simply with what the author or researcher observes, which means that no interventions, manipulative treatment or other interference is part of the research [18]. One of the main reasons for conducting an observational study is that the authors have ethical or practical concerns about a common experiment. For example, when assessing the effect of antibiotics to prevent endocarditis in patients with a high risk of developing endocarditis [19] and needing periodontal treatment, it would be unethical to plan a more robust study in the form of RCT.

The biggest difference between an observational study and an experimental study is that the authors of observational studies never try to affect outcomes, while experimental studies by definition are designed to produce results through certain treatments and interventions [20]. Observational studies have a higher risk of observer bias and confounding variables [7], which makes it even more important for the authors of systematic reviews of observational studies to accurately check the methodological quality of the primary studies included in their reviews. We identified different types of epidemiological observational studies and subdivided them into studies on prevalence, risk factor, diagnostic test accuracy, aetiology, prognosis and incidence. Sometimes, the transitions between studies are fluid, and it is thus challenging in a systematic review to differentiate one type of study from another. There are also systematic reviews that examine, for example, the prevalence and incidence of a particular topic. For example, Petti et al. [21] assessed the prevalence rates of permanent dentition and primary dentition and, in 12-year-olds, the incidence rate of any tooth for any age. The main type of study examined by systematic reviews is not always apparent at first glance. For example, Rahman et al. [22] investigated not only the incidence of the co-infection of the Epstein–Barr virus and human papillomavirus in oropharyngeal squamous cell carcinoma but also the prevalence and molecular mechanisms of the aetiology.

Another finding in our research was that some systematic reviews (*n* = 18) included a second methodological tool to assess the methodological quality of the included primary studies. This finding suggests that the systematic review authors might have included primary studies with different characteristics or designs. For each systematic review, we focused on the main question and used it for classification.

Our study also included studies where authors applied tools not originally designed to assess methodological quality or RoB. This was the case with the use of the STROBE checklist for assessing the reporting of epidemiological observational studies. Authors used this checklist to assess the methodological quality of the primary studies included in their systematic reviews [23–30]. These findings corroborate previous research on the misuse of methodological tools [8, 31].

### Limitations and strengths

To the best of our knowledge, this research appears to be the first in dentistry to assess how the authors of systematic reviews of epidemiological observational studies assessed the methodological quality of primary studies included in the reviews. Not only does it provide useful information about methodological quality assessment and reporting standards, but it also provides guidance for authors planning studies in the future. The sample was robust, although one can consider that the data reported here may be only representative of the field of dentistry.

It should be noted that it appeared to be challenging to examine whether a systematic review really included only observational studies. We predefined the criteria for reviews included in our study and excluded those that did not meet the inclusion criteria entirely. We excluded studies that were not published in English, which led to a reduction in the number of included studies. However, the number of studies that were excluded due to being published in another language from the database results was so small that this did not have a large effect on the results. By using well-known databases, such as Scopus and Web of Science, it is very likely that our sample was the best representation of the currently available evidence on this topic.

## Conclusion

Of the 399 systematic reviews with meta-analyses of epidemiological observational studies in dentistry, the majority reported a methodological tool or at least an approach, for assessing the methodologies of the included primary studies. There was an encouraging trend that the number of authors who did not report any information about quality assessment was decreasing over the last few years. However, it is important for authors to ensure that the chosen tool is appropriate for the individual aim of their systematic review and modifications to existing tools should be considered.

## Electronic supplementary material

Below is the link to the electronic supplementary material.

Supplementary Material 1

Supplementary Material 2

## Data Availability

Search strategies, the list of included articles, and the lists of excluded articles with reasons of exclusion are reported in the supplementary file
