# Supplementary material for: Do authors of systematic reviews of epidemiological observational studies assess the methodologies of the included primary studies? An empirical examination of methodological tool use in the literature
Source: BMC Med Res Methodol. 2024 Oct 8;24:233. doi: 10.1186/s12874-024-02349-5 (PMC11459945; doi:10.1186/s12874-024-02349-5)
Supplement: Supplementary file 1 — Supplementary Material 1 [file 12874_2024_2349_MOESM1_ESM.docx]

**Supplementary files**

Search strategy conducted in Scopus

4 ( TITLE ( meta-analysis OR metanalysis OR metaanalysis OR "meta analysis" ) ) AND ( ABS ( prevalence OR incidence OR aetiology OR etiology OR diagnostic OR prognosis ) ) AND ( LIMIT-TO ( SUBJAREA , "dent" ) )

814 results

3 ( TITLE ( meta-analysis OR metanalysis OR metaanalysis OR "meta analysis" ) ) AND ( ABS ( prevalence OR incidence OR aetiology OR etiology OR diagnostic OR prognosis ) )

Show more

49,385 results

2 TITLE ( meta-analysis OR metanalysis OR metaanalysis OR "meta analysis" )

198,166 results

1 ABS ( prevalence OR incidence OR aetiology OR etiology OR diagnostic OR prognosis )

3,831,131 results

Scopus

EXPORT DATE: 16 June 2023

Search strategy conducted in Web of Science

4 #2 AND #1 and Dentistry Oral Surgery Medicine (Web of Science Categories)

752

3 #2 AND #1

45,617

2 TI=(meta-analysis OR metanalysis OR metaanalysis OR "meta analysis" )

214,411

1 prevalence OR incidence OR aetiology OR etiology OR diagnostic OR prognosis (Abstract)

2,578,462

*List of included studies*

1. Shi F-P, Huang Y-Y, Dai Q-Q, Chen Y-L, Jiang H-Y, Liang S-Y. Maternal Common Cold or Fever During Pregnancy and the Risk of Orofacial Clefts in the Offspring: A Systematic Review and Meta-analysis. Cleft Palate-Craniofac J. 2023;60:446–53.

2. Minervini G, Franco R, Marrapodi MM, Fiorillo L, Cervino G, Cicciù M. Prevalence of temporomandibular disorders (TMD) in pregnancy: A systematic review with meta-analysis. J Oral Rehabil. 2023. https://doi.org/10.1111/joor.13458.

3. de Oliveira-Neto OB, Barbosa FT, de Lima FJC, de Sousa-Rodrigues CF. Prevalence of canalis sinuosus and accessory canals of canalis sinuosus on cone beam computed tomography: a systematic review and meta-analysis. Int J Oral Maxillofac Surg. 2023;52:118–31.

4. Mohammadi H, Roochi MM, Heidar H, Garajei A, Dallband M, Sadeghi M, et al. A meta-analysis to evaluate the prevalence of maxillofacial trauma caused by various etiologies among children and adolescents. Dent Traumatol. 2023. https://doi.org/10.1111/edt.12845.

5. Cui Y, Chen D, Lin H, Tao Y. The association between low birth weight and/or preterm birth and dental caries -A systematic review and meta-analysis. Int J Dent Hyg. 2023. https://doi.org/10.1111/idh.12651.

6. Abdolalian F, Bayani M, Afzali S, Nakhostin A, Almasi-Hashiani A. Periostin level in gingival crevicular fluid in periodontal disease: a systematic review and meta-analysis. BMC Oral Health. 2023;23.

7. Knorst JK, Tomazoni F, Sfreddo CS, Vettore M, Hesse D, Ardenghi TM. Social capital and oral health in children and adolescents: A systematic review and meta-analysis. Community Dent Oral Epidemiol. 2022;50:461–8.

8. Talal AlSharif M, Abdullah Alamoudi R, Jafar Sabbagh H. Maternal stress as a risk factor for non-syndromic orofacial clefts: Systematic review and meta-analysis. Saudi Dent J. 2023;35:207–19.

9. Martins M, Mascarenhas P, Evangelista JG, Barahona I, Tavares V. The Incidence of Dental Caries in Children with Down Syndrome: A Systematic Review and Meta-Analysis. Dentistry J. 2022;10.

10. De Porras-Carrique T, Ramos-García P, Aguilar-Diosdado M, Warnakulasuriya S, González-Moles MÁ. Autoimmune disorders in oral lichen planus: A systematic review and meta-analysis. Oral Dis. 2023;29:1382–94.

11. González-Moles M, de Porras-Carrique T, Ramos-García P. Association of oral lichen planus with hepatic disorders and hepatocellular carcinoma: systematic review and meta-analysis. Med Oral Patol Oral Cir Bucal. 2023;28:e229–37.

12. Bento VAA, Gomes JML, Lemos CAA, Limirio JPJO, Rosa CDDRD, Pellizzer EP. Prevalence of proximal contact loss between implant-supported prostheses and adjacent natural teeth: A systematic review and meta-analysis. J Prosthet Dent. 2023;129:404–12.

13. Ma Y, Mu D, Li X. Risk factors for root resorption of second molars with impacted third molars: a meta-analysis of CBCT studies. Acta Odontol Scand. 2023;81:18–28.

14. Batistella EÂ, Gondak R, Rivero ERC, Warnakulasuriya S, Guerra E, Porporatti AL, et al. Comparison of tobacco and alcohol consumption in young and older patients with oral squamous cell carcinoma: a systematic review and meta-analysis. Clin Oral Invest. 2022;26:6855–69.

15. Abesi F, Yousefi MJ, Zamani M. Prevalence and anatomical characteristics of maxillary sinus septa: A systematic review and meta-analysis of cone-beam computed tomography studies. J Oral Maxillofacial Surg Med Pathol. 2023. https://doi.org/10.1016/j.ajoms.2023.03.015.

16. González-Mancilla S, Montero-Miralles P, Saúco-Márquez JJ, Areal-Quecuty V, Cabanillas-Balsera D, Segura-Egea JJ. Prevalence of Dens Invaginatus assessed by CBCT: Systematic Review and Meta-Analysis. J Clini Exp Dent. 2022;14:959–66.

17. da Costa TMP, Nascimento MDCC, Peralta-Mamani M, Rubira-Bullen IRF, Junqueira JLC, Soares MQS. Prevalence of orofacial alterations in patients with sickle-cell disease: Systematic review and meta-analysis. Oral Surg Oral Med Oral Pathol Oral Radiol. 2023;135:642–60.

18. Borg-Bartolo R, Roccuzzo A, Molinero-Mourelle P, Schimmel M, Gambetta-Tessini K, Chaurasia A, et al. Global prevalence of edentulism and dental caries in middle-aged and elderly persons: A systematic review and meta-analysis. J Dent. 2022;127.

19. Souza MRF, Gonçalves MWA, de Souza GM, Fernandes IA, Falci SGM, Galvão EL. Oral and maxillofacial trauma in women assaulted by men: Systematic review and meta-analysis. J Stomatol Oral Max Surg. 2023;124.

20. Aung NM, Myint KK. Bifid Mandibular Canal: A Proportional Meta-Analysis of Computed Tomography Studies. Int J Dent. 2023;2023.

21. Minervini G, Franco R, Marrapodi MM, Fiorillo L, Cervino G, Cicciù M. Economic inequalities and temporomandibular disorders: A systematic review with meta-analysis. J Oral Rehabil. 2023. https://doi.org/10.1111/joor.13491.

22. Mehta J, Eaton C, AlAmri M, Lin G-H, Nibali L. The association between Aggregatibacter actinomycetemcomitans JP2 clone and periodontitis: A systematic review and meta-analysis. J Periodontal Res. 2023;58:465–82.

23. de Moura RC, Santos PS, Matias PMDS, Vitali FC, Hilgert LA, Cardoso M, et al. Knowledge, attitudes, and practice of dentists on Minimal Intervention Dentistry: A systematic review and meta-analysis. J Dent. 2023;132.

24. Rahman R, Shaikh MH, Gopinath D, Idris A, Johnson NW. Human papillomavirus and Epstein-Barr virus co-infection in oral and oropharyngeal squamous cell carcinomas: A systematic review and meta-analysis. Mol Oral Microbiol. 2023. https://doi.org/10.1111/omi.12412.

25. Skeie MS, Sen A, Dahllöf G, Fagerhaug TN, Høvik H, Klock KS. Dental caries at enamel and dentine level among European adolescents – a systematic review and meta-analysis. BMC Oral Health. 2022;22.

26. Evangelista K, de Freitas Silva BS, Yamamoto-Silva FP, Valladares-Neto J, Silva MAG, Cevidanes LHS, et al. Accuracy of artificial intelligence for tooth extraction decision-making in orthodontics: a systematic review and meta-analysis. Clin Oral Invest. 2022;26:6893–905.

27. Guan J-Y, Luo Y-H, Lin Y-Y, Wu Z-Y, Ye J-Y, Xie S-M, et al. Malignant transformation rate of oral leukoplakia in the past 20 years: A systematic review and meta-analysis. J Oral Pathol Med. 2023. https://doi.org/10.1111/jop.13440.

28. Heller MA, Nyirjesy SC, Balsiger R, Talbot N, VanKoevering KK, Haring CT, et al. Modifiable risk factors for oral cavity cancer in non-smokers: A systematic review and meta-analysis. Oral Oncol. 2023;137.

29. Sadr S, Mohammad-Rahimi H, Motamedian SR, Zahedrozegar S, Motie P, Vinayahalingam S, et al. Deep Learning for Detection of Periapical Radiolucent Lesions: A Systematic Review and Meta-analysis of Diagnostic Test Accuracy. J Endod. 2023;49:248-261.e3.

30. Xu F, Tang J. Is There An Association Between Periodontitis And Non-Alcoholic Fatty Liver Disease? A Systematic Review and Meta-Analysis. Community Dent Health. 2023;40:47–52.

31. Al-Maswary AA, Almadhoon HW, Elkhateb A, Hamdallah A, Halboub E. The Global Prevalence of Middle Mesial Canal in Mandibular First and Second Molars Assessed by Cone Beam Computed Tomography: A Systematic Review and Meta-Analysis. J Endod. 2023. https://doi.org/10.1016/j.joen.2023.04.008.

32. Sampaio C, Pessan JP, Nunes GP, Magno MB, Maia LC, Exterkate R, et al. Are the counts of Streptococcus mutans and Staphylococcus aureus changed in complete denture wearers carrying denture stomatitis? A systematic review with meta-analyses. J Prosthet Dent. 2023. https://doi.org/10.1016/j.prosdent.2023.03.015.

33. Alves dos Santos GN, Sousa-Neto MD, Assis HC, Lopes-Olhê FC, Faria-e-Silva AL, Oliveira ML, et al. Prevalence and morphological analysis of dens invaginatus in anterior teeth using cone beam computed tomography: A systematic review and meta-analysis. Arch Oral Biol. 2023;151.

34. Londono J, Ghasemi S, Lawand G, Dashti M. Evaluation of the golden proportion in the natural dentition: A systematic review and meta-analysis. J Prosthet Dent. 2023;129:696–702.

35. Chaweeborisuit P, Yurasakpong L, Kruepunga N, Tubbs RS, Chaiyamoon A, Suwannakhan A. The prevalence of Stafne bone cavity: A meta-analysis of 355,890 individuals. J Dent Sci. 2023;18:594–603.

36. Musa AAR, Sethi S, Poirier BF, Oliver KJ, Jensen ED. Non-traumatic emergency department dental visits among patients 0–25 years of age: A systematic review and meta-analysis. Int J Paediatr Dent. 2023. https://doi.org/10.1111/ipd.13069.

37. Reis INRD, do Amaral GCLS, Hassan MA, Villar CC, Romito GA, Spin-Neto R, et al. The influence of smoking on the incidence of peri-implantitis: A systematic review and meta-analysis. Clin Oral Implants Res. 2023;34:543–54.

38. Abreu MHD, da Silva APL, Cavalcanti RVA, Cecilio Hallak Regalo S, Siéssere S, Gonçalves FM, et al. Prevalence of chewing difficulty in older people in long-term care: A systematic review and meta-analysis. Gerodontology. 2023;40:10–25.

39. Vitali FC, Santos PS, Massignan C, Maia LC, Cardoso M, Teixeira CDS. Global Prevalence of Maxillary Sinusitis of Odontogenic Origin and Associated Factors: A Systematic Review and Meta-Analysis. J Endod. 2023;49:369-381.e11.

40. Juárez-López MLA, Salazar-Treto LV, Hernández-Monjaraz B, Molina-Frechero N. Etiological Factors of Molar Incisor Hypomineralization: A Systematic Review and Meta-Analysis. Dentistry J. 2023;11.

41. Thapar PR, Nadgere JB, Iyer J, Salvi NA. Diagnostic accuracy of ultrasonography compared with magnetic resonance imaging in diagnosing disc displacement of the temporomandibular joint: A systematic review and meta-analysis. J Prosthet Dent. 2023. https://doi.org/10.1016/j.prosdent.2023.03.012.

42. Kimmie-Dhansay F, Bhayat A. Prevalence of dental caries in the permanent dentition amongst 12-year-olds in Africa: a systematic review and meta-analysis. BMC Oral Health. 2022;22.

43. Pedroso CM, Normando AGC, Siracusa CS, Lauby-Secretan B, Nethan ST, Tomasi RA, et al. Pan-American prevalence of smokeless tobacco use and association with oral potentially malignant disorders and head and neck cancer: a systematic review and meta-analysis. Oral Surg Oral Med Oral Pathol Oral Radiol. 2023. https://doi.org/10.1016/j.oooo.2023.02.019.

44. Bhadauria US, Purohit B, Agarwal D, Priya H. Dental caries status in individuals with hearing difficulties: A systematic review and meta-analysis. Spec Care Dent. 2023;43:9–16.

45. Minervini G, Franco R, Marrapodi MM, Ronsivalle V, Shapira I, Cicciù M. Prevalence of temporomandibular disorders in subjects affected by Parkinson disease: A systematic review and metanalysis. J Oral Rehabil. 2023. https://doi.org/10.1111/joor.13496.

46. Liu W, Zhang Q, Bao Z, Shen X. A potential reciprocal emergence of regulatory T cells in oral lichen planus and pemphigus vulgaris: A meta-analysis. J Dent Sci. 2023;18:437–42.

47. Azzola LG, Fankhauser N, Srinivasan M. Influence of the vegan, vegetarian and omnivore diet on the oral health status in adults: a systematic review and meta-analysis. Evid-based Dent. 2023;24:43–4.

48. Baena-de la Iglesia T, Yañez-Vico RM, Iglesias-Linares A. DIAGNOSTIC PERFORMANCE OF CONE-BEAM COMPUTED TOMOGRAPHY TO DIAGNOSE IN VIVO/IN VITRO ROOT RESORPTION: A SYSTEMATIC REVIEW AND META-ANALYSIS. J Evid-Based Dent Pract. 2023;23.

49. Nascimento EB, Rodrigues R, Manso MC. Prevalence of dental floss use in deciduous dentition: A systematic review and meta-analysis. Int J Dent Hyg. 2023;21:116–27.

50. Bayani M, Raisolvaezin K, Almasi-Hashiani A, Mirhoseini SH. Bacterial biofilm prevalence in dental unit waterlines: a systematic review and meta-analysis. BMC Oral Health. 2023;23:158.

51. Gonde N, Rathod S, Kolte A, Lathiya V, Ughade S. Association between tooth loss and risk of occurrence of oral cancer - A systematic review and meta-analysis. Dent Res J. 2023;20:4.

52. Minervini G, Franco R, Marrapodi MM, Fiorillo L, Cervino G, Cicciù M. Prevalence of temporomandibular disorders in children and adolescents evaluated with Diagnostic Criteria for Temporomandibular Disorders: A systematic review with meta-analysis. J Oral Rehabil. 2023;50:522–30.

53. Diaz P, Gonzalo E, Villagra LJG, Miegimolle B, Suarez MJ. What is the prevalence of peri-implantitis? A systematic review and meta-analysis. BMC Oral Health. 2022;22.

54. Normando AGC, dos Santos ES, Sá JDO, Busso-Lopes AF, De Rossi T, Patroni FMDSá., et al. A meta-analysis reveals the protein profile associated with malignant transformation of oral leukoplakia. Front Oral Health. 2023;4.

55. da Motta TP, Owens J, Abreu LG, Debossan SAT, Vargas-Ferreira F, Vettore MV. Malocclusion characteristics amongst individuals with autism spectrum disorder: a systematic review and meta-analysis. BMC Oral Health. 2022;22.

56. Lima TCDS, Coste SC, Fernandes MIAP, Barbato-Ferreira DA, Colosimo EA, Del Fabbro M, et al. Prevalence of traumatic dental injuries in emergency dental services: A systematic review and meta-analysis. Community Dent Oral Epidemiol. 2023;51:247–55.

57. Machado RA, Popoff DAV, Martelli-Júnior H. Relationship between non-syndromic oral clefts and cancer: A systematic review and meta-analysis. Oral Dis. 2022;28:1369–86.

58. Henriques I, Caramês J, Francisco H, Caramês G, Hernández-Alfaro F, Marques D. Prevalence of maxillary sinus septa: systematic review and meta-analysis. Int J Oral Maxillofac Surg. 2022;51:823–31.

59. da Silva YS, Sohal KS, Stoelinga PJW, Grillo R. A meta-analysis on the presentation of Unicystic Ameloblastoma in the jaws and the consequences for their treatment. J Stomatol Oral Max Surg. 2022;123:e433–8.

60. De Porras-Carrique T, González-Moles MÁ, Warnakulasuriya S, Ramos-García P. Depression, anxiety, and stress in oral lichen planus: a systematic review and meta-analysis. Clin Oral Invest. 2022;26:1391–408.

61. Rotbeh A, Kazeminia M, Kalantari M, Rajati F. Global prevalence of oral pigmentation and its related factors: a systematic review and meta-analysis. J Stomatol Oral Max Surg. 2022;123:e411–24.

62. Tsimpiris A, Tsolianos I, Grigoriadis A, Moschos I, Goulis DG, Kouklakis G. Association of Chronic Periodontitis with Helicobacter pylori Infection in Stomach or Mouth: A Systematic Review and Meta-Analysis. Eur J Dent. 2022. https://doi.org/10.1055/s-0042-1756690.

63. Shetty A, Dixit U, Kirubakaran R. Prevalence of molar incisor hypomineralization in India: A systematic review and meta-analysis. J Indian Soc Pedodontics Prev Dent. 2022;40:356–67.

64. Reis PHF, Laxe LAC, Lacerda-Santos R, Münchow EA. Distribution of anxiety and depression among different subtypes of temporomandibular disorder: A systematic review and meta-analysis. J Oral Rehabil. 2022;49:754–67.

65. Sabbagh S, Mohammadi-Nasrabadi F, Ravaghi V, Azadi Mood K, Sarraf Shirazi A, Abedi A-S, et al. Food insecurity and dental caries prevalence in children and adolescents: A systematic review and meta-analysis. Int J Paediatr Dent. 2022. https://doi.org/10.1111/ipd.13041.

66. Bai X, Cui C, Yin J, Li H, Gong Q, Wei B, et al. The association between oral hygiene and head and neck cancer: a meta-analysis. Acta Odontol Scand. 2022. https://doi.org/10.1080/00016357.2022.2158129.

67. McCarra C, Olegário IC, O’Connell AC, Leith R. Prevalence of hypomineralised second primary molars (HSPM): A systematic review and meta-analysis. Int J Paediatr Dent. 2022;32:367–82.

68. Alves APNN, de Paula DS, Lima LVR, Dantas TS, Mota MRL, Sousa FB, et al. Is immunohistochemistry more sensitive than hematoxylin-eosin staining for identifying perineural or lymphovascular invasion in oral squamous cell carcinoma? A systematic review and meta-analysis. Med Oral Patol Oral Cir Bucal. 2022;27:e238–47.

69. Santos PS, Barasuol JC, Moccelini BS, Magno MB, Bolan M, Martins-Junior PA, et al. Prevalence of toothache and associated factors in children and adolescents: a systematic review and meta-analysis. Clin Oral Invest. 2022;26:1105–19.

70. Trivedi A, Agarwal S, Gupta I, Goswami R, Mowar A. A Comparative Evaluation of Malocclusion and Associated Risk Factors in Patients Suffering with Temporomandibular Disorders: A Systematic Review and Meta-analysis of Observational Studies. European J Gen Dent. 2022;11:149–57.

71. Moro JDS, Soares JP, Massignan C, Oliveira LB, Ribeiro DM, Cardoso M, et al. BURNOUT SYNDROME AMONG DENTISTS: A SYSTEMATIC REVIEW AND META-ANALYSIS. J Evid-Based Dent Pract. 2022;22.

72. Kesharani P, Kansara P, Kansara T, Kini A, Bhat R, Shetty P, et al. Is periodontitis a risk factor for lung cancer? A meta-analysis and detailed review of mechanisms of association. Contemp Clin Dent. 2022;13:297–306.

73. Gaba FI, Gonzalez RC, Martïnez RG. The Role of Oral Fusobacterium nucleatum in Female Breast Cancer: A Systematic Review and Meta-Analysis. Int J Dent. 2022;2022.

74. Moltajaei MH, Mehrbani SP, Motahari P, Rezapour R. Clinicopathological and prognostic value of Ki-67 expression in oral malignant melanoma: A systematic review and meta-analysis. J Dent Res Dent Clin Dent Prospects. 2022;16:140–6.

75. Kammer PV, Moro JS, Soares JP, Massignan C, Phadraig CMG, Bolan M. Prevalence of tooth grinding in children and adolescents with neurodevelopmental disorders: A systematic review and meta-analysis. J Oral Rehabil. 2022;49:671–85.

76. Lorenzo-Pouso AI, Lafuente-Ibáñez de Mendoza I, Pérez-Sayáns M, Pérez-Jardón A, Chamorro-Petronacci CM, Blanco-Carrión A, et al. Critical update, systematic review, and meta-analysis of oral erythroplakia as an oral potentially malignant disorder. J Oral Pathol Med. 2022;51:585–93.

77. Yanushevich OO, Maev IV, Krikheli NI, Andreev DN, Lyamina SV, Sokolov FS, et al. Prevalence and Risk of Dental Erosion in Patients with Gastroesophageal Reflux Disease: A Meta-Analysis. Dentistry J. 2022;10.

78. Hui L, Hung KF, Bornstein MM, Leung YY. Assessment of the prevalence and length of the anterior and caudal loops of the mental nerve as anatomical variants of exiting the mandible at the mental foramen using cone-beam computed tomography: a systematic review and meta-analysis. Clin Oral Invest. 2022;26:6423–41.

79. Salari N, Darvishi N, Heydari M, Bokaee S, Darvishi F, Mohammadi M. Global prevalence of cleft palate, cleft lip and cleft palate and lip: A comprehensive systematic review and meta-analysis. J Stomatol Oral Max Surg. 2022;123:110–20.

80. Howe BJ, Pendleton C, Withanage MHH, Childs CA, Zeng E, van Wijk A, et al. Tooth Agenesis Patterns in Orofacial Clefting Using Tooth Agenesis Code: A Meta-Analysis. Dentistry J. 2022;10.

81. Hadilou M, Gholami L, Ghojazadeh M, Emadi N. Prevalence and extension of the anterior loop of the mental nerve in different populations and CBCT imaging settings: A systematic review and meta-analysis. Imaging Sci Dent. 2022;52:141–53.

82. Martins-de-Barros AV, Anjos RSD, Silva CCG, Silva EDDOE, Araújo FADC, Carvalho MDV. Diagnostic accuracy of immunohistochemistry compared with molecular tests for detection of BRAF V600E mutation in ameloblastomas: Systematic review and meta-analysis. J Oral Pathol Med. 2022;51:223–30.

83. Moccelini BS, Santos PS, Barasuol JC, Magno MB, Bolan M, Maia LC, et al. Prevalence of sequelae after traumatic dental injuries to anterior primary teeth: A systematic review and meta-analysis. Dent Traumatol. 2022;38:286–98.

84. Avrella MT, Zimmermann DR, Andriani JSP, Santos PS, Barasuol JC. Prevalence of anterior open bite in children and adolescents: a systematic review and meta-analysis. Eur Arch Paediatr Dent. 2022;23:355–64.

85. Garot E, Rouas P, Somani C, Taylor GD, Wong F, Lygidakis NA. An update of the aetiological factors involved in molar incisor hypomineralisation (MIH): a systematic review and meta-analysis. Eur Arch Paediatr Dent. 2022;23:23–38.

86. Nogueira-Reis F, de Oliveira Reis L, Fontenele RC, Freitas DQ, Tabchoury CPM. Prevalence and features of elongated styloid process on imaging studies: a systematic review and meta-analysis. Clin Oral Invest. 2022;26:1199–215.

87. Rosário-dos-Santos HL, Miranda SS, Gomes-Filho IS, Cruz SSD, Figueiredo ACMG, Souza ES, et al. Periodontitis severity relationship with metabolic syndrome: A systematic review with meta-analysis. Oral Dis. 2022. https://doi.org/10.1111/odi.14428.

88. Shahi AK, Sharma S, Singh B, Tandon A, Kumar A, Chandra S. Assessment of Risk of Malignancy of Fine-needle Aspiration Cytology in Salivary Gland Lesions Using the Milan System for Reporting Salivary Gland Cytopathology Categorization: A Systematic Review and Meta-analysis. J Contemp Dental Pract. 2022;23:1039–56.

89. Khodadadi N, Khodadadi M, Zamani M. Is periodontitis associated with obstructive sleep apnea? A systematic review and meta-analysis. J Clini Exp Dent. 2022;14:359–65.

90. Li K, He W, Hua H. Characteristics of the psychopathological status of oral lichen planus: a systematic review and meta-analysis. Aust Dent J. 2022;67:113–24.

91. de Farias Gabriel A, Silveira FM, Curra M, Schuch LF, Wagner VP, Martins MAT, et al. Risk factors associated with the development of oral mucositis in pediatric oncology patients: Systematic review and meta-analysis. Oral Dis. 2022;28:1068–84.

92. Chen P, Hong F, Yu X. Prevalence of periodontal disease in pregnancy: A systematic review and meta-analysis. J Dent. 2022;125.

93. Morsy N, El Kateb M. Accuracy of intraoral scanners for static virtual articulation: A systematic review and meta-analysis of multiple outcomes. J Prosthet Dent. 2022. https://doi.org/10.1016/j.prosdent.2022.09.005.

94. Saikiran K, Gurunathan D, Elicherla S, Mallineni S, Nuvvula S. Pulp oxygen saturation measurement as a diagnostic tool for assessing pulp status in primary teeth: A systematic review and meta-analysis. J Indian Soc Pedodontics Prev Dent. 2022;40:349–55.

95. Adeoye J, Alade AA, Zhu W-Y, Wang W, Choi S-W, Thomson P. Efficacy of hypermethylated DNA biomarkers in saliva and oral swabs for oral cancer diagnosis: Systematic review and meta-analysis. Oral Dis. 2022;28:541–58.

96. Shan S, Zhong S, Li J, Wang T. Systematic review and meta-analysis of mandibular canal variations on cone beam computed tomography. Oral Radiol. 2022;38:445–51.

97. Pereira RDPL, de Oliveira JMD, Pauletto P, Munhoz EDA, Silva Guerra EN, Massignan C, et al. Worldwide prevalence of geographic tongue in adults: A systematic review and meta-analysis. Oral Dis. 2022. https://doi.org/10.1111/odi.14397.

98. Huang L, Pei T, Wu G, Liu J, Pan W, Pan X. Circular RNAs as a Diagnostic Biomarker in Oral Squamous Cell Carcinoma: A Meta-Analysis. J Oral Maxillofac Surg. 2022;80:756–66.

99. Réus JC, Polmann H, Souza BDM, Flores-Mir C, Gonçalves DAG, de Queiroz LP, et al. Association between primary headaches and temporomandibular disorders: A systematic review and meta-analysis. J Am Dent Assoc. 2022;153:120-131.e6.

100. Fell M, Dack K, Chummun S, Sandy J, Wren Y, Lewis S. Maternal Cigarette Smoking and Cleft Lip and Palate: A Systematic Review and Meta-Analysis. Cleft Palate-Craniofac J. 2022;59:1185–200.

101. Mohideen K, Krithika C, Jeddy N, Shamsuddin S, Basheer SA, Sainudeen S, et al. Depleting levels of endogenous anti-oxidant superoxide dismutase in oral sub-mucous fibrosis: A systematic review and meta-analysis: Superoxide dismutase and oral sub-mucous fibrosis. J Oral Biol Craniofac Res. 2022;12:343–51.

102. Cruz PV, Souza-Oliveira AC, Notaro SQ, Occhi-Alexandre IGP, Maia RM, De Luca Canto G, et al. Prevalence of ankyloglossia according to different assessment tools: A meta-analysis. J Am Dent Assoc. 2022;153:1026-1040.e31.

103. Wu S, Zhang W, Yan J, Noma N, Young A, Yan Z. Worldwide prevalence estimates of burning mouth syndrome: A systematic review and meta-analysis. Oral Dis. 2022;28:1431–40.

104. Rozema R, Doff MHJ, Delli K, Spijkervet FKL, van Minnen B. Diagnostic accuracy of physical examination findings for midfacial fractures: a systematic review and meta-analysis. Clin Oral Invest. 2022;26:3405–27.

105. Andonovski M-E, Antonarakis GS. Autism spectrum disorder and dentoalveolar trauma: A systematic review and meta-analysis. J Stomatol Oral Max Surg. 2022;123:e858–64.

106. Muinelo-Lorenzo J, Rodríguez-Pato R, Martínez-Reglero C, Salgado-Barreira A, Suárez-Cunqueiro MM. Detection of the Accessory Mental Foramina on Human Mandibles Using Cone-beam Computed Tomography: A Systematic Review and Meta-analysis. J Endod. 2021;47:1215–28.

107. Dos Santos ES, Normando AGC, Scarini JF, Crescencio LR, de Lima-Souza RA, Mariano FV, et al. Diagnostic and prognostic value of miRNAs on salivary gland tumors: a systematic review and meta-analysis. Oral Maxillofac Surg. 2021;25:445–56.

108. Buenahora MR, Peraza-L A, Díaz-Báez D, Bustillo J, Santacruz I, Trujillo TG, et al. Diagnostic accuracy of clinical visualization and light-based tests in precancerous and cancerous lesions of the oral cavity and oropharynx: a systematic review and meta-analysis. Clin Oral Invest. 2021;25:4145–59.

109. Moffa A, Giorgi L, Costantino A, De Benedetto L, Cassano M, Spriano G, et al. Accuracy of autofluorescence and chemiluminescence in the diagnosis of oral Dysplasia and Carcinoma: A systematic review and Meta-analysis. Oral Oncol. 2021;121.

110. Yuan H, Qiu J, Zhang T, Wu X, Zhou J, Park S. Quantitative changes of Veillonella, Streptococcus, and Neisseria in the oral cavity of patients with recurrent aphthous stomatitis: A systematic review and meta-analysis. Arch Oral Biol. 2021;129.

111. Uribe SE, Innes N, Maldupa I. The global prevalence of early childhood caries: A systematic review with meta-analysis using the WHO diagnostic criteria. Int J Paediatr Dent. 2021;31:817–30.

112. Lafuente Ibáñez de Mendoza I, Lorenzo Pouso AI, Aguirre Urízar JM, Barba Montero C, Blanco Carrión A, Gándara Vila P, et al. Malignant development of proliferative verrucous/multifocal leukoplakia: A critical systematic review, meta-analysis and proposal of diagnostic criteria. J Oral Pathol Med. 2022;51:30–8.

113. Zhang X, Gu H, Xie S, Su Y. Periodontitis in patients with psoriasis: A systematic review and meta-analysis. Oral Dis. 2022;28:33–43.

114. Sethi S, Ali A, Ju X, Antonsson A, Logan R, Canfell K, et al. A systematic review and meta-analysis of the prevalence of human papillomavirus infection in Indigenous populations – A Global Picture. J Oral Pathol Med. 2021;50:843–54.

115. Yuwanati M, Ramadoss R, Kudo Y, Ramani P, Senthil Murugan M. Prevalence of oral submucous fibrosis among areca nut chewers: A systematic review and meta-analysis. Oral Dis. 2023;29:1920–6.

116. Alawaji YN, Alshammari A, Aleksejuniene J. Accuracy of Estimating Periodontitis and Its Risk Association Using Partial-Mouth Recordings for Surveillance Studies: A Systematic Review and Meta-Analysis. Int J Dent. 2022;2022.

117. Barbosa DAF, Mesquita LR, Borges MMC, de Mendonça DS, de Carvalho FSR, Kurita LM, et al. Mental Foramen and Anterior Loop Anatomic Characteristics: A Systematic Review and Meta-analysis of Cross-sectional Imaging Studies. J Endod. 2021;47:1829-1843.e1.

118. Baima G, Corana M, Iaderosa G, Romano F, Citterio F, Meoni G, et al. Metabolomics of gingival crevicular fluid to identify biomarkers for periodontitis: A systematic review with meta-analysis. J Periodontal Res. 2021;56:633–45.

119. Gadicherla S, Pentapati K-C, Rustaqi N, Singh A, Smriti K. Diagnostic accuracy of ultrasonography for the assessment of maxillofacial fractures: A meta-analysis. J Intl Soc Prev Community Dent. 2021;11:503–9.

120. Nath S, Poirier B, Ju X, Kapellas K, Haag D, Jamieson L. Periodontal disease inequities among Indigenous populations: A systematic review and meta-analysis. J Periodontal Res. 2022;57:11–29.

121. de Oliveira JMD, Pauletto P, Werlich MO, Massignan C, Lehmkuhl KM, Porfírio GJM, et al. Prevalence of orofacial injuries in wheeled non-motor sports athletes: A systematic review and meta-analysis. Dent Traumatol. 2021;37:546–56.

122. de Paula DS, Nóbrega Malta CE, de Brito WH, Mota Lemos JV, Cetira Fillho EL, Gurgel Costa FW, et al. Prevalence of malignant neoplastic oral lesions among children and adolescents: A systematic review and meta-analysis. Int J Paediatr Dent. 2021;31:504–19.

123. Ramos-García P, González-Moles MÁ, Mello FW, Bagan JV, Warnakulasuriya S. Malignant transformation of oral proliferative verrucous leukoplakia: A systematic review and meta-analysis. Oral Dis. 2021;27:1896–907.

124. Schwendicke F, Chaurasia A, Arsiwala L, Lee J-H, Elhennawy K, Jost-Brinkmann P-G, et al. Deep learning for cephalometric landmark detection: systematic review and meta-analysis. Clin Oral Invest. 2021;25:4299–309.

125. Jabbarzadeh M, Hamblin MR, Pournaghi-Azar F, Saatloo MV, Kouhsoltani M, Vahed N. Ki-67 expression as a diagnostic biomarker in odontogenic cysts and tumors: A systematic review and meta-analysis. J Dent Res Dent Clin Dent Prospects. 2021;15:66–75.

126. Boustedt K, Dahlgren J, Roswall J, Twetman S. Is the mode of childbirth delivery linked to the prevalence of early childhood caries? A systematic review and meta-analysis. Eur Arch Paediatr Dent. 2021;22:765–72.

127. Usha G, Muddappa SC, Venkitachalam R, Singh V P P, Rajan RR, Ravi AB. Variations in root canal morphology of permanent incisors and canines among Asian population: A systematic review and meta-analysis. J Oral Biosci. 2021;63:337–50.

128. Fontenele RC, Machado AH, de Oliveira Reis L, Freitas DQ. Influence of metal artefact reduction tool on the detection of vertical root fractures involving teeth with intracanal materials in cone beam computed tomography images: A systematic review and meta-analysis. Int Endod J. 2021;54:1769–81.

129. Zhang S, Zhu J, Zhu Y, Zhang X, Wu R, Li S, et al. Oral manifestations of patients with systemic sclerosis: a meta-analysis for case-controlled studies. BMC Oral Health. 2021;21.

130. Kalogirou E-M, Thermos G, Zogopoulos V, Foutadakis S, Michalopoulos I, Agelopoulos M, et al. The immunohistochemical profile of basal cell nevus syndrome–associated and sporadic odontogenic keratocysts: a systematic review and meta-analysis. Clin Oral Invest. 2021;25:3351–67.

131. Wang X, Shujaat S, Shaheen E, Jacobs R. Accuracy of desktop versus professional 3D printers for maxillofacial model production. A systematic review and meta-analysis. J Dent. 2021;112.

132. de Sá Cavalcante D, de Barros Silva PG, Carvalho FSR, Quidute ARP, Kurita LM, Cid AMPL, et al. Is jaw fractal dimension a reliable biomarker for osteoporosis screening? A systematic review and meta-analysis of diagnostic test accuracy studies. Dentomaxillofac Radiol. 2022;51.

133. Lima LJS, Ramos-Jorge ML, Soares MEC. Prenatal, perinatal and postnatal events associated with hypomineralized second primary molar: a systematic review with meta-analysis. Clin Oral Invest. 2021;25:6501–16.

134. Abdalla-Aslan R, Shilo D, Nadler C, Eran A, Rachmiel A. Diagnostic correlation between clinical protocols and magnetic resonance findings in temporomandibular disorders: A systematic review and meta-analysis. J Oral Rehabil. 2021;48:955–67.

135. Maria de França G, Andrade ACDM, Felix FA, da Silva WR, Almeida DRDMF, Leite RB, et al. Survival-related epithelial-mesenchymal transition proteins in oropharyngeal squamous cell carcinoma: A systematic review and meta-analysis. Arch Oral Biol. 2021;131.

136. Cavalcante DKF, Veloso SRM, Durão MDA, Melo VDC, Monteiro GQDM, Porto GG. Do Helmet Use and Type Influence Facial Trauma Occurrence and Severity in Motorcyclists? A Systematic Review and Meta-analysis. J Oral Maxillofac Surg. 2021;79:1492–506.

137. PradeepKumar AR, Shemesh H, Nivedhitha MS, Hashir MMJ, Arockiam S, Uma Maheswari TN, et al. Diagnosis of Vertical Root Fractures by Cone-beam Computed Tomography in Root-filled Teeth with Confirmation by Direct Visualization: A Systematic Review and Meta-Analysis. J Endod. 2021;47:1198–214.

138. Varela-Centelles P, Gonzalez-Moles MÁ, Seoane-Romero J, Leira-Feijoo Y, Takkouche B, Seoane-Romero JM. Immunohistochemical analysis of epithelium adjacent to lip cancer: A meta-analysis. Oral Dis. 2022;28:57–65.

139. Roithmann CC, Silva CAGD, Pattussi MP, Grossi ML. Subjective sleep quality and temporomandibular disorders: Systematic literature review and meta-analysis. J Oral Rehabil. 2021;48:1380–94.

140. Kapor S, Rankovic MJ, Khazaei Y, Crispin A, Schüler I, Krause F, et al. Systematic review and meta-analysis of diagnostic methods for occlusal surface caries. Clin Oral Invest. 2021;25:4801–15.

141. Devanna R, Felemban NH, Althomali Y, Battepati PM, Ali Alfawzan A, Gupta P. Prevalence of malocclusion among children of the Kingdom of Saudi Arabia – A systematic review and meta-analysis. Saudi Dent J. 2021;33:826–34.

142. Yang J, Wang Z-Y, Huang L, Yu T-L, Wan S-Q, Song J, et al. Do betel quid and areca nut chewing deteriorate prognosis of oral cancer? A systematic review, meta-analysis, and research agenda. Oral Dis. 2021;27:1366–75.

143. Barbosa DAF, de Mendonça DS, de Carvalho FSR, Kurita LM, de Barros Silva PG, Neves FS, et al. Systematic review and meta-analysis of lingual foramina anatomy and surgical-related aspects on cone-beam computed tomography: a PROSPERO-registered study. Oral Radiol. 2022;38.

144. Foros P, Oikonomou E, Koletsi D, Rahiotis C. Detection Methods for Early Caries Diagnosis: A Systematic Review and Meta-Analysis. Caries Res. 2021;55:247–59.

145. Venkat M, Janakiram C, Ramanarayanan V. Prevalence of tooth mortality among adults in India: A systematic review and meta-analysis. Contemp Clin Dent. 2021;12:339–45.

146. Mohideen K, Krithika C, Jeddy N, Balakrishnan T, Bharathi R, Sankari S. A meta-analysis of oral squamous cell carcinoma in young adults with a comparison to the older group patients (2014-2019). Contemp Clin Dent. 2021;12:213–21.

147. Monteiro L, Mariano LC, Warnakulasuriya S. Podoplanin could be a predictive biomarker of the risk of patients with oral leukoplakia to develop oral cancer: A systematic review and meta-analysis. Oral Dis. 2022. https://doi.org/10.1111/odi.14378.

148. Batistella EÂ, Sabino da Silva R, Rivero ERC, Silva CAB. Prevalence of oral mucosal lesions in patients with pemphigus vulgaris: A systematic review and meta-analysis. J Oral Pathol Med. 2021;50:750–7.

149. Tiwari P, Bera RN, Kanojia S, Chauhan N, Hirani MS. Assessing the optimal imaging modality in the diagnosis of jaw osteomyelitis. A meta-analysis. Br J Oral Maxillofac Surg. 2021;59:982–92.

150. Felin GC, Tagliari CVDC, Agostini BA, Collares K. Prevalence of psychological disorders in patients with temporomandibular disorders: A systematic review and meta-analysis. J Prosthet Dent. 2022. https://doi.org/10.1016/j.prosdent.2022.08.002.

151. Janjic Rankovic M, Kapor S, Khazaei Y, Crispin A, Schüler I, Krause F, et al. Systematic review and meta-analysis of diagnostic studies of proximal surface caries. Clin Oral Invest. 2021;25:6069–79.

152. Hussain SB, Leira Y, Zehra SA, Botelho J, Machado V, Ciurtin C, et al. Periodontitis and Systemic Lupus Erythematosus: A systematic review and meta-analysis. J Periodontal Res. 2022;57:1–10.

153. Yadav VS, Gumber B, Makker K, Gupta V, Tewari N, Khanduja P, et al. Global prevalence of gingival recession: A systematic review and meta-analysis. Oral Dis. 2022. https://doi.org/10.1111/odi.14289.

154. Anirudhan S, Suneelkumar C, Uppalapati H, Anumula L, Kirubakaran R. Detection of second mesiobuccal canals in maxillary first molars of the Indian population - a systematic review and meta-analysis. Evid-based Dent. 2022. https://doi.org/10.1038/s41432-022-0233-3.

155. Molek M, Florenly F, Lister INE, Wahab TA, Lister C, Fioni F. Xerostomia and hyposalivation in association with oral candidiasis: a systematic review and meta-analysis. Evid-based Dent. 2022. https://doi.org/10.1038/s41432-021-0210-2.

156. Gambin DJ, Vitali FC, De Carli JP, Mazzon RR, Gomes BPFA, Duque TM, et al. Prevalence of red and orange microbial complexes in endodontic-periodontal lesions: a systematic review and meta-analysis. Clin Oral Invest. 2021;25:6533–46.

157. Aung NM, Myint KK. Three-Rooted Permanent Mandibular First Molars: A Meta-Analysis of Prevalence. Int J Dent. 2022;2022.

158. Roy S, Raj D, Ghosh S, Majumdar S, Das S. Comparative Accuracy of Panoramic Radiograph and Conebeam Computed Tomography Images in Identification and Measurement of the Anterior Loop of Mental Nerve: A Metaanalysis and Review of the Literature. Int J Prosthodont Restor Dent. 2021;11:132–7.

159. Nath S, Poirier BF, Ju X, Kapellas K, Haag DG, Ribeiro Santiago PH, et al. Dental Health Inequalities among Indigenous Populations: A Systematic Review and Meta-Analysis. Caries Res. 2021;55:268–87.

160. Siddiqui AA, Alshammary F, Mulla M, Al-Zubaidi SM, Afroze E, Amin J, et al. Prevalence of dental caries in Pakistan: a systematic review and meta-analysis. BMC Oral Health. 2021;21.

161. Liu D, Xin Z, Guo S, Li S, Cheng J, Jiang H. Blood and Salivary MicroRNAs for Diagnosis of Oral Squamous Cell Carcinoma: A Systematic Review and Meta-Analysis. J Oral Maxillofac Surg. 2021;79:1082.e1-1082.e13.

162. Poletto AG, Mello FW, Melo G, Rivero ERC. Prevalence of mucoepidermoid carcinoma among intraoral minor salivary gland tumors: A systematic review and meta-analysis. J Oral Pathol Med. 2020;49:720–6.

163. Moosazadeh M, Shafaroudi AM, Gorji NE, Barzegari S, Nasiri P. Prevalence of oral lesions in patients with AIDS: a systematic review and meta-analysis. Evid-based Dent. 2021. https://doi.org/10.1038/s41432-021-0209-8.

164. Narayanan SP, Rath H, Panda A, Mahapatra S, Kader RH. Prevalence, Trends, and Associated Risk Factors of Traumatic Dental Injury among Children and Adolescents in India: A Systematic Review and Meta-analysis. J Contemp Dental Pract. 2021;22:1206–24.

165. Lorenzo-Pouso AI, Castelo-Baz P, Rodriguez-Zorrilla S, Pérez-Sayáns M, Vega P. Association between periodontal disease and inflammatory bowel disease: a systematic review and meta-analysis. Acta Odontol Scand. 2021;79:344–53.

166. Patnana AK, Chugh A, Chugh VK, Kumar P, Vanga NRV, Singh S. The prevalence of traumatic dental injuries in primary teeth: A systematic review and meta-analysis. Dent Traumatol. 2021;37:383–99.

167. Grisolia BM, dos Santos APP, Dhyppolito IM, Buchanan H, Hill K, Oliveira BH. Prevalence of dental anxiety in children and adolescents globally: A systematic review with meta-analyses. Int J Paediatr Dent. 2021;31:168–83.

168. Campos DS, de Araújo Ferreira Muniz I, de Souza Villarim NL, Ribeiro ILA, Batista AUD, Bonan PRF, et al. Is there an association between rheumatoid arthritis and bone changes in the temporomandibular joint diagnosed by cone-beam computed tomography? A systematic review and meta-analysis. Clin Oral Invest. 2021;25:2449–59.

169. Valesan LF, Da-Cas CD, Réus JC, Denardin ACS, Garanhani RR, Bonotto D, et al. Prevalence of temporomandibular joint disorders: a systematic review and meta-analysis. Clin Oral Invest. 2021;25:441–53.

170. Dehesa-Santos A, Iber-Diaz P, Iglesias-Linares A. Genetic factors contributing to skeletal class III malocclusion: a systematic review and meta-analysis. Clin Oral Invest. 2021;25:1587–612.

171. Zheng D-X, Kang X-N, Wang Y-X, Huang Y-N, Pang C-F, Chen Y-X, et al. Periodontal disease and emotional disorders: A meta-analysis. J Clin Periodontol. 2021;48:180–204.

172. Mishra SK, Nahar R, Gaddale R, Chowdhary R. Identification of anterior loop in different populations to avoid nerve injury during surgical procedures—a systematic review and meta-analysis. Oral Maxillofac Surg. 2021;25:159–74.

173. Burr MR, Naze GS, Shaffer SM, Emerson AJ. The role of sleep dysfunction in temporomandibular onset and progression: A systematic review and meta-analyses. J Oral Rehabil. 2021;48:183–94.

174. Idrees M, Kujan O, Shearston K, Farah CS. Oral lichen planus has a very low malignant transformation rate: A systematic review and meta-analysis using strict diagnostic and inclusion criteria. J Oral Pathol Med. 2021;50:287–98.

175. Natanasabapathy V, Arul B, Mishra A, Varghese A, Padmanaban S, Elango S, et al. Ultrasound imaging for the differential diagnosis of periapical lesions of endodontic origin in comparison with histopathology – a systematic review and meta-analysis. Int Endod J. 2021;54:693–711.

176. Pentapati KC, Yeturu SK, Siddiq H. Global and regional estimates of dental pain among children and adolescents—systematic review and meta-analysis. Eur Arch Paediatr Dent. 2021;22.

177. Knorst JK, Sfreddo CS, de F. Meira G, Zanatta FB, Vettore MV, Ardenghi TM. Socioeconomic status and oral health-related quality of life: A systematic review and meta-analysis. Community Dent Oral Epidemiol. 2021;49:95–102.

178. Huang S, Zhu Y, Cai H, Zhang Y, Hou J. Impact of lymphovascular invasion in oral squamous cell carcinoma: A meta-analysis. Oral Surg Oral Med Oral Pathol Oral Radiol. 2021;131:319-328.e1.

179. Bronzato JD, Bomfim RA, Hayasida GZP, Cúri M, Estrela C, Paster BJ, et al. Analysis of microorganisms in periapical lesions: A systematic review and meta-analysis. Arch Oral Biol. 2021;124.

180. Rodriguez-Archilla A, Fuentes-Perez C. Clinicopathological Parameters Related To Malignant Transformation of Oral Leukoplakia: A Meta-analysis. Cumhuriyet Dent J. 2021;24:198–205.

181. Moosazadeh M, Gorji NE, Nasiri P, Shafaroudi AM. Comparison of decayed, missing, filled teeth index between thalassemia major patients and control group in Iran: a systematic review and meta-analysis. BDJ Open. 2020;6.

182. Borsetto D, Fussey J, Fabris L, Bandolin L, Gaudioso P, Phillips V, et al. HCV infection and the risk of head and neck cancer: A meta-analysis. Oral Oncol. 2020;109.

183. Janakiram C, Mehta A, Venkitachalam R. Prevalence of periodontal disease among adults in India: A systematic review and meta-analysis. J Oral Biol Craniofac Res. 2020;10:800–6.

184. Dos Santos ES, Ramos JC, Normando AGC, Leme AFP. Prognostic value of the immunohistochemical expression of vascular endothelial growth factors in malignant salivary gland neoplasms: A systematic review and meta-analysis. Med Oral Patol Oral Cir Bucal. 2021;26:e126–35.

185. Kazeminia M, Abdi A, Shohaimi S, Jalali R, Vaisi-Raygani A, Salari N, et al. Dental caries in primary and permanent teeth in children’s worldwide, 1995 to 2019: A systematic review and meta-analysis. Head Face Med. 2020;16.

186. Oliveira Werlich M, Honnef LR, Silva Bett JV, Domingos FL, Pauletto P, Dulcineia Mendes de Souza B, et al. Prevalence of dentofacial injuries in contact sports players: A systematic review and meta-analysis. Dent Traumatol. 2020;36:477–88.

187. Magnucki G, Mietling SVK. Four-Rooted Maxillary First Molars: A Systematic Review and Meta-Analysis. Int J Dent. 2021;2021.

188. Pandey P, Nandkeoliar T, Tikku AP, Singh D, Singh MK. Prevalence of dental caries in the Indian population: A systematic review and meta-analysis. J Intl Soc Prev Community Dent. 2021;11:256–65.

189. Xuan K, Jha AR, Zhao T, Uy JP, Sun C. Is periodontal disease associated with increased risk of colorectal cancer? A meta-analysis. Int J Dent Hyg. 2021;19:50–61.

190. Eidt G, Waltermann EDM, Hilgert JB, Arthur RA. Candida and dental caries in children, adolescents and adults: A systematic review and meta-analysis. Arch Oral Biol. 2020;119.

191. de la Cour CD, Sperling CD, Belmonte F, Syrjänen S, Kjaer SK. Human papillomavirus prevalence in oral potentially malignant disorders: Systematic review and meta-analysis. Oral Dis. 2021;27:431–8.

192. Balachandran P, Janakiram C. Prevalence of malocclusion among 8–15 years old children, India – A systematic review and meta-analysis. J Oral Biol Craniofac Res. 2021;11:192–9.

193. Wang J, Yang X, Zou X, Zhang Y, Wang J, Wang Y. Relationship between periodontal disease and lung cancer: A systematic review and meta-analysis. J Periodontal Res. 2020;55:581–93.

194. Campos LGN, Pedrosa BH, Cavalcanti RVA, Stechman-Neto J, Gadotti IC, de Araujo CM, et al. Prevalence of temporomandibular disorders in musicians: A systematic review and meta-analysis. J Oral Rehabil. 2021;48:632–42.

195. Aung NM, Myint KK. Diagnostic Accuracy of CBCT for Detection of Second Canal of Permanent Teeth: A Systematic Review and Meta-Analysis. Int J Dent. 2021;2021.

196. de Lima AKA, Amorim dos Santos J, Stefani CM, Almeida de Lima A, Damé-Teixeira N. Diabetes mellitus and poor glycemic control increase the occurrence of coronal and root caries: a systematic review and meta-analysis. Clin Oral Invest. 2020;24:3801–12.

197. Thanh MTG, Toan NV, Toan DTT, Thang NP, Dong NQ, Dung NT, et al. Diagnostic value of fluorescence methods, visual inspection and photographic visual examination in initial caries lesion: A systematic review and meta-analysis. Dentistry J. 2021;9.

198. Decaup P-H, Garot E, Rouas P. Prevalence of talon cusp: Systematic literature review, meta-analysis and new scoring system. Arch Oral Biol. 2021;125.

199. Cho JH, Lim YC. Prognostic impact of regulatory T cell in head and neck squamous cell carcinoma: A systematic review and meta-analysis. Oral Oncol. 2021;112.

200. Darley RM, Fernandes e Silva C, Costa FDS, Xavier CB, Demarco FF. Complications and sequelae of concussion and subluxation in permanent teeth: A systematic review and meta-analysis. Dent Traumatol. 2020;36:557–67.

201. Singh A, Purohit BM. Malnutrition and Its Association with Dental Caries in the Primary and Permanent Dentition: A Systematic Review and Meta-Analysis. Pediatr Dent. 2020;42:418–26.

202. Silva MAG, Pantoja LLQ, Dutra-Horstmann KL, Valladares-Neto J, Wolff FL, Porporatti AL, et al. Prevalence of degenerative disease in temporomandibular disorder patients with disc displacement: A systematic review and meta-analysis. J Cranio-Maxillofac Surg. 2020;48:942–55.

203. Savian CM, Bolsson GB, Botton G, Antoniazzi RP, de Oliveira Rocha R, Zanatta FB, et al. Do breastfed children have a lower chance of developing mouth breathing? A systematic review and meta-analysis. Clin Oral Invest. 2021;25:1641–54.

204. Ramos-Garcia P, Roca-Rodriguez MDM, Aguilar-Diosdado M, Gonzalez-Moles MA. Diabetes mellitus and oral cancer/oral potentially malignant disorders: A systematic review and meta-analysis. Oral Dis. 2021;27:404–21.

205. Cao C, Gan X, He Y, Su Y, Liu Z, Hu X, et al. Diagnostic efficacy of PET-CT, CT, and MRI in preoperative assessment of mandibular invasion caused by head and neck cancer: A systematic review and meta-analysis. Oral Oncol. 2021;116.

206. Pina GDMS, Mota Carvalho R, Silva BSDF, Almeida FT. Prevalence of hyposalivation in older people: A systematic review and meta-analysis. Gerodontology. 2020;37:317–31.

207. Campello CP, Pellizzer EP, Vasconcelos BCDE, Moraes SLD, Lemos CAA, Muniz MTC. Evaluation of IL-6 levels and +3954 polymorphism of IL-1β in burning mouth syndrome: A systematic review and meta-analysis. J Oral Pathol Med. 2020;49:961–8.

208. González-Moles MÁ, Warnakulasuriya S, González-Ruiz I, González-Ruiz L, Ayén Á, Lenouvel D, et al. Worldwide prevalence of oral lichen planus: A systematic review and meta-analysis. Oral Dis. 2021;27:813–28.

209. Ashraf S, Al-Maweri SA, Alaizari N, Umair A, Ariffin Z, Alhajj MN, et al. The association between Epstein-Barr virus and oral lichen planus: A systematic review and meta-analysis. J Oral Pathol Med. 2020;49:969–76.

210. Tibúrcio-Machado CS, Michelon C, Zanatta FB, Gomes MS, Marin JA, Bier CA. The global prevalence of apical periodontitis: a systematic review and meta-analysis. Int Endod J. 2021;54:712–35.

211. Silva VKS, Vieira WA, Bernardino ÍM, Travençolo BAN, Bittencourt MAV, Blumenberg C, et al. Accuracy of computer-assisted image analysis in the diagnosis of maxillofacial radiolucent lesions: A systematic review and meta-analysis. Dentomaxillofac Radiol. 2020;49.

212. Martins JNR, Marques D, Silva EJNL, Caramês J, Mata A, Versiani MA. Prevalence of C-shaped canal morphology using cone beam computed tomography – a systematic review with meta-analysis. Int Endod J. 2019;52:1556–72.

213. Bouziane A, Hamdoun R, Abouqal R, Ennibi O. Global prevalence of aggressive periodontitis: A systematic review and meta-analysis. J Clin Periodontol. 2020;47:406–28.

214. Doriguêtto PVT, Carrada CF, Scalioni FAR, Abreu LG, Devito KL, Paiva SM, et al. Malocclusion in children and adolescents with Down syndrome: A systematic review and meta-analysis. Int J Paediatr Dent. 2019;29:524–41.

215. Liu Y, Li R, Xue X, Xu T, Luo Y, Dong Q, et al. Periodontal disease and Helicobacter pylori infection in oral cavity: a meta-analysis of 2727 participants mainly based on Asian studies. Clin Oral Invest. 2020;24:2175–88.

216. Silva MCPMD, Lyra MCA, Almeida HCRD, Alencar Filho AVD, Heimer MV, Rosenblatt A. Caries experience in children and adolescents with Down Syndrome: A systematic review and meta-analysis. Arch Oral Biol. 2020;115.

217. Occhi-Alexandre IGP, Cruz PV, Bendo CB, Paiva SM, Pordeus IA, Martins CC. Prevalence of dental caries in preschool children born preterm and/or with low birth weight: A systematic review with meta-analysis of prevalence data. Int J Paediatr Dent. 2020;30:265–75.

218. Arias-Bujanda N, Regueira-Iglesias A, Balsa-Castro C, Nibali L, Donos N, Tomás I. Accuracy of single molecular biomarkers in saliva for the diagnosis of periodontitis: A systematic review and meta-analysis. J Clin Periodontol. 2020;47:2–18.

219. Imani MM, Safaei M, Lopez-Jornet P, Sadeghi M. A systematic review and meta-analysis on protective role of forkhead box E1 (FOXE1) polymorphisms in susceptibility to non-syndromic cleft lip/palate. Int Orthod. 2019;17:437–45.

220. Chinnasamy A, Moodie M. Prevalence of Undiagnosed Diabetes and Prediabetes in the Dental Setting: A Systematic Review and Meta-Analysis. Int J Dent. 2020;2020.

221. Lombardo G, Vena F, Negri P, Pagano S, Barilotti C, Paglia L, et al. Worldwide prevalence of malocclusion in the different stages of dentition: A systematic review and meta-analysis. Eur J Paediatr Dent. 2020;21:115–22.

222. Chisini LA, Cademartori MG, Francia A, Mederos M, Grazioli G, Conde MCM, et al. Is the use of Cannabis associated with periodontitis? A systematic review and meta-analysis. J Periodontal Res. 2019;54:311–7.

223. Limeira FIR, Yamauti M, Moreira AN, Galdino TM, de Magalhães CS, Abreu LG. Dental caries and developmental defects of enamel in individuals with chronic kidney disease: Systematic review and meta-analysis. Oral Dis. 2019;25:1446–64.

224. Gupta AA, Kheur S, Raj AT, Mahajan P. Association of Helicobacter pylori with oral potentially malignant disorders and oral squamous cell carcinoma—a systematic review and meta-analysis. Clin Oral Invest. 2020;24:13–23.

225. Martins JNR, Marques D, Leal Silva EJN, Caramês J, Mata A, Versiani MA. Influence of Demographic Factors on the Prevalence of a Second Root Canal in Mandibular Anterior Teeth – A Systematic Review and Meta-Analysis of Cross-Sectional Studies Using Cone Beam Computed Tomography. Arch Oral Biol. 2020;116.

226. Graizel D, Zlotogorski-Hurvitz A, Tsesis I, Rosen E, Kedem R, Vered M. Oral cancer-associated fibroblasts predict poor survival: Systematic review and meta-analysis. Oral Dis. 2020;26:733–44.

227. Alshammary F, Siddiqui AA, Al-Enizy AS, Almalaq SAS, Amin J, Rathore HA, et al. Prevalence of dental fluorosis in Saudi Arabia: A meta-analysis. Pesqui Bras Odontopediatria Clin Integr. 2020;20:1–10.

228. Skeie MS, Gil EG, Cetrelli L, Rosén A, Fischer J, Åstrøm AN, et al. Oral health in children and adolescents with juvenile idiopathic arthritis - A systematic review and meta-analysis. BMC Oral Health. 2019;19.

229. Bronzato JD, Bomfim RA, Edwards DH, Crouch D, Hector MP, Gomes BPFA. Detection of Fusobacterium in oral and head and neck cancer samples: A systematic review and meta-analysis. Arch Oral Biol. 2020;112.

230. González-Moles MÁ, Ruiz-Ávila I, González-Ruiz L, Ayén Á, Gil-Montoya JA, Ramos-García P. Malignant transformation risk of oral lichen planus: A systematic review and comprehensive meta-analysis. Oral Oncol. 2019;96:121–30.

231. Rapado-González O, Martínez-Reglero C, Salgado-Barreira A, López-López R, Suárez-Cunqueiro MM, Muinelo-Romay L. miRNAs in liquid biopsy for oral squamous cell carcinoma diagnosis: Systematic review and meta-analysis. Oral Oncol. 2019;99.

232. Lenouvel D, González-Moles MÁ, Ruiz-Ávila I, Gonzalez-Ruiz L, Gonzalez-Ruiz I, Ramos-García P. Prognostic and clinicopathological significance of PD-L1 overexpression in oral squamous cell carcinoma: A systematic review and comprehensive meta-analysis. Oral Oncol. 2020;106.

233. Pérez-de-Oliveira ME, Wagner VP, Araújo ALD, Martins MD, Santos-Silva AR, Bingle L, et al. Prognostic value of CRTC1-MAML2 translocation in salivary mucoepidermoid carcinoma: Systematic review and meta-analysis. J Oral Pathol Med. 2020;49:386–94.

234. Wang Y, Xing L, Yu H, Zhao L. Prevalence of dental caries in children and adolescents with type 1 diabetes: A systematic review and meta-analysis. BMC Oral Health. 2019;19.

235. Martins JNR, Marques D, Silva EJNL, Caramês J, Mata A, Versiani MA. Second mesiobuccal root canal in maxillary molars—A systematic review and meta-analysis of prevalence studies using cone beam computed tomography. Arch Oral Biol. 2020;113.

236. Zhang Y, Sun C, Song EJ, Liang M, Shi T, Min M, et al. Is periodontitis a risk indicator for gastrointestinal cancers? A meta-analysis of cohort studies. J Clin Periodontol. 2020;47:134–47.

237. Huang Z, Xie N, Liu H, Wan Y, Zhu Y, Zhang M, et al. The prognostic role of tumour-infiltrating lymphocytes in oral squamous cell carcinoma: A meta-analysis. J Oral Pathol Med. 2019;48:788–98.

238. Arraj GP, Rossi-Fedele G, Doğramacı EJ. The association of overjet size and traumatic dental injuries—A systematic review and meta-analysis. Dent Traumatol. 2019;35:217–32.

239. Otero Rey EM, Yáñez-Busto A, Rosa Henriques IF, López-López J, Blanco-Carrión A. Lichen planus and diabetes mellitus: Systematic review and meta-analysis. Oral Dis. 2019;25:1253–64.

240. Imani MM, Lopez-Jornet P, López EP-F, Ghanbari F, Sadeghi M. Association of Betaine-Homocysteine S-Methyl Transferase (rs3797546 and rs3733890) polymorphisms with non-syndromic cleft lip/palate: A meta-analysis. Int Orthod. 2019;17:643–51.

241. Toedtling V, Devlin H, Tickle M, O’Malley L. Prevalence of distal surface caries in the second molar among referrals for assessment of third molars: a systematic review and meta-analysis. Br J Oral Maxillofac Surg. 2019;57:505–14.

242. Azeredo F, Guimarães L, Luís W, Fialho S, Alves Antunes L, Antunes L. Estimated prevalence of dental caries in athletes: An epidemiological systematic review and meta-analysis. Indian J Dent Res. 2020;31:297–304.

243. Bohner L, Hanisch M, Chilvarquer I, Kleinheinz J, Tortamano P. Assessment of peri-implant buccal bone thickness using digital imaging techniques: A systematic review and meta-analysis. Open Dent J. 2020;14:150–60.

244. Kale S, Kakodkar P, Shetiya S, Rizwan S. Dental caries prevalence among 5- to 15-year-old children from SEAR countries of WHO: A systematic review and meta-analysis. Indian J Dent Res. 2019;30:937–47.

245. Pinto KP, Ferreira CM, Maia LC, Sassone LM, Fidalgo TKS, Silva EJNL. Does tobacco smoking predispose to apical periodontitis and endodontic treatment need? A systematic review and meta-analysis. Int Endod J. 2020;53:1068–83.

246. Silveira ALNDMES, Magno MB, Soares TRC. The relationship between special needs and dental trauma. A systematic review and meta-analysis. Dent Traumatol. 2020;36:218–36.

247. Santana T, Matuck B, Tenório JR, Braga MM. Can immunohistochemical biomarkers distinguish epithelial dysplasia degrees in actinic cheilitis? A systematic review and meta-analysis. Med Oral Patol Oral Cir Bucal. 2020;25:e106–16.

248. Hendra FN, Van Cann EM, Helder MN, Ruslin M, de Visscher JG, Forouzanfar T, et al. Global incidence and profile of ameloblastoma: A systematic review and meta-analysis. Oral Dis. 2020;26:12–21.

249. Polmann H, Melo G, Conti Réus J, Domingos FL, de Souza BDM, Padilha AC, et al. Prevalence of dentofacial injuries among combat sports practitioners: A systematic review and meta-analysis. Dent Traumatol. 2020;36:124–40.

250. Tewari N, Mathur VP, Siddiqui I, Morankar R, Verma AR, Pandey RM. Prevalence of traumatic dental injuries in India: A systematic review and meta-analysis. Indian J Dent Res. 2020;31:601–14.

251. Fatturi AL, Wambier LM, Chibinski AC, Assunção LRDS, Brancher JA, Reis A, et al. A systematic review and meta-analysis of systemic exposure associated with molar incisor hypomineralization. Community Dent Oral Epidemiol. 2019;47:407–15.

252. Xie C, Lin M, Yang H, Ren A. Prevalence of temporomandibular disorders and its clinical signs in Chinese students, 1979–2017: A systematic review and meta-analysis. Oral Dis. 2019;25:1697–706.

253. Dioguardi M, Di Gioia G, Caloro GA, Capocasale G, Zhurakivska K, Troiano G, et al. The association between tooth loss and Alzheimer’s disease: A systematic review with meta-analysis of case control studies. Dentistry J. 2019;7.

254. Arias-Bujanda N, Regueira-Iglesias A, Balsa-Castro C, Nibali L, Donos N, Tomás I. Accuracy of single molecular biomarkers in gingival crevicular fluid for the diagnosis of periodontitis: A systematic review and meta-analysis. J Clin Periodontol. 2019;46:1166–82.

255. He J, Chen X-F, Xu M-G, Zhao J. Relationship of programmed death ligand-1 expression with clinicopathological features and prognosis in patients with oral squamous cell carcinoma: A meta-analysis. Arch Oral Biol. 2020;114.

256. Oliveira LB, Massignan C, Oenning AC, Rovaris K, Bolan M, Porporatti AL, et al. Validity of micro-CT for in vitro caries detection: A systematic review and meta-analysis. Dentomaxillofac Radiol. 2020;49.

257. Kong X, Li H, Han Z. The diagnostic role of ultrasonography, computed tomography, magnetic resonance imaging, positron emission tomography/computed tomography, and real-time elastography in the differentiation of benign and malignant salivary gland tumors: a meta-analysis. Oral Surg Oral Med Oral Pathol Oral Radiol. 2019;128:431-443.e1.

258. Lin M, Xie C, Yang H, Wu C, Ren A. Prevalence of malocclusion in Chinese schoolchildren from 1991 to 2018: A systematic review and meta-analysis. Int J Paediatr Dent. 2020;30:144–55.

259. Qiao X, Liu W, Cao Y, Miao C, Yang W, Su N, et al. Performance of different imaging techniques in the diagnosis of head and neck cancer mandibular invasion: A systematic review and meta-analysis. Oral Oncol. 2018;86:150–64.

260. Moreira Falci SG, Duarte-Rodrigues L, Primo-Miranda EF, Furtado Gonçalves P, Lanza Galvão E. Association between epilepsy and oral maxillofacial trauma: A systematic review and meta-analysis. Spec Care Dent. 2019. https://doi.org/10.1111/scd.12398.

261. Mello FW, Melo G, Kammer PV, Speight PM, Rivero ERC. Prevalence of odontogenic cysts and tumors associated with impacted third molars: A systematic review and meta-analysis. J Cranio-Maxillofac Surg. 2019;47:996–1002.

262. Mottaghi A, Menéndez-Díaz I, Cobo JL, González-Serrano J, Cobo T. Is there a higher prevalence of tinnitus in patients with temporomandibular disorders? A systematic review and meta-analysis. J Oral Rehabil. 2019;46:76–86.

263. Su N, van Wijk AJ, Visscher CM, Lobbezoo F, van der Heijden GJMG. Diagnostic value of ultrasonography for the detection of disc displacements in the temporomandibular joint: a systematic review and meta-analysis. Clin Oral Invest. 2018;22:2599–614.

264. Schroder AGD, de Araujo CM, Guariza-Filho O, Flores-Mir C, de Luca Canto G, Porporatti AL. Diagnostic accuracy of panoramic radiography in the detection of calcified carotid artery atheroma: a meta-analysis. Clin Oral Invest. 2019. https://doi.org/10.1007/s00784-019-02880-6.

265. Glória JCR, Martins CC, Armond ACV, Galvão EL, Dos Santos CRR, Falci SGM. Third Molar and Their Relationship with Caries on the Distal Surface of Second Molar: A Meta-analysis. J Maxillofac Oral Surg. 2018;17:129–41.

266. Miri SS, Khademi A, Amirkhani Z, Amiri SM, Goodarzi M, Khazaei S. Prevalence of apical periodontitis in different communities: A meta-analysis. Iran Endod J. 2018;13:438–45.

267. Wang Y, Wang P, Andrukhov O, Wang T, Song S, Yan C, et al. Meta-analysis of the prognostic value of the neutrophil-to-lymphocyte ratio in oral squamous cell carcinoma. J Oral Pathol Med. 2018;47:353–8.

268. Petti S, Glendor U, Andersson L. World traumatic dental injury prevalence and incidence, a meta-analysis—One billion living people have had traumatic dental injuries. Dent Traumatol. 2018;34:71–86.

269. Schroder AGD, Guariza-Filho O, de Araujo CM, Ruellas AC, Tanaka OM, Porporatti AL. To what extent are impacted canines associated with root resorption of the adjacent tooth?: A systematic review with meta-analysis. J Am Dent Assoc. 2018;149:765-777.e8.

270. Souto-Souza D, da Consolação Soares ME, Rezende VS, de Lacerda Dantas PC, Galvão EL, Falci SGM. Association between developmental defects of enamel and celiac disease: A meta-analysis. Arch Oral Biol. 2018;87:180–90.

271. Mainkar A, Kim SG. Diagnostic Accuracy of 5 Dental Pulp Tests: A Systematic Review and Meta-analysis. J Endod. 2018;44:694–702.

272. Graziani F, Gennai S, Solini A, Petrini M. A systematic review and meta-analysis of epidemiologic observational evidence on the effect of periodontitis on diabetes An update of the EFP-AAP review. J Clin Periodontol. 2018;45:167–87.

273. Almeida FT, Pacheco-Pereira C, Flores-Mir C, Le LH, Jaremko JL, Major PW. Diagnostic ultrasound assessment of temporomandibular joints: A systematic review and meta-analysis. Dentomaxillofac Radiol. 2019;48.

274. Bueno CH, Pereira DD, Pattussi MP, Grossi PK, Grossi ML. Gender differences in temporomandibular disorders in adult populational studies: A systematic review and meta-analysis. J Oral Rehabil. 2018;45:720–9.

275. Troiano G, Mastrangelo F, Caponio VCA, Laino L, Cirillo N, Lo Muzio L. Predictive Prognostic Value of Tissue-Based MicroRNA Expression in Oral Squamous Cell Carcinoma: A Systematic Review and Meta-analysis. J Dent Res. 2018;97:759–66.

276. Afroz S, Naritani M, Hosoki H, Takechi K, Okayama Y, Matsuka Y. Prevalence of posterior disc displacement of the temporomandibular joint in patients with temporomandibular disorders: Systematic review and meta-analyses. J Oral Facial Pain Headache. 2018;32:277–86.

277. de Souza Melo G, Batistella EÂ, Bertazzo-Silveira E, Simek Vega Gonçalves TM, Mendes de Souza BD, Porporatti AL, et al. Association of sleep bruxism with ceramic restoration failure: A systematic review and meta-analysis. J Prosthet Dent. 2018;119:354–62.

278. Hariyani N, Setyowati D, Spencer AJ, Luzzi L, Do LG. Root caries incidence and increment in the population – A systematic review, meta-analysis and meta-regression of longitudinal studies. J Dent. 2018;77:1–7.

279. Fitzpatrick DG, Goh M, Howlett DC, Williams M. Bicycle helmets are protective against facial injuries, including facial fractures: a meta-analysis. Int J Oral Maxillofac Surg. 2018;47:1121–5.

280. Mello FW, Miguel AFP, Dutra KL, Porporatti AL, Warnakulasuriya S, Guerra ENS, et al. Prevalence of oral potentially malignant disorders: A systematic review and meta-analysis. J Oral Pathol Med. 2018;47:633–40.

281. Garot E, Denis A, Delbos Y, Manton D, Silva M, Rouas P. Are hypomineralised lesions on second primary molars (HSPM) a predictive sign of molar incisor hypomineralisation (MIH)? A systematic review and a meta-analysis. J Dent. 2018;72:8–13.

282. Zaror C, Martínez-Zapata MJ, Abarca J, Díaz J, Pardo Y, Pont À, et al. Impact of traumatic dental injuries on quality of life in preschoolers and schoolchildren: A systematic review and meta-analysis. Community Dent Oral Epidemiol. 2018;46:88–101.

283. Barbosa DAF, Barros ID, Teixeira RC, Pimenta AVM, Kurita LM, Silva PGB, et al. Imaging aspects of the mandibular incisive canal: A PROSPERO-registered systematic review and meta-analysis of cone beam computed tomography studies. Int J Oral Maxillofac Implants. 2019;34:423–33.

284. Tam S, Fu S, Xu L, Krause KJ, Lairson DR, Miao H, et al. The epidemiology of oral human papillomavirus infection in healthy populations: A systematic review and meta-analysis. Oral Oncol. 2018;82:91–9.

285. Brandão Neto JDS, Aires FT, Dedivitis RA, Matos LL, Cernea CR. Comparison between magnetic resonance and computed tomography in detecting mandibular invasion in oral cancer: A systematic review and diagnostic meta-analysis: MRI x CT in mandibular invasion. Oral Oncol. 2018;78:114–8.

286. Ziukaite L, Slot DE, Van der Weijden FA. Prevalence of diabetes mellitus in people clinically diagnosed with periodontitis: A systematic review and meta-analysis of epidemiologic studies. J Clin Periodontol. 2018;45:650–62.

287. Navarro Azevedo de Azeredo F, Silva Guimarães L, Azeredo A. Antunes L, Santos Antunes L. Global prevalence of dental caries in athletes with intellectual disabilities: An epidemiological systematic review and meta-analysis. Spec Care Dent. 2019;39:114–24.

288. Bett JVS, Batistella EÂ, Melo G, Munhoz EDA, Silva CAB, Guerra ENDS, et al. Prevalence of oral mucosal disorders during pregnancy: A systematic review and meta-analysis. J Oral Pathol Med. 2019;48:270–7.

289. Zhao D, Khawaja AT, Jin L, Li K-Y, Tonetti M, Pelekos G. The directional and non-directional associations of periodontitis with chronic kidney disease: A systematic review and meta-analysis of observational studies. J Periodontal Res. 2018;53:682–704.

290. Dourado MR, Guerra ENS, Salo T, Lambert DW, Coletta RD. Prognostic value of the immunohistochemical detection of cancer-associated fibroblasts in oral cancer: A systematic review and meta-analysis. J Oral Pathol Med. 2018;47:443–53.

291. Ferreira SD, Martins CC, Amaral SA, Vieira TR, Albuquerque BN, Cota LOM, et al. Periodontitis as a risk factor for peri-implantitis: Systematic review and meta-analysis of observational studies. J Dent. 2018;79:1–10.

292. Andrade RNM, Vieira WDA, Bernardino ÍDM, Franco A, Paranhos LR. Reliability of palatal rugoscopy for sexual dimorphism in forensic dentistry: A systematic literature review and meta-analysis. Arch Oral Biol. 2019;97:25–34.

293. Al Ayyan W, Al Halabi M, Hussein I, Khamis AH, Kowash M. A systematic review and meta-analysis of primary teeth caries studies in Gulf Cooperation Council States. Saudi Dent J. 2018;30:175–82.

294. Sun W, Xia K, Tang L, Liu C, Zou L, Liu J. Accuracy of panoramic radiography in diagnosing maxillary sinus-root relationship: A systematic review and meta-analysis. Angle Orthod. 2018;88:819–29.

295. Magno MB, Neves AB, Ferreira DM, Pithon MM, Maia LC. The relationship of previous dental trauma with new cases of dental trauma. A systematic review and meta-analysis. Dent Traumatol. 2019;35:3–14.

296. Fernandes LM, Neto JCL, Lima TFR, Magno MB, Santiago BM, Cavalcanti YW, et al. The use of mouthguards and prevalence of dento-alveolar trauma among athletes: A systematic review and meta-analysis. Dent Traumatol. 2019;35:54–72.

297. González-Álvarez L, García-Martín JM, García-Pola MJ. Association between geographic tongue and psoriasis: A systematic review and meta-analyses. J Oral Pathol Med. 2019;48:365–72.

298. Mupparapu M, Baddam VRR, Lingamaneni KP, Singer SR. Dental x-ray exposure is not associated with risk of meningioma: A 2019 meta-analysis. Quintessence Int. 2019;50:822–9.

299. Pentapati KC, Siddiq H, Yeturu SK. Global and regional estimates of the prevalence of root caries – Systematic review and meta-analysis. Saudi Dent J. 2019;31:3–15.

300. Favaro Zeola L, Soares PV, Cunha-Cruz J. Prevalence of dentin hypersensitivity: Systematic review and meta-analysis. J Dent. 2019;81:1–6.

301. Martins JNR, Marques D, Silva EJNL, Caramês J, Mata A, Versiani MA. Second root and second root canal prevalence in maxillary first and second premolars assessed by cone beam computed tomography - a systematic review and meta-analysis. Rev Port Estomatol Med Dent Cir Maxilofac. 2019;60:37–50.

302. Berrocal C, Terrero-Pérez Á, Peralta-Mamani M, Rubira-Bullen IRF, Honório HM, de Carvalho IMM, et al. Cervical vertebrae anomalies and cleft lip and palate: A systematic review and meta-analysis. Dentomaxillofac Radiol. 2019;48.

303. Gilheaney Ó, Béchet S, Kerr P, Kenny C, Smith S, Kouider R, et al. The prevalence of oral stage dysphagia in adults presenting with temporomandibular disorders: a systematic review and meta-analysis. Acta Odontol Scand. 2018;76:448–58.

304. Mergoni G, Percudani D, Lodi G, Bertani P, Manfredi M. Prevalence of Candida Species in Endodontic Infections: Systematic Review and Meta-analysis. J Endod. 2018;44:1616-1625.e9.

305. Doğramacı EJ, Rossi-Fedele G, Dreyer CW. Malocclusions in young children: Does breast-feeding really reduce the risk? A systematic review and meta-analysis. J Am Dent Assoc. 2017;148:566-574.e6.

306. Alaizari NA, Al-Maweri SA, Al-Shamiri HM, Tarakji B, Shugaa-Addin B. Hepatitis C virus infections in oral lichen planus: a systematic review and meta-analysis. Aust Dent J. 2016;61:282–7.

307. Lingen MW, Tampi MP, Urquhart O, Abt E, Agrawal N, Chaturvedi AK, et al. Adjuncts for the evaluation of potentially malignant disorders in the oral cavity: Diagnostic test accuracy systematic review and meta-analysis—a report of the American Dental Association. J Am Dent Assoc. 2017;148:797-813.e52.

308. Kadir A, Mossey PA, Blencowe H, Moorthie S, Lawn JE, Mastroiacovo P, et al. Systematic review and meta-analysis of the birth prevalence of orofacial clefts in low- and middle-income countries. Cleft Palate-Craniofac J. 2017;54:571–81.

309. Haas LF, Zimmermann GS, De Luca Canto G, Flores-Mir C, Corrêa M. Precision of cone beam CT to assess periodontal bone defects: A systematic review and meta-analysis. Dentomaxillofac Radiol. 2018;47.

310. Doğramacı EJ, Rossi-Fedele G. Establishing the association between nonnutritive sucking behavior and malocclusions: A systematic review and meta-analysis. J Am Dent Assoc. 2016;147:926-934.e6.

311. Mermod M, Tolstonog G, Simon C, Monnier Y. Extracapsular spread in head and neck squamous cell carcinoma: A systematic review and meta-analysis. Oral Oncol. 2016;62:60–71.

312. Barbosa KGN, de Macedo Bernardino Í, d’Avila S, Ferreira EF, Ferreira RC. Systematic review and meta-analysis to determine the proportion of maxillofacial trauma resulting from different etiologies among children and adolescents. Oral Maxillofac Surg. 2017;21:131–45.

313. De Lima CL, Acevedo AC, Grisi DC, Taba M, Guerra E, De Luca Canto G. Host-derived salivary biomarkers in diagnosing periodontal disease: Systematic review and meta-analysis. J Clin Periodontol. 2016;43:492–502.

314. Javed F, Feng C, Kopycka-Kedzierawski DT. Incidence of early childhood caries: A systematic review and meta-analysis. J Investig Clin Dent. 2017;8.

315. Blanco R, Colombo A, Pardo R, Suazo J. Maternal biomarkers of methylation status and non-syndromic orofacial cleft risk: a meta-analysis. Int J Oral Maxillofac Surg. 2016;45:1323–32.

316. Ma RH, Ge ZP, Li G. Detection accuracy of root fractures in cone-beam computed tomography images: A systematic review and meta-analysis. Int Endod J. 2016;49:646–54.

317. Pentapati KC, Yeturu SK, Siddiq H. Systematic review and meta‑analysis of the prevalence of molar‑incisor hypomineralization. J Int Oral Health. 2017;9:243–50.

318. Sedghizadeh PP, Billington WD, Paxton D, Ebeed R, Mahabady S, Clark GT, et al. Is p16-positive oropharyngeal squamous cell carcinoma associated with favorable prognosis? A systematic review and meta-analysis. Oral Oncol. 2016;54:15–27.

319. Casett E, Réus JC, Stuginski-Barbosa J, Porporatti AL, Carra MC, Peres MA, et al. Validity of different tools to assess sleep bruxism: a meta-analysis. J Oral Rehabil. 2017;44:722–34.

320. Palaska PK, Antonarakis GS. Prevalence and patterns of permanent tooth agenesis in individuals with Down syndrome: a meta-analysis. Eur J Oral Sci. 2016;124:317–28.

321. Alayyan W, Halabi MA, Hussein I, Khamis A, Kowash M. A systematic review and meta-Analysis of school children’s caries studies in gulf cooperation council states. J Intl Soc Prev Community Dent. 2017;7:234–41.

322. Talwar S, Utneja S, Nawal RR, Kaushik A, Srivastava D, Oberoy SS. Role of Cone-beam Computed Tomography in Diagnosis of Vertical Root Fractures: A Systematic Review and Meta-analysis. J Endod. 2016;42:12–24.

323. Ferreira JB, Christovam IO, Alencar DS, DaMotta AFJ, Mattos CT, Cury-Saramago A. Accuracy and reproducibility of dental measurements on tomographic digital models: A systematic review and meta-analysis. Dentomaxillofac Radiol. 2017;46.

324. Yi J, Sun Y, Li Y, Li C, Li X, Zhao Z. Cone-beam computed tomography versus periapical radiograph for diagnosing external root resorption: A systematic review and meta-analysis. Angle Orthod. 2017;87:328–37.

325. Persoon IF, Crielaard W, Özok AR. Prevalence and nature of fungi in root canal infections: a systematic review and meta-analysis. Int Endod J. 2017;50:1055–66.

326. Pupo YM, Pantoja LLQ, Veiga FF, Stechman-Neto J, Zwir LF, Farago PV, et al. Diagnostic validity of clinical protocols to assess temporomandibular disk displacement disorders: a meta-analysis. Oral Surg Oral Med Oral Pathol Oral Radiol. 2016;122:572–86.

327. da Silva SN, Gimenez T, Souza RC, Mello-Moura ACV, Raggio DP, Morimoto S, et al. Oral health status of children and young adults with autism spectrum disorders: systematic review and meta-analysis. Int J Paediatr Dent. 2017;27:388–98.

328. Winand C, Shetty A, Senior A, Ganatra S, De Luca Canto G, Alsufyani N, et al. Digital imaging capability for caries detection: A meta-analysis. JDR Clin Transl Res. 2016;1:112–21.

329. Salineiro FCS, Kobayashi-Velasco S, Braga MM, Cavalcanti MGP. Radiographicdiagnosis of root fractures: A systematic review, meta-analyses and sources of heterogeneity. Dentomaxillofac Radiol. 2017;46.

330. Antonarakis GS, Palaska PK, Suri S. Permanent tooth agenesis in individuals with non-syndromic Robin sequence: a systematic review and meta-analysis. Orthodont Craniofac Res. 2017;20:216–26.

331. Mushi MF, Bader O, Taverne-Ghadwal L, Bii C, Groß U, Mshana SE. Oral candidiasis among African human immunodeficiency virus-infected individuals: 10 years of systematic review and meta-analysis from sub-Saharan Africa. J Oral Microbiol. 2017;9.

332. Joury E, Bernabe E, Sabbah W, Nakhleh K, Gurusamy K. Systematic review and meta-analysis of randomised controlled trials on the effectiveness of school-based dental screening versus no screening on improving oral health in children. J Dent. 2017;58:1–10.

333. Mozaffari HR, Ramezani M, Mahmoudiahmadabadi M, Omidpanah N, Sadeghi M. Salivary and serum levels of tumor necrosis factor-alpha in oral lichen planus: a systematic review and meta-analysis study. Oral Surg Oral Med Oral Pathol Oral Radiol. 2017;124:e183–9.

334. Rakhshan V, Rakhshan A. Systematic review and meta-analysis of congenitally missing permanent dentition: Sex dimorphism, occurrence patterns, associated factors and biasing factors. Int Orthod. 2016;14:273–94.

335. Worth V, Perry R, Ireland T, Wills AK, Sandy J, Ness A. Are people with an orofacial cleft at a higher risk of dental caries? A systematic review and meta-analysis. Brit Dent J. 2017;223:37–47.

336. Da Silva CG, Pachêco-Pereira C, Porporatti AL, Savi MG, Peres MA, Flores-Mir C, et al. Prevalence of clinical signs of intra-articular temporomandibular disorders in children and adolescents A systematic review and meta-analysis. J Am Dent Assoc. 2016;147:10-18.e8.

337. Kumar S, Tadakamadla J, Johnson NW. Effect of toothbrushing frequency on incidence and increment of dental caries: A systematic review and meta-analysis. J Dent Res. 2016;95:1230–6.

338. Abbood HM, Hinz J, Cherukara G, Macfarlane TV. Validity of self-reported Periodontal disease: A systematic review and meta-analysis. J Periodontol. 2016;87:1474–83.

339. Eslamipour F, Afshari Z, Najimi A. Prevalence of orthodontic treatment need in permanent dentition of Iranian population: A systematic review and meta analysis of observational studies. Dent Res J. 2018;15:1–10.

340. Pękala PA, Henry BM, Pękala JR, Frączek PA, Taterra D, Natsis K, et al. The pterygoalar bar: A meta-analysis of its prevalence, morphology and morphometry. J Cranio-Maxillofac Surg. 2017;45:1535–41.

341. Leonardi Dutra K, Haas L, Porporatti AL, Flores-Mir C, Nascimento Santos J, Mezzomo LA, et al. Diagnostic accuracy of cone-beam computed tomography and conventional radiography on apical periodontitis: A systematic review and meta-analysis. J Endod. 2016;42:356–64.

342. Porto De Toledo I, Stefani FM, Porporatti AL, Mezzomo LA, Peres MA, Flores-Mir C, et al. Prevalence of otologic signs and symptoms in adult patients with temporomandibular disorders: a systematic review and meta-analysis. Clin Oral Invest. 2017;21:597–605.

343. Nieri M, Tofani E, Defraia E, Giuntini V, Franchi L. Enamel defects and aphthous stomatitis in celiac and healthy subjects: Systematic review and meta-analysis of controlled studies. J Dent. 2017;65:1–10.

344. Aghbari SMH, Abushouk AI, Attia A, Elmaraezy A, Menshawy A, Ahmed MS, et al. Malignant transformation of oral lichen planus and oral lichenoid lesions: A meta-analysis of 20095 patient data. Oral Oncol. 2017;68:92–102.

345. Akbari M, Lankarani KB, Honarvar B, Tabrizi R, Mirhadi H, Moosazadeh M. Prevalence of malocclusion among Iranian children: A systematic review and meta-Analysis. Dent Res J. 2016;13:387–95.

346. Long H, Zhou Y, Ye N, Liao L, Jian F, Wang Y, et al. Diagnostic accuracy of CBCT for tooth fractures: A meta-analysis. J Dent. 2014;42:240–8.

347. Khan SQ. Dental caries in arab league countries: A systematic review and meta-analysis. Int Dent J. 2014;64:173–80.

348. Tomás I, Diz P, Tobías A, Scully C, Donos N. Periodontal health status and bacteraemia from daily oral activities: Systematic review/meta-analysis. J Clin Periodontol. 2012;39:213–28.

349. Lodi G, Pellicano R, Carrozzo M. Hepatitis C virus infection and lichen planus: A systematic review with meta-analysis. Oral Dis. 2010;16:601–12.

350. Fidalgo TKDS, Freitas-Fernandes LB, Ammari M, Mattos CT, De Souza IPR, Maia LC. The relationship between unspecific s-IgA and dental caries: A systematic review and meta-analysis. J Dent. 2014;42:1372–81.

351. Delli K, Dijkstra PU, Stel AJ, Bootsma H, Vissink A, Spijkervet FKL. Diagnostic properties of ultrasound of major salivary glands in Sjögren’s syndrome: A meta-analysis. Oral Dis. 2015;21:792–800.

352. Ameer Al-Jundi M, John MT, Setz JM, Szentpétery A, Kuss O. Meta-analysis of treatment need for temporomandibular disorders in adult nonpatients. J Orofac Pain. 2008;22:97–107.

353. Ye X, Zhang J, Tan Y, Chen G, Zhou G. Meta-analysis of two computer-assisted screening methods for diagnosing oral precancer and cancer. Oral Oncol. 2015;51:966–75.

354. Dong XY, He S, Zhu L, Dong TY, Pan SS, Tang LJ, et al. The diagnostic value of high-resolution ultrasonography for the detection of anterior disc displacement of the temporomandibular joint: A meta-analysis employing the HSROC statistical model. Int J Oral Maxillofac Surg. 2015;44:852–8.

355. Gimenez T, Piovesan C, Braga MM, Raggio DP, Deery C, Ricketts DN, et al. Visual inspection for caries detection: A systematic review and meta-analysis. J Dent Res. 2015;94:895–904.

356. Pommer B, Ulm C, Lorenzoni M, Palmer R, Watzek G, Zechner W. Prevalence, location and morphology of maxillary sinus septa: Systematic review and meta-analysis. J Clin Periodontol. 2012;39:769–73.

357. Saridin CP, Raijmakers PGHM, Tuinzing DB, Becking AG. Bone scintigraphy as a diagnostic method in unilateral hyperactivity of the mandibular condyles: A review and meta-analysis of the literature. Int J Oral Maxillofac Surg. 2011;40:11–7.

358. Herkrath APCDQ, Herkrath FJ, Rebelo MAB, Vettore MV. Parental age as a risk factor for non-syndromic oral clefts: A meta-analysis. J Dent. 2012;40:3–14.

359. Nascimento GG, Leite FRM, Do LG, Peres KG, Correa MB, Demarco FF, et al. Is weight gain associated with the incidence of periodontitis? A systematic review and meta-analysis. J Clin Periodontol. 2015;42:495–505.

360. Don KR, Ramani P, Ramshankar V, Sherlin HJ, Premkumar P, Natesan A. Promoter hypermethylation patterns of P16, DAPK and MGMT in Oral Squamous Cell Carcinoma: A systematic review and meta-analysis. Indian J Dent Res. 2014;25:797–805.

361. Varela-Centelles P, Loira-Gago M, Seoane-Romero JM, Takkouche B, Monteiro L, Seoane J. Detection of the posterior superior alveolar artery in the lateral sinus wall using computed tomography/cone beam computed tomography: A prevalence meta-analysis study and systematic review. Int J Oral Maxillofac Surg. 2015;44:1405–10.

362. Schwendicke F, Dörfer CE, Schlattmann P, Page LF, Thomson WM, Paris S. Socioeconomic inequality and caries: A systematic review and meta-analysis. J Dent Res. 2015;94:10–8.

363. Li C, Sheng S, Men Y, Sun H, Xia H, Li L. Emission computed tomography for the diagnosis of mandibular invasion by head and neck cancers: A systematic review and meta-analysis. J Oral Maxillofac Surg. 2015;73:1875.e1-1875.e11.

364. Chaffee BW, Weston SJ. Association between chronic periodontal disease and obesity: A systematic review and meta-analysis. J Periodontol. 2010;81:1708–24.

365. Schwendicke F, Tzschoppe M, Paris S. Radiographic caries detection: A systematic review and meta-analysis. J Dent. 2015;43:924–33.

366. Dasanayake AP, Silverman AJ, Warnakulasuriya S. Maté drinking and oral and oro-pharyngeal cancer: A systematic review and meta-analysis. Oral Oncol. 2010;46:82–6.

367. Gong Y, Wei B, Yu L, Pan W. Type 2 diabetes mellitus and risk of oral cancer and precancerous lesions: A meta-analysis of observational studies. Oral Oncol. 2015;51:332–40.

368. Anthonappa RP, King NM, Rabie ABM. Diagnostic tools used to predict the prevalence of supernumerary teeth: A meta-analysis. Dentomaxillofac Radiol. 2012;41:444–9.

369. Li L, Gu H, Zhang G. Association between recurrent aphthous stomatitis and Helicobacter pylori infection: A meta-analysis. Clin Oral Invest. 2014;18:1553–60.

370. Govers TM, Hannink G, Merkx MAW, Takes RP, Rovers MM. Sentinel node biopsy for squamous cell carcinoma of the oral cavity and oropharynx: A diagnostic meta-analysis. Oral Oncol. 2013;49:726–32.

371. Kassebaum NJ, Bernabé E, Dahiya M, Bhandari B, Murray CJL, Marcenes W. Global Burden of Severe Tooth Loss: A Systematic Review and Meta-analysis. J Dent Res. 2014;93:20S-28S.

372. Antonarakis GS, Palaska P-K, Herzog G. Caries prevalence in non-syndromic patients with cleft lip and/or palate: A meta-analysis. Caries Res. 2013;47:406–13.

373. Aldrigui JM, Jabbar NS, Bonecker M, Braga MM, Wanderley MT. Trends and associated factors in prevalence of dental trauma in Latin America and Caribbean: A systematic review and meta-analysis. Community Dent Oral Epidemiol. 2014;42:30–42.

374. Li C, Su N, Yang X, Yang X, Shi Z, Li L. Ultrasonography for detection of disc displacement of temporomandibular joint: A systematic review and meta-analysis. J Oral Maxillofac Surg. 2012;70:1300–9.

375. Liao G, Wang Y, Zhou Y-Q, Li T-W, Zeng D-Q, Zeng X, et al. Host genetic susceptibility to oral cancer: Evidence from meta-analyses and pooled analyses. Oral Dis. 2014;20:644–9.

376. O’Rorke MA, Ellison MV, Murray LJ, Moran M, James J, Anderson LA. Human papillomavirus related head and neck cancer survival: A systematic review and meta-analysis. Oral Oncol. 2012;48:1191–201.

377. Gómez I, Seoane J, Varela-Centelles P, Diz P, Takkouche B. Is diagnostic delay related to advanced-stage oral cancer? A meta-analysis. Eur J Oral Sci. 2009;117:541–6.

378. Corbella S, Taschieri S, Francetti L, de Siena F, Del Fabbro M. Periodontal disease as a risk factor for adverse pregnancy outcomes: A systematic review and meta-analysis of case-control studies. Odontology. 2012;100:232–40.

379. Guerra ENS, Acevedo AC, Leite AF, Gozal D, Chardin H, De Luca Canto G. Diagnostic capability of salivary biomarkers in the assessment of head and neck cancer: A systematic review and meta-analysis. Oral Oncol. 2015;51:805–18.

380. Haas LF, Dutra K, Porporatti AL, Mezzomo LA, De Luca Canto G, Flores-Mir C, et al. Anatomical variations of mandibular canal detected by panoramic radiography and CT: A systematic review and meta-analysis. Dentomaxillofac Radiol. 2015;45.

381. Perinetti G, Westphalen GH, Biasotto M, Salgarello S, Contardo L. The diagnostic performance of dental maturity for identification of the circumpubertal growth phases: A meta-analysis. Prog Orthod. 2013;14:1–13.

382. Bie M, Wu P, Zhou J, Li Y, Zhao L. Periodontal health status in cirrhotic patients: a systematic review and meta-analysis. BMC ORAL HEALTH. 2023;23.

383. Ganesan S, Peter T, Withanage M, Boksa F, Zeng E, Martinez A, et al. COVID-19 associated oral and oropharyngeal microbiome: Systematic review and meta-analysis. PERIODONTOLOGY 2000. 2023. https://doi.org/10.1111/prd.12489.

384. AlMoharib H, AlRowis R, AlMubarak A, Almadhoon H, Ashri N. The relationship between matrix metalloproteinases-8 and peri-implantitis: A systematic review and meta-analysis. SAUDI DENTAL JOURNAL. 2023;35:283–93.

385. Kania M, Malinowski K, Slowik J, Wladysiuk M, Szopa M. Inflammatory markers in pregnant women with periodontal diseases-Systematic review and network meta-analysis. ORAL SCIENCE INTERNATIONAL. 2023;20:67–77.

386. Vieira W, Paranhos L, Meneses-Santos D, Floriano L, Borges G, Sponchiado E, et al. Association between the prevalence of pulp stones and calcified atherosclerotic plaques: a systematic review and meta-analysis. DENTOMAXILLOFACIAL RADIOLOGY. 2023;52.

387. Tran D, Vu C, Phan Q, Nguyen C. Prevalence of periodontal disease among Vietnamese adults: A systematic review and meta-analysis. DENTAL AND MEDICAL PROBLEMS. 2023;60:145–52.

388. Yang Y, Tian Y, Sun L, Qu H, Chen F. Relationship between Presence of Third Molars and Prevalence of Periodontal Pathology of Adjacent Second Molars: a Systematic Review and Meta-analysis. CHINESE JOURNAL OF DENTAL RESEARCH. 2022;25:45–55.

389. Ferrari-Piloni C, Barros L, Evangelista K, Serra-Negra J, Silva M, Valladares-Neto J. Prevalence of Bruxism in Brazilian Children: A Systematic Review and Meta-Analysis. PEDIATRIC DENTISTRY. 2022;44:8-+.

390. Pourzare-Mehrbani S, Motahari P, Pournagi-Azar F, Alizadeh P, Salehnia F. Association of helicobacter pylori and oral lichen planus: A systematic review and meta-analysis. JOURNAL OF ORAL HEALTH AND ORAL EPIDEMIOLOGY. 2021;10:1–6.

391. Pasnik-Chwalik B, Konopka T. Impact of periodontitis on the Oral Health Impact Profile: A systematic review and meta-analysis. DENTAL AND MEDICAL PROBLEMS. 2020;57:423–31.

392. Motahari P, Pournaghi-Azar F, Khodadadi P. Role of tumor necrosis factor-alpha in pathogenesis of recurrent aphthous stomatitis: A systematic review and meta-analysis. JOURNAL OF ORAL HEALTH AND ORAL EPIDEMIOLOGY. 2020;9:108–15.

393. Martorano-Fernandes L, Dornelas-Figueira L, Marcello-Machado R, Silva R, Magno M, Maia L, et al. Oral candidiasis and denture stomatitis in diabetic patients: Systematic review and meta-analysis. BRAZILIAN ORAL RESEARCH. 2020;34.

394. Gu Z, Zhang S, Zhang R, Tang H, Sun X, Liu X, et al. Prevalence of Caries in Mainland China: Evidence from 1980 to 2018: A Systematic Review and Meta-Analysis. CHINESE JOURNAL OF DENTAL RESEARCH. 2019;22:251–63.

395. Rabiei M, Rad H, Rad E, Ashourizadeh S. Dental status of the Iranian elderly: A systematic review and meta-analysis. JOURNAL OF INVESTIGATIVE AND CLINICAL DENTISTRY. 2019;10.

396. Kirthiga M, Murugan M, Saikia A, Kirubakaran R. Risk Factors for Early Childhood Caries: A Systematic Review and Meta-Analysis of Case Control and Cohort Studies. PEDIATRIC DENTISTRY. 2019;41:95-+.

397. Xiao J, Huang X, Alkhers N, Alzamil H, Alzoubi S, Wu T, et al. Candida albicans and Early Childhood Caries: A Systematic Review and Meta-Analysis. CARIES RESEARCH. 2018;52:102–12.

398. Carter K, Worthington S. Predictors of Third Molar Impaction: A Systematic Review and Meta-analysis. JOURNAL OF DENTAL RESEARCH. 2016;95:267–76.

399. Papadopoulos M, Chatzoudi M, Karagiannis V. Assessment of characteristic features and dental anomalies accompanying tooth transposition: A meta-analysis. AMERICAN JOURNAL OF ORTHODONTICS AND DENTOFACIAL ORTHOPEDICS. 2009;136.

*List of excluded studies after Title/Abstract assessment with reasons for exclusion*

1. Pertek Hatipoğlu F, Mağat G, Hatipoğlu Ö, Taha N, Alfirjani S, Abidin IZ, et al. Assessment of the Prevalence of Middle Mesial Canal in Mandibular First Molar: A Multinational Cross-sectional Study with Meta-analysis. J Endod. 2023;49:549–58.

Reason: Other Study Design

2. Higginson JA, Breik O, Thompson AH, Ashrafian H, Hardman JC, Takats Z, et al. Diagnostic accuracy of intraoperative margin assessment techniques in surgery for head and neck squamous cell carcinoma: A meta-analysis. Oral Oncol. 2023;142.

Reason: Not related to topic

3. Atieh MA, Shah M, Ameen M, Tawse-Smith A, Alsabeeha NHM. Influence of implant restorative emergence angle and contour on peri-implant marginal bone loss: A systematic review and meta-analysis. Clin Implant Dent Relat Res. 2023. https://doi.org/10.1111/cid.13214.

Reason: Intervention

4. da Silva Correia AG, Alves JN, da Mota Santos SA, Guerra DR, Garção DC. Anatomical variations in the relationship between the spinal accessory nerve and internal jugular vein: a systematic review and meta-analysis. Int J Oral Maxillofac Surg. 2023;52:13–8.

Reason: Not related to topic

5. Neto RMS, Zotarelli-Filho IJ, Ribeiro da Silva CEXS. Meta-analysis of the Major Clinical Results of the Treatment with 1-Point Fixation in Fractures in the Zygomatic-Maxillary Complex: Success Rate and Complications. J Maxillofac Oral Surg. 2023;22:1–8.

Reason: Intervention

6. Rodríguez-Fuentes ME, Pérez-Sayáns M, Carreras-Presas CM, Marichalar-Mendia X, Bagán-Debón L, López-López R. Prevalence of acute oral mucosal damage secondary to the use of systemic antineoplastics: A systematic review and meta-analysis. Oral Surg Oral Med Oral Pathol Oral Radiol. 2023;135:385–95.

Reason: Intervention

7. Kapoor E, Mantilla-Rivas E, Rana MS, Aivaz M, Duarte-Bateman D, Escandón JM, et al. Facial Nerve Dysfunction After Mandibular Distraction Osteogenesis in Patients with Robin Sequence: A Systematic Review and Meta-Analysis. Cleft Palate-Craniofac J. 2023;60:395–404.

Reason: Intervention

8. Xue S, Song G, Zhu Y, Zhang N, Tan Y. The efficacy and safety of VEGF/VEGFR inhibitors in patients with recurrent or metastatic nasopharyngeal carcinoma: A meta-analysis. Oral Oncol. 2022;135.

Reason: Not related to topic

9. Yao Y, Luo A, Hao Y. Selective versus stepwise removal of deep carious lesions: A meta-analysis of randomized controlled trials. J Dent Sci. 2023;18:17–26.

Reason: Intervention and other study design

10. Giannakoulas DG, Koletsi D, Tzanetakis GN. Assessment of spin in abstracts of Endodontic Systematic Reviews with meta-analyses published between 2010 and 2022. Are we in need of more transparent interpretation of findings? Int Endod J. 2022;55:1347–58.

Reason: Other study design

11. Seehra J, Mortaja K, Wazwaz F, Papageorgiou SN, Newton JT, Cobourne MT. Interventions to facilitate the successful eruption of impacted maxillary incisor teeth due to the presence of a supernumerary: A systematic review and meta-analysis. Am J Orthod Dentofacial Orthop. 2023;163:594–608.

Reason: Intervention

12. Mahardawi B, Jiaranuchart S, Tompkins KA, Pimkhaokham A. Efficacy of the autogenous dentin graft for implant placement: a systematic review and meta-analysis of randomized controlled trials. Int J Oral Maxillofac Surg. 2023;52:604–12.

Reason: Intervention

13. Batista NVR, Valdez RMA, Silva EMVDM, Melo TS, Pereira JRD, Warnakulasuriya S, et al. Association between autoimmune rheumatic diseases and head and neck cancer: Systematic review and meta-analysis. J Oral Pathol Med. 2023;52:357–64.

Reason: Not related to topic

14. Scardini IL, Sarra G, Braga MM, Dos Santos M, Freire LG. The Effect of Number of Visits, Use of Solvent and Gutta-percha Removal Technique on Postoperative Pain following Nonsurgical Endodontic Retreatment; A Systematic Review and Meta-analysis. Iran Endod J. 2023;18:71–84.

Reason: Intervention

15. Theodoridou M-Z, Zarkadi A-E, Zymperdikas VF, Papadopoulos MA. Long-term effectiveness of non-surgical open-bite treatment: a systematic review and meta-analysis. Prog Orthod. 2023;24.

Reason: Intervention

16. Molina A, Huck O, Herrera D, Montero E. The association between respiratory diseases and periodontitis: A systematic review and meta-analysis. J Clin Periodontol. 2023;50:842–87.

Reason: Other study design

17. Romandini M, Ruales-Carrera E, Sadilina S, Hämmerle CHF, Sanz M. Minimal invasiveness at dental implant placement: A systematic review with meta-analyses on flapless fully guided surgery. Periodontol 2000. 2023;91:89–112.

Reason: Intervention

18. Kang Z, Jin T, Li X, Wang Y, Xu T, Wang Y, et al. Progression and postoperative complications of osteoradionecrosis of the jaw: a 20-year retrospective study of 124 non-nasopharyngeal cancer cases and meta-analysis. BMC Oral Health. 2022;22.

Reason: Intervention

19. Wortmann DE, van Minnen B, Delli K, Schortinghuis J, Raghoebar GM, Vissink A. Harvesting anterior iliac crest or calvarial bone grafts to augment severely resorbed edentulous jaws: a systematic review and meta-analysis of patient-reported outcomes. Int J Oral Maxillofac Surg. 2023;52:481–94.

Reason: Intervention

20. Ntolou P, Pani P, Panis V, Madianos P, Vassilopoulos S. The effect of antiretroviral therapyon the periodontal conditions of patients with HIV infection: A systematic review and meta-analysis. J Clin Periodontol. 2023;50:170–82.

Reason: Intervention

21. Martins CC, Lockhart PB, Firmino RT, Kilmartin C, Cahill TJ, Dayer M, et al. Bacteremia following different oral procedures: Systematic review and meta-analysis. Oral Dis. 2023. https://doi.org/10.1111/odi.14531.

Reason: Intervention

22. Rocha RS, Vianna CP, Trojan LC, Padovan LEM, dos Santos MCGL. Comparison of sinusitis rate after sinus lift procedure and zygomatic implant surgery: a meta-analysis. Oral Maxillofac Surg. 2023. https://doi.org/10.1007/s10006-023-01159-1.

Reason: Intervention

23. Bezerra PL, de Carvalho Júnior AD, da Silva AF, Pereira NEG, da Costa SRR, de Sousa JNL, et al. Effects of periodontal treatment on the C-reactive protein levels in hemodialysis patients: A systematic review and meta-analysis. Spec Care Dent. 2023. https://doi.org/10.1111/scd.12834.

Reason: Intervention

24. Mordini L, Patianna GP, Di Domenico GL, Natto ZS, Valente NA. The use of corticosteroids in the lateral sinus augmentation surgical procedure: A systematic review and meta-analysis. Clin Implant Dent Relat Res. 2022;24:776–91.

Reason: Intervention

25. Dawoud BES, Kent S, Tabbenor O, Markose G, Java K, Kyzas P. Does anticoagulation improve outcomes of microvascular free flap reconstruction following head and neck surgery: a systematic review and meta-analysis. Br J Oral Maxillofac Surg. 2022;60:1292–302.

Reason: Not related to topic and Intervention

26. Darriba I, Seidel A, Moreno F, Botelho J, Machado V, Mendes JJ, et al. Influence of low insertion torque values on survival rate of immediately loaded dental implants: A systematic review and meta-analysis. J Clin Periodontol. 2023;50:158–69.

Reason: Intervention

27. Sales PHDH, Cetira Filho EL, Silva PGDB, Costa FWG, Leão JC. Effectiveness of Autogenous Chin Bone Graft in Reconstructive Surgery of Cleft Patients: A Systematic Review With Meta-Analysis and Algorithm of Treatment. J Oral Maxillofac Surg. 2023. https://doi.org/10.1016/j.joms.2023.04.011.

Reason: Intervention

28. AlMoharib HS, AlRowis R, AlMubarak A, Waleed Almadhoon H, Ashri N. The relationship between matrix metalloproteinases-8 and peri-implantitis: A systematic review and meta-analysis. Saudi Dent J. 2023;35:283–93.

Reason: Intervention

29. Boscolo-Rizzo P, Tirelli G, Polesel J, Sia E, Phillips V, Borsetto D, et al. TERT promoter mutations in head and neck squamous cell carcinoma: A systematic review and meta-analysis on prevalence and prognostic significance. Oral Oncol. 2023;140.

Reason: Not related to topic

30. He S, Choong EKM, Duangthip D, Chu CH, Lo ECM. Clinical interventions with various agents to prevent early childhood caries: A systematic review with network meta-analysis. Int J Paediatr Dent. 2023. https://doi.org/10.1111/ipd.13055.

Reason: Intervention

31. Sozkes S, Sozkes S. Use of toothbrushing in conjunction with chlorhexidine for preventing ventilator-associated pneumonia: A random-effect meta-analysis of randomized controlled trials. Int J Dent Hyg. 2023;21:389–97.

Reason: Intervention

32. Salgado-Peralvo AO, Montero-Alonso M, Kewalramani N, Pérez-Sayáns-García M, Mateos-Moreno MV, Garcillán-Izquierdo MR. Prevalence of aphthous stomatitis in patients with inflammatory bowel disease after the treatment with monoclonal antibodies: A systematic review and meta-analysis. Med Oral Patol Oral Cir Bucal. 2022;27:e588–99.

Reason: Intervention

33. Kaul P, Malhotra M, Arora V, Agarwal N, Singh MP, Garg PK. Prognostic significance of soft tissue deposits in head and neck squamous cell carcinoma: a systematic review and meta-analysis. Int J Oral Maxillofac Surg. 2023. https://doi.org/10.1016/j.ijom.2023.01.014.

Reason: Not related to topic

34. Alghauli MA, Alqutaibi AY, Wille S, Kern M. Clinical reliability of self-adhesive luting resins compared to other adhesive procedures: A systematic review and meta-analysis. J Dent. 2023;129.

Reason: Intervention

35. Peres Lima FGG, Rios LGC, Bianchi J, Gonçalves JR, Paranhos LR, Vieira WA, et al. Complications of total temporomandibular joint replacement: a systematic review and meta-analysis. Int J Oral Maxillofac Surg. 2023;52:584–94.

Reason: Intervention

36. WANG Q, WU YUQI, Zhang YOU, ZHANG Z, XU HAO, JIANG Y, et al. EVALUATING THE OUTCOMES OF MINIMALLY INVASIVE THERAPY VS SURGERY FOR ORAL MUCOCELES: A SYSTEMATIC REVIEW AND META-ANALYSIS. J Evid-Based Dent Pract. 2023;23.

Reason: Intervention

37. Watanabe T, Sieg M, Lunde SJ, Persson M, Taneja P, Baad-Hansen L, et al. Nocebo response in dentistry: A systematic review and meta-analysis of adverse events in analgesic trials of third molar removal. J Oral Rehabil. 2023;50:332–42.

Reason: Intervention

38. Niezen ET, van Minnen B, Bos RRM, Dijkstra PU. Temporomandibular joint prosthesis as treatment option for mandibular condyle fractures: a systematic review and meta-analysis. Int J Oral Maxillofac Surg. 2023;52:88–97.

Reason: Intervention

39. Li X, Liu Y, Li C, Wang J. Sedative and adverse effect comparison between oral midazolam and nitrous oxide inhalation in tooth extraction: a meta-analysis. BMC Oral Health. 2023;23.

Reason: Intervention

40. Zhang C, Shen G, Li H, Xin Y, Shi M, Zheng Y, et al. Incidence rate of osteonecrosis of jaw after cancer treated with bisphosphonates and denosumab: A systematic review and meta‑analysis. Spec Care Dent. 2023. https://doi.org/10.1111/scd.12877.

Reason: Intervention

41. Goonetilleke SB, Lister G, Sayed O. Effect of Herbst Treatment on Mandibular Length (Co-Gn) in Patients with Class II Malocclusion: A Systematic Review and Meta-analysis. World J Dent. 2023;14:176–81.

Reason: Intervention

42. de Castro M, Machado B, Barbosa M, Soares L, Sperandio F, de Carli M, et al. Impact of the COVID-19 pandemic on head and neck cancer patients: systematic review and meta-analysis. Quintessence Int. 2023;54:320–7.

Reason: Not related to topic

43. Al-Manei KK, Alzaidi S, Almalki G, Al-Manei K, Almotairy N. Incidence and influential factors in pulp necrosis and periapical pathosis following indirect restorations: a systematic review and meta-analysis. BMC Oral Health. 2023;23.

Reason: Intervention

44. de Lucena Alves CP, Vetromilla BM, Moreno LB, Helal L, Sarkis-Onofre R, Pereira-Cenci T. Systematic reviews on the success of dental implants present low spin of information but may be better reported and interpreted: An overview of systematic reviews with meta-analysis. Clin Implant Dent Relat Res. 2022;24:105–15.

Reason: Other study design

45. Ahmed A, Wu E, Sarai R, Williams R, Breeze J. Potentially modifiable patient factors in mandible fracture complications: a systematic review and meta-analysis. Br J Oral Maxillofac Surg. 2022;60:266–70.

Reason: Intervention

46. Ali NT, El-Boghdadi RM, Ibrahim AM, Amin SAW. Clinical and microbiological effects of ultrasonically activated irrigation versus syringe irrigation during endodontic treatment: a systematic review and meta-analysis of randomized clinical trials. Odontology. 2022;110:419–33.

Reason: Intervention

47. Zhou L, Su Y, Wang J, Wang X, Liu Q, Wang J. Effect of ExposureRates With Customized Versus Conventional TitaniumMeshon Guided Bone Regeneration: Systematic Reviewand Meta-Analysis. J Oral Implantol. 2022;48:339–46.

Reason: Intervention

48. Ge KX, Quock R, Chu C-H, Yu OY. The preventive effect of glass ionomer restorations on new caries formation: A systematic review and meta-analysis. J Dent. 2022;125.

Reason: Intervention

49. Eini E, Yousefimanesh H, Ashtiani AH, Saki-Malehi A, Olapour A, Rahim F. Comparing success of immediate versus delay loading of implants in fresh sockets: a systematic review and meta-analysis. Oral Maxillofac Surg. 2022;26:185–94.

Reason: Intervention

50. Sensever FDA, de Lucena Alves CP, Lima GDS, Loomans B, Opdam N, Pereira-Cenci T. Spin and reporting in systematic reviews with meta-analysis of randomized clinical trials in restorative dentistry. J Dent. 2022;125.

Reason: Other study design

51. Abd-ElHady MS, Abd-ElAziz OM, Hakam MM, Radi IA-E. POST-SURGICAL NEUROSENSORY DYSFUNCTION OF INFERIOR ALVEOLAR NERVE IN BILATERAL SAGITTAL SPILT OSTEOTOMY OF THE MANDIBLE USING SAW VERSUS PIEZOTOME: A SYSTEMATIC REVIEW AND META-ANALYSIS. J Evid-Based Dent Pract. 2022;22.

Reason: Intervention

52. MCKENNA GJ, GJENGEDAL H, HARKIN J, HOLLAND N, MOORE C, SRINIVASAN M. EFFECT OF AUTOGENOUS BONE GRAFT SITE ON DENTAL IMPLANT SURVIVAL AND DONOR SITE COMPLICATIONS: A SYSTEMATIC REVIEW AND META-ANALYSIS. J Evid-Based Dent Pract. 2022;22.

Reason: Intervention

53. Selvaraj A, Jain RK, Nagi R, Balasubramaniam A. Correlation between gray values of cone-beam computed tomograms and Hounsfield units of computed tomograms: A systematic review and meta-analysis. Imaging Sci Dent. 2022;52:133–40.

Reason: Not related to topic

54. Singh AK, Dahal S, Singh S, Krishna KC, Chaulagain R. Is manual reduction adequate for intraoperative control of occlusion during fixation of mandibular fractures? A systematic review and meta-analysis. Br J Oral Maxillofac Surg. 2022;60:271–8.

Reason: Intervention

55. Singh AK, Khanal N, Chaulagain R, Bhujel N, Singh RP. How effective is 5-Fluorouracil as an adjuvant in the management of odontogenic keratocyst? A systematic review and meta-analysis. Br J Oral Maxillofac Surg. 2022;60:746–54.

Reason: Intervention

56. Xiao Y, Yuan P, Sun Y, Xu Y, Deng X, Wang X, et al. Comparison of topical antifungal agents for oral candidiasis treatment: a systematic review and meta-analysis. Oral Surg Oral Med Oral Pathol Oral Radiol. 2022;133:282–91.

Reason: Intervention

57. Eren SK, Uzunoğlu-Özyürek E, Karahan S. Do resin-based root canal sealers cause more postoperative pain than other sealers? A systematic review of clinical studies and meta-analysis. Quintessence Int. 2022;53:472–83.

Reason: Intervention

58. Leow NM, Moreno F, Marletta D, Hussain SB, Buti J, Almond N, et al. Recurrence and progression of periodontitis and methods of management in long-term care: A systematic review and meta-analysis. J Clin Periodontol. 2022;49:291–313.

Reason: Intervention

59. Azab M, Ibrahim S, Li A, Khosravirad A, Carrasco-Labra A, Zeng L, et al. Efficacy of secondary vs primary closure techniques for the prevention of postoperative complications after impacted mandibular third molar extractions: A systematic review update and meta-analysis. J Am Dent Assoc. 2022;153:943-956.e48.

Reason: Intervention

60. León-López M, Cabanillas-Balsera D, Martín-González J, Montero-Miralles P, Saúco-Márquez JJ, Segura-Egea JJ. Prevalence of root canal treatment worldwide: A systematic review and meta-analysis. Int Endod J. 2022;55:1105–27.

Reason: Intervention

61. Valdez RMA, Melo TS, Santos-Silva AR, Duarte A, Gueiros LA. Adverse post-operative events of salivary gland biopsies: A systematic review and meta-analysis. J Oral Pathol Med. 2022;51:152–9.

Reason: Intervention

62. Ramanauskaite A, Schwarz F, Sader R. Influence of width of keratinized tissue on the prevalence of peri-implant diseases: A systematic review and meta-analysis. Clin Oral Implants Res. 2022;33:8–31.

Reason: Intervention

63. Montero E, Molina A, Palombo D, Morón B, Pradíes G, Sanz-Sánchez I. Efficacy and risks of tooth-supported prostheses in the treatment of partially edentulous patients with stage IV periodontitis. A systematic review and meta-analysis. J Clin Periodontol. 2022;49:182–207.

Reason: Intervention

64. Luthra S, Orlandi M, Leira Y, Bokre D, Marletta D, Rotundo R, et al. Invasive dental treatment and acute vascular events: A systematic review and meta-analysis. J Clin Periodontol. 2022;49:467–79.

Reason: Intervention

65. Tay JRH, Ng E, Lu XJ, Lai WMC. Healing complications and their detrimental effects on bone gain in vertical-guided bone regeneration: A systematic review and meta-analysis. Clin Implant Dent Relat Res. 2022;24:43–71.

Reason: Intervention

66. Gennai S, Izzetti R, Pioli MC, Music L, Graziani F. Impact of rehabilitation versus edentulism on systemic health and quality of life in patients affected by periodontitis: A systematic review and meta-analysis. J Clin Periodontol. 2022;49:328–58.

Reason: Intervention

67. Belotti L, Frazão P. Effectiveness of water fluoridation in an upper-middle-income country: A systematic review and meta-analysis. Int J Paediatr Dent. 2022;32:503–13.

Reason: Intervention

68. Singh AK, Khanal N, Acharya N, Rokaya D, Hasan MR, Saito T. Are Physics Forceps Less Traumatic than Conventional Forceps for Tooth Extraction? A Systematic Review and Meta-Analysis of Randomized Controlled Trials. Dentistry J. 2022;10.

Reason: Intervention

69. Almutairi W, Al-Dahman Y, Alnassar F, Albalawi O. Intracanal calcification following regenerative endodontic treatment: a systematic review and meta-analysis. Clin Oral Invest. 2022;26:3333–42.

Reason: Intervention

70. Dalle Carbonare M, Manisali M. Surgical management of syndromic versus non-syndromic craniofacial fibrous dysplasia: a systematic review and meta-analysis. Br J Oral Maxillofac Surg. 2022;60:1166–75.

Reason: Intervention

71. Charlotte Höfer K, Graf I, Adams A, Kuhr K, Plum G, Schwendicke F, et al. Bacteraemia of oral origin in children—A Systematic review and network meta-analysis. Oral Dis. 2022;28:1783–801.

Reason: Intervention

72. Nardello LCL, Pinheiro ET, Gavini G, Prado LC, Romero RX, Gomes BPFA, et al. Nature and Prevalence of Bacterial Taxa Persisting after Root Canal Chemomechanical Preparation in Permanent Teeth: A Systematic Review and Meta-analysis. J Endod. 2022;48:572–96.

Reason: Intervention

73. Traboulsi-Garet B, Jorba-García A, Camps-Font O, Alves FA, Figueiredo R, Valmaseda-Castellón E. Is serum C-terminal telopeptide cross-link of type 1 collagen a reliable parameter for predicting the risk of medication-related osteonecrosis of the jaws? A systematic review and meta-analysis of diagnostic test accuracy. Clin Oral Invest. 2022;26:2371–82.

Reason: Intervention

74. Garcia-Sanchez R, Dopico J, Kalemaj Z, Buti J, Pardo Zamora G, Mardas N. Comparison of clinical outcomes of immediate versus delayed placement of dental implants: A systematic review and meta-analysis. Clin Oral Implants Res. 2022;33:231–77.

Reason: Intervention

75. Manicone PF, De Angelis P, Rella E, Papetti L, D’Addona A. Proximal Contact Loss in Implant-Supported Restorations: A Systematic Review and Meta-Analysis of Prevalence. J Prosthodontics. 2022;31:201–9.

Reason: Intervention

76. Josic U, Mazzitelli C, Maravic T, Ragazzini N, Jacimovic J, Mancuso E, et al. Universal adhesives and non-carious cervical lesions. A systematic review and meta-analysis. Dent Cadmos. 2022;90:188–97.

Reason: Intervention

77. Kohli S, Bhatia S, Al-Haddad A, Pulikkotil SJ, Jamayet NB. Pulpal and Periapical Status of the Vital Teeth Used as Abutment for Fixed Prosthesis—A Systematic Review and Meta-Analysis. J Prosthodontics. 2022;31:102–14.

Reason: Intervention

78. Costantino A, Festa BM, Ferreli F, Russo E, Malvezzi L, Giannitto C, et al. Circumferential pharyngeal reconstruction after total laryngopharyngectomy: A systematic review and network meta-analysis. Oral Oncol. 2022;127.

Reason: Intervention

79. Lopes T, Grillo R, de Almeida Milani B, Jodas CRP, Teixeira RG. Complications on maxilar impaction: A systematic review and meta-analysis. J Stomatol Oral Max Surg. 2022;123:e268–72.

Reason: Intervention

80. Papageorgiou SN, Antonoglou GN, Michelogiannakis D, Kakali L, Eliades T, Madianos P. Effect of periodontal–orthodontic treatment of teeth with pathological tooth flaring, drifting, and elongation in patients with severe periodontitis: A systematic review with meta-analysis. J Clin Periodontol. 2022;49:102–20.

Reason: Intervention

81. Bitencourt FV, Lia EN, Pauletto P, Martins CC, Stefani CM, Massignan C, et al. Prevalence of SARS-CoV-2 infection among oral health care workers worldwide: A meta-analysis. Community Dent Oral Epidemiol. 2022. https://doi.org/10.1111/cdoe.12827.

Reason: Not related to topic

82. Cetira Filho EL, Sales PHH, Rebelo HL, Silva PGB, Maffìa F, Vellone V, et al. Do lower third molars increase the risk of complications during mandibular sagittal split osteotomy? Systematic review and meta-analysis. Int J Oral Maxillofac Surg. 2022;51:906–21.

Reason: Intervention

83. Tang M, Gurpegui Abud D, Shariff JA. Oral Contraceptive Use and Alveolar Osteitis Following Third Molar Extraction: A Systematic Review and Meta-Analysis. Int J Dent. 2022;2022.

Reason: Intervention

84. Linn YL, Khoo MSQ, Sultana R, Iyer NG, Dharmawan R. Comparison of the use of allogenic acellular dermal matrix on rates of Frey syndrome post parotidectomy: A systematic review and meta-analysis. Oral Surg Oral Med Oral Pathol Oral Radiol. 2022;134:49–56.

Reason: Intervention

85. Andrade CAS, Paz JLC, de Melo GS, Mahrouseh N, Januário AL, Capeletti LR. Survival rate and peri-implant evaluation of immediately loaded dental implants in individuals with type 2 diabetes mellitus: a systematic review and meta-analysis. Clin Oral Invest. 2022;26:1797–810.

Reason: Intervention

86. Sabeti M, Huang Y, Chung YJ, Azarpazhooh A. Prognosis of Vital Pulp Therapy on Permanent Dentition: A Systematic Review and Meta-analysis of Randomized Controlled Trials. J Endod. 2021;47:1683–95.

Reason: Intervention

87. Zou H-W, Gao J, Liu JX, Qu Z-L, Du Z-S, Zhao H, et al. Feasibility and advantages of endoscope-assisted parotidectomy: a systematic review and meta-analysis. Br J Oral Maxillofac Surg. 2021;59:503–10.

Reason: Intervention

88. Dos Santos MH, Dos Santos LR, Alves LNS, Moura HS, de Carvalho MMSG, Lins RBE. Do Bulk-Fill Resin Composites Present More Susceptibility to Marginal Degradation in Different Clinical Scenarios? A Systematic Review and Meta-Analysis. Pesqui Bras Odontopediatria Clin Integr. 2022;22.

Reason: Intervention

89. Ferro A, Kotecha S, Auzinger G, Yeung E, Fan K. Systematic review and meta-analysis of tracheostomy outcomes in COVID-19 patients. Br J Oral Maxillofac Surg. 2021;59:1013–23.

Reason: Not related to topic and Intervention

90. Esteves Lima RP, Abreu LG, Belém FV, Pereira GHDM, Brant RA, Costa FO. Is Implantoplasty Efficacious at Treating Peri-Implantitis? A Systematic Review and Meta-Analysis. J Oral Maxillofac Surg. 2021;79:2270–9.

Reason: Intervention

91. Ribeiro MCDO, Vargas-Moreno VF, Gomes RS, Faot F, Del Bel Cury AA, Marcello-Machado RM. Implant-supported crowns with locking taper implant-abutment connection: A systematic review and meta-analysis. J Prosthet Dent. 2022. https://doi.org/10.1016/j.prosdent.2022.06.005.

Reason: Intervention

92. Abdelhay N, Prasad S, Gibson MP. Failure rates associated with guided versus non-guided dental implant placement: a systematic review and meta-analysis. BDJ Open. 2021;7.

Reason: Intervention

93. Ferreira MS, Miranda G, Almeida FT, Gasperini G, Silva BSDF, Valladares-Neto J, et al. Complications in intraoral versus external approach for surgical treatment of Eagle syndrome: A systematic review and meta-analysis. Cranio J Craniomandibular Prac. 2022. https://doi.org/10.1080/08869634.2021.2020995.

Reason: Intervention

94. Proaño-Haro A, Bagan L, Bagan JV. Recurrences following treatment of proliferative verrucous leukoplakia: A systematic review and meta-analysis. J Oral Pathol Med. 2021;50:820–8.

Reason: Intervention

95. Oliveira CDS, Galdino TM, Limeira FIR, Moreira AN, de Magalhães CS, Abreu LG. Is dental caries associated with liver transplantation? A systematic review and meta-analysis. Oral Dis. 2021;27:1346–55.

Reason: Intervention

96. Seehra J, Stonehouse-Smith D, Pandis N. Prediction intervals reporting in orthodontic meta-analyses. Eur J Orthod. 2021;43:596–600.

Reason: Other study design

97. Ferrillo M, Nucci L, Giudice A, Calafiore D, Marotta N, Minervini G, et al. Efficacy of conservative approaches on pain relief in patients with temporomandibular joint disorders: a systematic review with network meta-analysis. Cranio J Craniomandibular Sleep Prac. 2022. https://doi.org/10.1080/08869634.2022.2126079.

Reason: Intervention

98. Santana LG, Avelar K, Marques LS. Association between arch perimeter management and the occurrence of mandibular second molar eruption disturbances: Systematic review and meta-analysis. Angle Orthod. 2021;91:544–54.

Reason: Intervention

99. Ramis-Alario A, Soto-Peñaloza D, Tarazona-Alvarez B, Peñarrocha-Diago M, Peñarrocha-Oltra D. Comparison of the diagnostic efficacy of 2D radiography and cone beam computed tomography in persistent apical periodontal disease: A PRISMA-DTA systematic review and meta-analysis. Oral Surg Oral Med Oral Pathol Oral Radiol. 2021;132:e153–68.

Reason: Intervention

100. Shafizadeh M, Amid R, Mahmoum M, Kadkhodazadeh M. Histopathological characterization of peri-implant diseases: A systematic review and meta-analysis. Arch Oral Biol. 2021;132.

Reason: Intervention

101. Shen Loo Y, Yee Wong T, Veettil SK, Se Wong P, Gopinath D, Mooi Ching S, et al. Antifungal agents in preventing oral candidiasis in clinical oncology: A network meta-analysis. Oral Dis. 2021;27:1631–43.

Reason: Intervention and other study design

102. Stanek J, Riad A, Slezakova S, Azar B, Klugarova J, Pokorna A, et al. Bar fracture of implant-retained overdenture: Protocol of a systematic review and meta-analysis. J Osseointegration. 2021;13:89–94.

Reason: Intervention

103. Beaumont S, Bhatia N, McDowell L, Fua T, McCullough M, Celentano A, et al. Timing of dental extractions in patients undergoing radiotherapy and the incidence of osteoradionecrosis: a systematic review and meta-analysis. Br J Oral Maxillofac Surg. 2021;59:511–23.

Reason: Intervention

104. Thorat NC, Sahana S, Chauhan N, Singh TP, Khare A. Prevalence of musculoskeletal pain in dentists; A systematic review and meta-analysis. J Head Neck Physician Surg. 2022;10:53–60.

Reason: Not related to topic

105. Theodoridis C, Doulkeridou C, Menexes G, Vouros I. Comparison of RANKL and OPG levels in peri-implant crevicular fluid between healthy and diseased peri-implant tissues. A systematic review and meta-analysis. Clin Oral Invest. 2022;26:823–36.

Reason: Intervention

106. Fiacchini G, Benettini G, Tricò D, Torregrossa L, Vianini M, Picariello M, et al. Human papillomavirus-related head and neck adenosquamous carcinoma: A systematic review and individual patient data meta-analysis. Oral Oncol. 2021;119.

Reason: Not related to topic and Intervention

107. Yuan L, Gao J, Liu S, Zhao H. Does the Lingual-Based Mucoperiosteal Flap Reduce Postoperative Morbidity Compared With the Buccal-Based Mucoperiosteal Flap After the Surgical Removal of Impacted Third Molars? A Meta-analysis Review. J Oral Maxillofac Surg. 2021;79:1409-1421.e3.

Reason: Intervention

108. Rodrigues-Fernandes CI, Abreu LG, Radhakrishnan R, Perez DEDC, Amaral-Silva GK, Gondak RDO, et al. Prognostic significance of CD30 expression in diffuse large B-cell lymphoma: A systematic review with meta-analysis. J Oral Pathol Med. 2021;50:587–93.

Reason: Not related to topic

109. Ferreli F, Festa BM, Costantino A, Malvezzi L, Colombo G, Spriano G, et al. Prevalence of occult level 2b nodal metastases in cN0 squamous cell carcinoma of the oral cavity: A systematic review and meta-analysis. Oral Oncol. 2021;122.

Reason: Not related to topic

110. Toneatti DJ, Graf RR, Burkhard J-P, Schaller B. Survival of dental implants and occurrence of osteoradionecrosis in irradiated head and neck cancer patients: a systematic review and meta-analysis. Clin Oral Invest. 2021;25:5579–93.

Reason: Intervention

111. Vandenberg K, Castle M, Qeadan F, Kraai T. Oronasal Fistula Incidence Associated With Vomer Flap Repair of Cleft Palate: A Systematic Review and Meta-Analysis. Cleft Palate-Craniofac J. 2021;58:957–65.

Reason: Intervention

112. AlSaleh E, Dutta A, Dummer PMH, Farnell DJJ, Vianna ME. Influence of remaining axial walls on of root filled teeth restored with a single crown and adhesively bonded fibre post: A systematic review and meta-analysis. J Dent. 2021;114.

Reason: Intervention

113. Yu L, Yu X, Li Y, Yang F, Hong J, Qin D, et al. The additional benefit of professional fluoride application for children as an adjunct to regular fluoride toothpaste: a systematic review and meta-analysis. Clin Oral Invest. 2021;25:3409–19.

Reason: Intervention

114. Nasiri K, Wrbas K-T. Accuracy of different generations of apex locators in determining working length; a systematic review and meta-analysis. Saudi Dent J. 2022;34:11–20.

Reason: Intervention

115. Didilescu AC, Vacaru R-P, Pronk C, Scheau C, Lazu A, Dan LP, et al. Oral diseases after liver transplantation: a systematic review and meta-analysis. Brit Dent J. 2021;231:117–24.

Reason: Intervention

116. Namdari M, Amdjadi P, Bayat A, Seifi M, Alzwghaibi A. Comparison of the failure rate, bonding time and ARI score of two orthodontic bonding systems: Self-Etch Primer and Conventional Etching Primer: A systematic review and meta-analysis. Int Orthod. 2021;19:566–79.

Reason: Intervention

117. Nanditha Kumar M, Raghavendra Swamy KN, Thippeswamy HM, Kamath G, Devananda D. Prevalence of xerostomia in patients on haemodialysis: A systematic review and meta-analysis. Gerodontology. 2021;38:235–41.

Reason: Intervention

118. Aviandiva M, Putri DS, Sulijaya B, Masulili SLC, Lessang R. Clinical Periodontal Parameter of Smokers with Periodontitis in Asia Following Periodontal Therapy: An Update Systematic Review and Meta-Analysis. J Int Dent Med Res. 2022;15:926–34.

Reason: Intervention

119. Fernández CE, Maturana CA, Coloma SI, Carrasco-Labra A, Giacaman RA. Teledentistry and mHealth for Promotion and Prevention of Oral Health: A Systematic Review and Meta-analysis. J Dent Res. 2021;100:914–27.

Reason: Not related to topic

120. Meire MA, Bronzato JD, Bomfim RA, Gomes BPFA. Effectiveness of adjunct therapy for the treatment of apical periodontitis: A systematic review and meta-analysis. Int Endod J. 2022. https://doi.org/10.1111/iej.13838.

Reason: Intervention

121. Nagay BE, Dini C, Borges GA, Mesquita MF, Cavalcanti YW, Magno MB, et al. Clinical efficacy of anodized dental implants for implant-supported prostheses after different loading protocols: A systematic review and meta-analysis. Clin Oral Implants Res. 2021;32:1021–40.

Reason: Intervention

122. de Kuijper MCFM, Cune MS, Özcan M, Gresnigt MMM. Clinical performance of direct composite resin versus indirect restorations on endodontically treated posterior teeth: A systematic review and meta-analysis. J Prosthet Dent. 2022. https://doi.org/10.1016/j.prosdent.2021.11.009.

Reason: Intervention

123. Adekunle AA, Egbunah UP, Erinoso OA, Adeyemo WL. Effectiveness of warm saline mouth bath in preventing alveolar osteitis: A systematic review and meta-analysis. J Cranio-Maxillofac Surg. 2021;49:980–8.

Reason: Intervention

124. Reia VCB, de Toledo Telles-Araujo G, Peralta-Mamani M, Biancardi MR, Rubira CMF, Rubira-Bullen IRF. Diagnostic accuracy of CBCT compared to panoramic radiography in predicting IAN exposure: a systematic review and meta-analysis. Clin Oral Invest. 2021;25:4721–33.

Reason: Intervention

125. Makou O, Eliades T, Koletsi D. Reporting, interpretation, and extrapolation issues (SPIN) in abstracts of orthodontic meta-analyses published from 2000 to 2020. Eur J Orthod. 2021;43:567–75.

Reason: Other Study Design

126. Abdel-Halim CN, Rosenberg T, Dyrvig A-K, Høilund-Carlsen PF, Sørensen JA, Rohde M, et al. Diagnostic accuracy of imaging modalities in detection of histopathological extranodal extension: A systematic review and meta-analysis. Oral Oncol. 2021;114.

Reason: Not related to topic

127. Shams Abadi MS, Taji A, Salehi F, Kazemian H, Heidari H. High-level Gentamicin Resistance among Clinical Isolates of Enterococci in Iran: a Systematic Review and Meta-analysis. Folia Med. 2021;63:15–23.

Reason: Not related to topic

128. Gupta A, Sharda S, Shafiq N, Kumar A, Goyal A. Topical fluoride-antibacterial agent combined therapy versus topical fluoride monotherapy in preventing dental caries: a systematic review and meta-analysis. Eur Arch Paediatr Dent. 2020;21:629–46.

Reason: Intervention

129. Jakovljevic A, Nikolic N, Jacimovic J, Pavlovic O, Milicic B, Beljic-Ivanovic K, et al. Prevalence of Apical Periodontitis and Conventional Nonsurgical Root Canal Treatment in General Adult Population: An Updated Systematic Review and Meta-analysis of Cross-sectional Studies Published between 2012 and 2020. J Endod. 2020;46:1371-1386.e8.

Reason: Intervention

130. Hao S, Wang J, Wang Y. Effectiveness and safety of Bifidobacterium in preventing dental caries: a systematic review and meta-analysis. Acta Odontol Scand. 2021;79:613–22.

Reason: Intervention

131. Askar H, Misch J, Chen Z, Chadha S, Wang H-L. Capnography monitoring in procedural intravenous sedation: a systematic review and meta-analysis. Clin Oral Invest. 2020;24:3761–70.

Reason: Intervention

132. Neto JCL, Fernandes LM, Magno MB, Lima TFR, De Dantas De Almeida LF, Santiago BM, et al. The effect of reciprocating and rotary systems on postoperative endodontic pain: A systematic review and meta-analysis. Iran Endod J. 2020;15:198–210.

Reason: Intervention

133. Yu X, Han Y, Wang J. Is an internal tapered connection more efficient than an internal nontapered connection? A systematic review and meta-analysis. J Prosthet Dent. 2020;124:431–8.

Reason: Intervention

134. Hu H, Feng C, Jiang Z, Wang L, Shrestha S, Yan J, et al. Effectiveness of remineralizing agents in the prevention and reversal of orthodontically induced white spot lesions: a systematic review and network meta-analysis. Clin Oral Invest. 2020;24:4153–67.

Reason: Intervention

135. Zhu J, Zhang S, Yuan X, He T, Liu H, Wang J, et al. Effect of platelet-rich fibrin on the control of alveolar osteitis, pain, trismus, soft tissue healing, and swelling following mandibular third molar surgery: an updated systematic review and meta-analysis. Int J Oral Maxillofac Surg. 2021;50:398–406.

Reason: Intervention

136. Jain A, Rai A. Meta-Analysis to Evaluate the Efficacy of Sternocleidomastoid Muscle Flap as a Reconstruction Modality in Prevention of Frey’s Syndrome Following Parotidectomy. J Maxillofac Oral Surg. 2021;20:310–8.

Reason: Intervention and other study design

137. de Pauli Paglioni M, Migliorati CA, Schausltz Pereira Faustino I, Linhares Almeida Mariz BA, Oliveira Corrêa Roza AL, Agustin Vargas P, et al. Laser excision of oral leukoplakia: Does it affect recurrence and malignant transformation? A systematic review and meta-analysis. Oral Oncol. 2020;109.

Reason: Intervention

138. Banu S, Pratibha G, Rajeshwari HR, Ravishankar N. LOCAL DELIVERY OF TETRACYCLINE GROUP OF DRUGS AS ADJUNCTS TO MECHANICAL DEBRIDEMENT IN PERI-IMPLANTITIS: A SYSTEMATIC REVIEW AND META-ANALYSIS. J Stomatol. 2021;74:124–31.

Reason: Intervention

139. Chaga MM, Kobayashi-Velasc S, Gimene T, Cavalcant MGP. Diagnostic accuracy of imaging examinations for peri-implant bone defects around titanium and zirconium dioxide implants: A systematic review and meta-analysis. Imaging Sci Dent. 2021;51:363–72.

Reason: Intervention

140. Rahbani Nobar B, Dianat O, Rahbani Nobar B, Shirvani A, Zargar N, Kazem M, et al. Effect of rotary and reciprocating instrumentation motions on postoperative pain incidence in non-surgical endodontic treatments: A systematic review and meta-analysis. Eur Endod J. 2021;6:3–14.

Reason: Intervention

141. Ruksakiet K, Hanák L, Farkas N, Hegyi P, Sadaeng W, Czumbel LM, et al. Antimicrobial Efficacy of Chlorhexidine and Sodium Hypochlorite in Root Canal Disinfection: A Systematic Review and Meta-analysis of Randomized Controlled Trials. J Endod. 2020;46:1032-1041.e7.

Reason: Intervention

142. Xu X, Huang J, Fu X, Kuang Y, Yue H, Song J, et al. Short implants versus longer implants in the posterior alveolar region after an observation period of at least five years: A systematic review and meta-analysis. J Dent. 2020;100.

Reason: Intervention

143. Yon MJ-Y, Tang MH-Y, Cheung GS-P. Defects and Safety of NiTi Root Canal Instruments: A Systematic Review and Meta-Analysis. Front Dent Med. 2021;2.

Reason: Intervention

144. Lan K, Wang F, Huang W, Davó R, Wu Y. Quad Zygomatic Implants: A Systematic Review and Meta-analysis on Survival and Complications. Int J Oral Maxillofac Implants. 2021;36:21–9.

Reason: Intervention

145. Nath S, Mupparapu M. Calcified carotid artery atheroma and stroke risk assessment. Use of Doppler ultrasonography as a secondary marker: A meta-analysis. Quintessence Int. 2021;52:348–59.

Reason: Not related to topic and other study design

146. Gupta A, Sharda S, Kumar A, Goyal A, Gauba K. Comparing the effectiveness of topical fluoride and povidone iodine with topical fluoride alone for the prevention of dental caries among children: A systematic review and meta-analysis. Int J Clin Pediatr Dent. 2020;13:559–65.

Reason: Intervention

147. Tu X, Ren J, Zhao Y. Prognostic value of prognostic nutritional index in nasopharyngeal carcinoma: A meta-analysis containing 4511 patients. Oral Oncol. 2020;110.

Reason: Not related to topic

148. Ierardo G, Mazur M, Luzzi V, Calcagnile F, Ottolenghi L, Polimeni A. Treatments of sleep bruxism in children: A systematic review and meta-analysis. Cranio J Craniomandibular Prac. 2021;39:58–64.

Reason: Intervention

149. Silveira ER, Cademartori MG, Schuch HS, Armfield JA, Demarco FF. Estimated prevalence of dental fear in adults: A systematic review and meta-analysis. J Dent. 2021;108.

Reason: Not related to topic

150. Weisz Shabtay N, Ronen O. Level IV neck dissection as an elective treatment for oral tongue carcinoma—a systematic review and meta-analysis. Oral Surg Oral Med Oral Pathol Oral Radiol. 2020;130:363–72.

Reason: Intervention

151. Kastelic DRA, Volpato LER, de Campos Neves ATS, Aranha AM, Martins CC. Do children and adolescents prefer pediatric attire over white attire during dental appointments? A meta-analysis of prevalence data. Int J Clin Pediatr Dent. 2021;14:14–29.

Reason: Not related to topic

152. Canellas JVDS, Fraga SRG, Santoro MF, Netto JDNS, Tinoco EMB. Intrasocket interventions to prevent alveolar osteitis after mandibular third molar surgery: A systematic review and network meta-analysis. J Cranio-Maxillofac Surg. 2020;48:902–13.

Reason: Intervention

153. Sharda S, Gupta A, Goyal A, Gauba K. Remineralization potential and caries preventive efficacy of CPP-ACP/Xylitol/Ozone/Bioactive glass and topical fluoride combined therapy versus fluoride mono-therapy–a systematic review and meta-analysis. Acta Odontol Scand. 2021;79:402–17.

Reason: Intervention

154. Gupta A, Aggarwal V, Mehta N, Abraham D, Singh A. Diabetes mellitus and the healing of periapical lesions in root filled teeth: a systematic review and meta-analysis. Int Endod J. 2020;53:1472–84.

Reason: Intervention

155. Muniz FWMG, Maurique LS, Toniazzo MP, Silva CF, Casarin M. Self-reported depressive symptoms in dental students: Systematic review with meta-analysis. J Dent Educ. 2021;85:135–47.

Reason: Not related to topic

156. Bedran NR, Nadelman P, Magno MB, de Almeida Neves A, Ferreira DM, Braga Pintor AV, et al. Does Calcium Hydroxide Reduce Endotoxins in Infected Root Canals? Systematic Review and Meta-analysis. J Endod. 2020;46:1545–58.

Reason: Intervention

157. Liu M, He L, Wang H. Clinical and radiographic performance of one-piece and two-piece implant: A systematic review and meta-analysis. J Prosthodontic Res. 2021;65:56–66.

Reason: Intervention

158. Díez-Suárez L, Paredes-Farrera GF. Dislocation of the mandibular condyle into the middle cranial fossa. A case of temporomandibular joint arthroplasty with resorbable fixation system and temporalis myofascial flap: systematic review and meta–analysis. Br J Oral Maxillofac Surg. 2021;59:389–97.

Reason: Intervention

159. de Araujo CM, Trannin PD, Schroder AGD, Stechman-Neto J, Cavalcante-Leão BL, Mattos NHR, et al. Surgical-Periodontal aspects in orthodontic traction of palatally displaced canines: a meta-analysis. Jpn Dent Sci Rev. 2020;56:164–76.

Reason: Intervention and Other study design

160. Moori PL, Rahman S. Endoscopic versus conventional parotid gland excision: a systematic review and meta-analysis. Br J Oral Maxillofac Surg. 2021;59:272–80.

Reason: Intervention

161. Peng J, Shi Y, Wang J, Wang F, Dan H, Xu H, et al. Low-level laser therapy in the prevention and treatment of oral mucositis: a systematic review and meta-analysis. Oral Surg Oral Med Oral Pathol Oral Radiol. 2020;130:387-397.e9.

Reason: Intervention

162. Thomas N, Blake S. Dental disease risk in children with autism: a meta-analysis. Evid-based Dent. 2021;22:34–5.

Reason: Other study design

163. Pellegrino G, Ferri A, Fabbro MD, Prati C, Gandolfi MG, Marchetti C. Dynamic Navigation in Implant Dentistry: A Systematic Review and Meta-analysis. Int J Oral Maxillofac Implants. 2021;36:121–40.

Reason: Intervention

164. da Rocha SS, Sales PHDH, Carvalho PHR, Maia RN, Gondim RF, de Menezes Junior JMS, et al. Mandibular traumas by gunshot. A systematic review with meta-analysis and algorithm of treatment. Br J Oral Maxillofac Surg. 2021;59:e99–108.

Reason: Intervention

165. Pacha MM, Fleming PS, Johal A. Complications, impacts, and success rates of different approaches to treatment of Class II malocclusion in adolescents: A systematic review and meta-analysis. Am J Orthod Dentofacial Orthop. 2020;158:477-494.e7.

Reason: Intervention

166. Song X, Li L, Gou H, Xu Y. Impact of implant location on the prevalence of peri-implantitis: A systematic review and meta- analysis. J Dent. 2020;103.

Reason: Intervention

167. Martins OP, Baptista IP, Caramelo FJ. Disease recurrence after surgical treatment of peri-implantitis —systematic review and meta-analysis. Frontier Oral Maxillofac Med. 2021;3.

Reason: Intervention

168. Zhang Y, Chen W, Zhang J, Li Y. Does Er,Cr:YSGG reduce the microleakage of restorations when used for cavity preparation A systematic review and meta-analysis. BMC Oral Health. 2020;20.

Reason: Intervention

169. Garip M, Van Dessel J, Grosjean L, Politis C, Bila M. The impact of smoking on surgical complications after head and neck reconstructive surgery with a free vascularised tissue flap: a systematic review and meta-analysis. Br J Oral Maxillofac Surg. 2021;59:e79–98.

Reason: Not related to topic and Intervention

170. Iezzi G, Perrotti V, Felice P, Barausse C, Piattelli A, Del Fabbro M. Are <7-mm long implants in native bone as effective as longer implants in augmented bone for the rehabilitation of posterior atrophic jaws? A systematic review and meta-analysis. Clin Implant Dent Relat Res. 2020;22:552–66.

Reason: Intervention

171. Lins RBE, Sebold M, Magno MB, Maia LC, Martins LRM, Giannini M. Does the type of solvent in dental adhesives influence the clinical performance of composite restorations placed in noncarious cervical lesions? a systematic review and meta-analysis. Oper Dent. 2020;45:E237–54.

Reason: Intervention

172. Xiang X, Shi P, Zhang P, Shen J, Kang J. Impact of platelet-rich fibrin on mandibular third molar surgery recovery: A systematic review and meta-analysis. BMC Oral Health. 2019;19.

Reason: Intervention

173. de Almeida-Pinto YD, Costa SFDS, de Andrade BAB, Altemani A, Vargas PA, Abreu LG, et al. t(6;9)(MYB-NFIB) in head and neck adenoid cystic carcinoma: A systematic review with meta-analysis. Oral Dis. 2019;25:1277–82.

Reason: Not related to topic

174. Timms L, Deery C. Fluoride varnish and dental caries in preschoolers: a systematic review and meta-analysis. Evid-based Dent. 2020;21:18–9.

Reason: Intervention

175. Lam PPY, Sardana D, Ekambaram M, Lee GHM, Yiu CKY. Effectiveness of Pit and Fissure Sealants for Preventing and Arresting Occlusal Caries in Primary Molars: A Systematic Review and Meta-Analysis. J Evid-Based Dent Pract. 2020;20.

Reason: Intervention

176. Satheeshkumar PS, Papatheodorou S, Sonis S. Enhanced oral hygiene interventions as a risk mitigation strategy for the prevention of non-ventilator-associated pneumonia: a systematic review and meta-analysis. Brit Dent J. 2020;228:615–22.

Reason: Intervention

177. Jamali S, Nasrabadi N, Payahoo S, Darvish M, Ahmadizadeh H, Khosravi S. Management of the maxillary sinus complications after dental implantation: A systematic review and meta-analysis. Braz Dent Sci. 2020;23.

Reason: Intervention

178. Wusiman P, Abasi K, Maimaitishawuti D, Moming A. Management of Mandibular Angle Fractures Using One Miniplate or Two Miniplate Fixation System: A Systematic Review and Meta-Analysis. J Oral Maxillofac Surg. 2019;77:1673.e1-1673.e11.

Reason: Intervention

179. Koç S, Del Fabbro M. Does the Etiology of Pulp Necrosis Affect Regenerative Endodontic Treatment Outcomes? A Systematic Review and Meta-analyses. J Evid-Based Dent Pract. 2020;20.

Reason: Intervention

180. Jazayeri HE, Khavanin N, Yu JW, Lopez J, Shamliyan T, Peacock ZS, et al. Fixation Points in the Treatment of Traumatic Zygomaticomaxillary Complex Fractures: A Systematic Review and Meta-Analysis. J Oral Maxillofac Surg. 2019;77:2064–73.

Reason: Intervention

181. Deepthi R, Bilichodmath S. Ozone therapy in periodontics: A meta-analysis. Contemp Clin Dent. 2020;11:108–15.

Reason: Intervention

182. Zhou X, Yang J, Wu L, Tang X, MOU Y, Sun W, et al. Evaluation of the Effect of Implants Placed in Preserved Sockets Versus Fresh Sockets on Tissue Preservation and Esthetics: A Meta-analysis and Systematic Review. J Evid-Based Dent Pract. 2019;19.

Reason: Intervention

183. Wang B-C, Cao R-B, Fu C, Chen W-B, Li P-D, Lin G-H, et al. The efficacy and safety of PD-1/PD-L1 inhibitors in patients with recurrent or metastatic nasopharyngeal carcinoma: A systematic review and meta-analysis. Oral Oncol. 2020;104.

Reason: Not related to topic and Intervention

184. Carneiro-Campos LE, Freitas-Fernandes LB, Masterson D, Magno MB, Fernandes CP, Maia LC, et al. Does the natural maxillary dentition influence the survival rate of mandibular metal-resin implant-supported fixed complete dentures? A systematic review and meta-analysis. J Prosthet Dent. 2020;124:36–45.

Reason: Intervention

185. Bagegni A, Abou-Ayash S, Rücker G, Algarny A, Att W. The influence of prosthetic material on implant and prosthetic survival of implant-supported fixed complete dentures: a systematic review and meta-analysis. J Prosthodontic Res. 2019;63:251–65.

Reason: Intervention

186. Westergaard-Nielsen M, Rosenberg T, Gerke O, Dyrvig A-K, Godballe C, Bjørndal K. Elective neck dissection in patients with salivary gland carcinoma: A systematic review and meta-analysis. J Oral Pathol Med. 2020;49:606–16.

Reason: Intervention

187. Borges H, Correia ARM, Castilho RM, Fernandes GVO. Zirconia implants and marginal bone loss: A systematic review and meta-analysis of clinical studies. Int J Oral Maxillofac Implants. 2020;35:707–20.

Reason: Intervention

188. Song Q, Li F, Chen X, Wang J, Liu H, Cheng Y. Early detection treatment response for head and neck carcinomas using intravoxel incoherent motion-magnetic resonance imaging: A meta-analysis. Dentomaxillofac Radiol. 2020;50.

Reason: Not related to topic and other study design

189. Khan AR, Fida M, Gul M. Decalcification and bond failure rate in resin modified glass ionomer cement versus conventional composite for orthodontic bonding: A systematic review & meta-analysis. Int Orthod. 2020;18:32–40.

Reason: Intervention

190. de Toledo Telles-Araújo G, Peralta-Mamani M, Caminha RDAG, de Fatima Moraes-da-Silva A, Rubira CMF, Honório HM, et al. CBCT does not reduce neurosensory disturbances after third molar removal compared to panoramic radiography: a systematic review and meta-analysis. Clin Oral Invest. 2020;24:1137–49.

Reason: Intervention

191. Zhu J, Yuan X, Yan L, Li T, Guang M, Zhang Y. Comparison of Postoperative Outcomes Between Envelope and Triangular Flaps After Mandibular Third Molar Surgery: A Systematic Review and Meta-Analysis. J Oral Maxillofac Surg. 2020;78:515–27.

Reason: Intervention

192. Yin S-C, Su X-Z, So HI, Wang S, Zhang Z-L, Xu Z-F, et al. Comparison of Internal Jugular Vein System Anastomosis and External Jugular Vein System Anastomosis in Free Flaps for Head and Neck Reconstruction: A Meta-Analysis. J Oral Maxillofac Surg. 2020;78:142–52.

Reason: Intervention and not related to topic

193. Limones A, Sáez-Alcaide L-M, Díaz-Parreño S-A, Helm A, Bornstein M-M, Molinero-Mourelle P. Medication-related osteonecrosis of the jaws (MRONJ) in cancer patients treated with denosumab VS. zoledronic acid: A systematic review and meta-analysis. Med Oral Patol Oral Cir Bucal. 2020;25:e326–36.

Reason: Intervention

194. Martins-Andrade B, dos Santos Costa SF, Sant’ana MSP, Altemani A, Vargas PA, Fregnani ER, et al. Prognostic importance of the lymphovascular invasion in head and neck adenoid cystic carcinoma: A systematic review and meta-analysis. Oral Oncol. 2019;93:52–8.

Reason: Not related to topic

195. Chen J, Cai M, Yang J, Aldhohrah T, Wang Y. Immediate versus early or conventional loading dental implants with fixed prostheses: A systematic review and meta-analysis of randomized controlled clinical trials. J Prosthet Dent. 2019;122:516–36.

Reason: Intervention

196. Cai H, Zhu Y, Wang C, Zhang Y, Hou J. Neck nodal recurrence and survival of clinical T1-2 N0 oral squamous cell carcinoma in comparison of elective neck dissection versus observation: A meta-analysis. Oral Surg Oral Med Oral Pathol Oral Radiol. 2020;129:296–310.

Reason: Intervention

197. Zeng BS, Lin SY, Tu YK, Wu YC, Stubbs B, Liang CS, et al. Prevention of Postdental Procedure Bacteremia: A Network Meta-analysis. J Dent Res. 2019;98:1204–10.

Reason: Intervention and other study design

198. Gorphe P, Simon C. A systematic review and meta-analysis of margins in transoral surgery for oropharyngeal carcinoma. Oral Oncol. 2019;98:69–77.

Reason: Intervention

199. Troiano G, Caponio VCA, Adipietro I, Tepedino M, Santoro R, Laino L, et al. Prognostic significance of CD68+ and CD163+ tumor associated macrophages in head and neck squamous cell carcinoma: A systematic review and meta-analysis. Oral Oncol. 2019;93:66–75.

Reason: Not related to topic

200. Liu S, Li M, Yu J. Does chlorhexidine improve outcomes in non-surgical management of peri-implant mucositis or peri-implantitis?: A systematic review and meta-analysis. Med Oral Patol Oral Cir Bucal. 2020;25:e608–15.

Reason: Intervention

201. Gómez-Barrachina R, Montiel-Company JM, García-Sanz V, Almerich-Silla JM, Paredes-Gallardo V, Bellot-Arcís C. Titanium plate removal in orthognathic surgery: prevalence, causes and risk factors. A systematic literature review and meta-analysis. Int J Oral Maxillofac Surg. 2020;49:770–8.

Reason: Intervention

202. Mehraban SH, Jamali S, Azizi A, Nasrabadi N. Evaluating the effectiveness of orthognathic surgery on the pre-existing temporomandibular disorders in patients with malocclusion: A systematic review and meta-analysis. Pesqui Bras Odontopediatria Clin Integr. 2020;20:1–8.

Reason: Intervention

203. Binmadi NO, Alblowi JA. Prevalence and policy of occupational violence against oral healthcare workers: Systematic review and meta-analysis. BMC Oral Health. 2019;19.

Reason: Not related to topic

204. Lorenzo-Pouso AI, Pérez-Sayáns M, Chamorro-Petronacci C, Gándara-Vila P, López-Jornet P, Carballo J, et al. Association between periodontitis and medication-related osteonecrosis of the jaw: A systematic review and meta-analysis. J Oral Pathol Med. 2020;49:190–200.

Reason: Intervention

205. Tasios T, Papageorgiou SN, Papadopoulos MA, Tsapas A, Haidich A-B. Prevention of orthodontic enamel demineralization: A systematic review with meta-analyses. Orthodont Craniofac Res. 2019;22:225–35.

Reason: Intervention

206. Cao R, Li Q, Wu Q, Yao M, Chen Y, Zhou H. Effect of non-surgical periodontal therapy on glycemic control of type 2 diabetes mellitus: A systematic review and Bayesian network meta-analysis. BMC Oral Health. 2019;19.

Reason: Intervention

207. Shu Z, Li P, Yu B, Huang S, Chen Y. The effectiveness of probiotics in prevention and treatment of cancer therapy-induced oral mucositis: A systematic review and meta-analysis. Oral Oncol. 2020;102.

Reason: Intervention

208. De Sousa FSDO, Dos Santos APP, Nadanovsky P, Hujoel P, Cunha-Cruz J, De Oliveira BH. Fluoride Varnish and Dental Caries in Preschoolers: A Systematic Review and Meta-Analysis. Caries Res. 2019;53:502–13.

Reason: Intervention

209. Padmanabhan H, Kumar A, Shivashankar K. Incidence of neurosensory disturbance in mandibular implant surgery - A meta-analysis. J Indian Prosthodontic Soc. 2020;20:17–26.

Reason: Intervention

210. Polak D, Wilensky A, Antonoglou GN, Shapira L, Goldstein M, Martin C. The efficacy of pocket elimination/reduction compared to access flap surgery: A systematic review and meta-analysis. J Clin Periodontol. 2020;47:303–19.

Reason: Intervention

211. Kurnik NM, Weidler EM, Lien KM, Cordero KN, Williams JL, Temkit M, et al. The Effectiveness of Palate Re-Repair for Treating Velopharyngeal Insufficiency: A Systematic Review and Meta-Analysis. Cleft Palate-Craniofac J. 2020;57:860–71.

Reason: Intervention

212. Teja KV, Vasundhara KA, Sriram G. Antibacterial efficacy of conventional versus herbal products on streptococcus mutans in adult population-a systematic review & meta-analysis. Braz Dent Sci. 2020;23:1–18.

Reason: Intervention

213. de Lima VHS, de Oliveira-Neto OB, da Hora Sales PH, da Silva Torres T, de Lima FJC. Effectiveness of low-level laser therapy for oral mucositis prevention in patients undergoing chemoradiotherapy for the treatment of head and neck cancer: A systematic review and meta-analysis. Oral Oncol. 2020;102.

Reason: Intervention

214. Zhang Y, Wang Y, Chen Y, Chen Y, Zhang Q, Zou J. The clinical effects of laser preparation of tooth surfaces for fissure sealants placement: A systematic review and meta-Analysis. BMC Oral Health. 2019;19.

Reason: Intervention

215. Rodrigues RPCB, de Andrade Vieira W, Siqueira WL, Blumenberg C, de Macedo Bernardino Í, Cardoso SV, et al. Saliva as an alternative to blood in the determination of uremic state in adult patients with chronic kidney disease: a systematic review and meta-analysis. Clin Oral Invest. 2020;24:2203–17.

Reason: Not related to topic

216. Didilescu AC, Lazu A, Pronk C, Vacaru RP, Brand HS. Clinical periodontal and dental findings in liver transplant patients: a systematic review and meta-analysis. Brit Dent J. 2020;228:108–16.

Reason: Intervention

217. Xun Y, Cao Q, Zhang J, Guan B, Wang M. Clinicopathological and prognostic significance of circulating tumor cells in head and neck squamous cell carcinoma: A systematic review and meta-analysis. Oral Oncol. 2020;104.

Reason: Not related to topic

218. von Stein-Lausnitz M, Nickenig H-J, Wolfart S, Neumann K, von Stein-Lausnitz A, Spies BC, et al. Survival rates and complication behaviour of tooth implant–supported, fixed dental prostheses: A systematic review and meta-analysis. J Dent. 2019;88.

Reason: Intervention

219. Jazayeri HE, Khavanin N, Yu JW, Lopez J, Ganjawalla KP, Shamliyan T, et al. Does Early Repair of Orbital Fractures Result in Superior Patient Outcomes? A Systematic Review and Meta-Analysis. J Oral Maxillofac Surg. 2020;78:568–77.

Reason: Intervention

220. Fang X, Qi R, Liu C. Root resorption in orthodontic treatment with clear aligners: A systematic review and meta-analysis. Orthodont Craniofac Res. 2019;22:259–69.

Reason: Intervention

221. Zaki J, Alnawawy M, Yussif N, Elkhadem A. The Effect of Membrane Coverage on the Resorption of Autogenous Intraoral Block Grafts in Horizontal Ridge Augmentation: A Systematic Review of Literature and Meta-Analysis: Inevitability or an Iatrogenic Vulnerability? J Evid-Based Dent Pract. 2018;18:275–89.

Reason: Intervention

222. Lin C-Y, Chen Z, Pan W-L, Wang H-L. The effect of supportive care in preventing peri-implant diseases and implant loss: A systematic review and meta-analysis. Clin Oral Implants Res. 2019;30:714–24.

Reason: Intervention

223. Souza BDM, Dutra KL, Kuntze MM, Bortoluzzi EA, Flores-Mir C, Reyes-Carmona J, et al. Incidence of Root Resorption after the Replantation of Avulsed Teeth: A Meta-analysis. J Endod. 2018;44:1216–27.

Reason: Intervention

224. Liu M, Gai K, Chen J, Jiang L. Comparison of failure and complication risks of porcelain laminate and indirect resin veneer restorations: A meta-analysis. Int J Prosthodontics. 2019;32:59–65.

Reason: Intervention

225. Samandara A, Papageorgiou SN, Ioannidou-Marathiotou I, Kavvadia-Tsatala S, Papadopoulos MA. Evaluation of orthodontically induced external root resorption following orthodontic treatment using cone beam computed tomography (CBCT): A systematic review and meta-analysis. Eur J Orthod. 2019;41:67–79.

Reason: Intervention

226. Papageorgiou SN, Xavier GM, Cobourne MT, Eliades T. Effect of orthodontic treatment on the subgingival microbiota: A systematic review and meta-analysis. Orthodont Craniofac Res. 2018;21:175–85.

Reason: Intervention

227. Giraldo VM, Duque A, Aristizabal AG, Hernández RDM. Prevalence of peri-implant disease according to periodontal probing depth and bleeding on probing: A systematic review and meta-analysis. Int J Oral Maxillofac Implants. 2018;33:e89–105.

Reason: Intervention

228. Li T, Zhai X, Song F, Zhu H. Selective versus non-selective removal for dental caries: a systematic review and meta-analysis. Acta Odontol Scand. 2018;76:135–40.

Reason: Intervention

229. Roca-Millan E, González-Navarro B, Del Mar Sabater-Recolons M, Marí-Roig A, Jané-Salas E, López-López J. Periodontal treatment on patients with cardiovascular disease: Systematic review and meta-analysis. Med Oral Patol Oral Cir Bucal. 2018;23:e681–90.

Reason: Intervention

230. Lim G, Lin G-H, Monje A, Chan H-L, Wang H-L. Wound healing complications following guided bone regeneration for ridge augmentation: A systematic review and meta-analysis. Int J Oral Maxillofac Implants. 2018;33:41–50.

Reason: Intervention

231. Salvi GE, Monje A, Tomasi C. Long-term biological complications of dental implants placed either in pristine or in augmented sites: A systematic review and meta-analysis. Clin Oral Implants Res. 2018;29:294–310.

Reason: Intervention

232. Antonoglou GN, Stavropoulos A, Samara MD, Ioannidis A, Benic GI, Papageorgiou SN, et al. Clinical performance of dental implants following sinus floor augmentation: A systematic review and meta-analysis of clinical trials with at least 3 years of follow-up. Int J Oral Maxillofac Implants. 2018;33:e45–65.

Reason: Intervention

233. Sun C, Sun J, Tan M, Hu B, Gao X, Song J. Pain after root canal treatment with different instruments: A systematic review and meta-analysis. Oral Dis. 2018;24:908–19.

Reason: Intervention

234. Canellas JVDS, Medeiros PJD, Figueredo CMDS, Fischer RG, Ritto FG. Platelet-rich fibrin in oral surgical procedures: a systematic review and meta-analysis. Int J Oral Maxillofac Surg. 2019;48:395–414.

Reason: Intervention

235. Howe M-S, Keys W, Richards D. Long-term (10-year) dental implant survival: A systematic review and sensitivity meta-analysis. J Dent. 2019;84:9–21.

Reason: Intervention

236. Al-Moraissi E, Elsharkawy A, Al-Tairi N, Farhan A, Abotaleb B, Alsharaee Y, et al. What surgical approach has the lowest risk of the lower lid complications in the treatment of orbital floor and periorbital fractures? A frequentist network meta-analysis. J Cranio-Maxillofac Surg. 2018;46:2164–75.

Reason: Intervention and other study design

237. Papageorgiou SN, Eliades T, Hämmerle CHF. Frequency of infraposition and missing contact points in implant-supported restorations within natural dentitions over time: A systematic review with meta-analysis. Clin Oral Implants Res. 2018;29:309–25.

Reason: Intervention

238. Pereira MC, Mello FW, Ribeiro DM, Porporatti AL, da Costa S, Flores-Mir C, et al. Prevalence of reported percutaneous injuries on dentists: A meta-analysis. J Dent. 2018;76:9–18.

Reason: Not related to topic

239. Sardana D, Zhang J, Ekambaram M, Yang Y, McGrath CP, Yiu CKY. Effectiveness of professional fluorides against enamel white spot lesions during fixed orthodontic treatment: A systematic review and meta-analysis. J Dent. 2019;82:1–10.

Reason: Intervention

240. Cabanillas-Balsera D, Martín-González J, Montero-Miralles P, Sánchez-Domínguez B, Jiménez-Sánchez MC, Segura-Egea JJ. Association between diabetes and nonretention of root filled teeth: a systematic review and meta-analysis. Int Endod J. 2019;52:297–306.

Reason: Intervention

241. Wusiman P, Nie B, Li WD, Moming A. Management of mandibular angle fractures using 3- dimensional or standard miniplates: A systematic review and meta-analysis. J Cranio-Maxillofac Surg. 2019;47:622–8.

Reason: Intervention

242. Yang W-F, Wong MCM, Thomson PJ, Li K-Y, Su Y-X. The prognostic role of PD-L1 expression for survival in head and neck squamous cell carcinoma: A systematic review and meta-analysis. Oral Oncol. 2018;86:81–90.

Reason: Not related to topic

243. Lian M, Zhao K, Feng Y, Yao Q. Prognosis of combining remaining teeth and implants in double-crown-retained removable dental prostheses: A systematic review and meta-analysis. Int J Oral Maxillofac Implants. 2018;33:281–97.

Reason: Intervention

244. Nascimento RRD, Masterson D, Mattos CT, Vilella ODV. Facial growth direction after surgical intervention to relieve mouth breathing: A systematic review and meta-analysis. J Orofacial Orthop. 2018;79:412–26.

Reason: Intervention

245. Rakic M, Galindo-Moreno P, Monje A, Radovanovic S, Wang H-L, Cochran D, et al. How frequent does peri-implantitis occur? A systematic review and meta-analysis. Clin Oral Invest. 2018;22:1805–16.

Reason: Intervention

246. Radi IA-W, Ibrahim W, Iskandar SMS, AbdelNabi N. Prognosis of dental implants in patients with low bone density: A systematic review and meta-analysis. J Prosthet Dent. 2018;120:668–77.

Reason: Intervention

247. ElHoussiney AG, Zhang H, Song J, Ji P, Wang L, Yang S. Influence of implant location on the clinical outcomes of implant abutments: a systematic review and meta-analysis. Clin Cosmet Invest Dent. 2018;10:19–35.

Reason: Intervention

248. de Melo Alencar C, de Paula BLF, Guanipa Ortiz MI, Baraúna Magno M, Martins Silva C, Cople Maia L. Clinical efficacy of nano-hydroxyapatite in dentin hypersensitivity: A systematic review and meta-analysis. J Dent. 2019;82:11–21.

Reason: Intervention

249. Lo Y-F, Crispin A, Kesslerc A, Hickeld R, Kühnisch J. What is an appropriate etching time for sealant application on permanent molars? Results from a meta-analysis. J Adhes Dent. 2019;21:487–95.

Reason: Intervention and other study design

250. Shamszadeh S, Shirvani A, Eghbal MJ, Asgary S. Efficacy of Corticosteroids on Postoperative Endodontic Pain: A Systematic Review and Meta-analysis. J Endod. 2018;44:1057–65.

Reason: Intervention

251. Wusiman P, Taxifulati D, Weidong L, Moming A. Three-dimensional versus standard miniplate, lag screws versus miniplates, locking plate versus non-locking miniplates: Management of mandibular fractures, a systematic review and meta-analysis. J Dent Sci. 2019;14:66–80.

Reason: Intervention

252. Rodríguez Sánchez F, Rodríguez Andrés C, Arteagoitia I. Which antibiotic regimen prevents implant failure or infection after dental implant surgery? A systematic review and meta-analysis. J Cranio-Maxillofac Surg. 2018;46:722–36.

Reason: Intervention

253. Sanz-Sánchez I, Carrillo de Albornoz A, Figuero E, Schwarz F, Jung R, Sanz M, et al. Effects of lateral bone augmentation procedures on peri-implant health or disease: A systematic review and meta-analysis. Clin Oral Implants Res. 2018;29:18–31.

Reason: Intervention

254. Suneelkumar C, Subha A, Gogala D. Effect of Preoperative Corticosteroids in Patients with Symptomatic Pulpitis on Postoperative Pain after Single-visit Root Canal Treatment: A Systematic Review and Meta-analysis. J Endod. 2018;44:1347–54.

Reason: Intervention

255. Araujo RZ, Santiago Júnior JF, Cardoso CL, Benites Condezo AF, Moreira Júnior R, Curi MM. Clinical outcomes of pterygoid implants: Systematic review and meta-analysis. J Cranio-Maxillofac Surg. 2019;47:651–60.

Reason: Intervention

256. Owattanapanich D, Ungprasert P, Owattanapanich W. Efficacy of local tranexamic acid treatment for prevention of bleeding after dental procedures: A systematic review and meta-analysis. J Dent Sci. 2019;14:21–6.

Reason: Intervention

257. Jodeh DS, Buller M, Rottgers SA. The Impact of Presurgical Infant Orthopedics on Oronasal Fistula Rates Following Cleft Repair: A Meta-Analysis. Cleft Palate-Craniofac J. 2019;56:576–85.

Reason: Intervention

258. Dutra KL, Haas LF, Zimmermann GS, Melo G, Minamisako MC, Flores-Mir C, et al. Prevalence of radiographic findings on jaws exposed to antiresorptive therapy: A meta-analysis. Dentomaxillofac Radiol. 2019;48.

Reason: Intervention

259. El-Ashmawi NA, ElKordy SA, Salah Fayed MM, El-Beialy A, Attia KH. Effectiveness of Gingivoperiosteoplasty on Alveolar Bone Reconstruction and Facial Growth in Patients With Cleft Lip and Palate: A Systematic Review and Meta-Analysis. Cleft Palate-Craniofac J. 2019;56:438–53.

Reason: Intervention

260. Wang X, Shu X, Zhang Y, Yang B, Jian Y, Zhao K. Evaluation of fiber posts vs metal posts for restoring severely damaged endodontically treated teeth: A systematic review and meta-analysis. Quintessence Int. 2019;50:8–20.

Reason: Intervention

261. Xiao J, Alkhers N, Kopycka-Kedzierawski DT, Billings RJ, Wu TT, Castillo DA, et al. Prenatal Oral Health Care and Early Childhood Caries Prevention: A Systematic Review and Meta-Analysis. Caries Res. 2019;53:411–21.

Reason: Intervention

262. Pauletto P, Ruales-Carrera E, Gonçalves TMSV, Philippi AG, Donos N, Mezzomo LA. Fixed and removable full-arch restorations supported by short (≤ 8 mm) dental implants in the mandible: A systematic review and meta-analysis. Int J Oral Maxillofac Implants. 2019;34:873–885a.

Reason: Intervention

263. Karna H, Gonzalez J, Radia HS, Sedghizadeh PP, Enciso R. Risk-reductive dental strategies for medication related osteonecrosis of the jaw among cancer patients: A systematic review with meta-analyses. Oral Oncol. 2018;85:15–23.

Reason: Intervention

264. Abdulrab S, Rodrigues JC, Al-maweri SA, Halboub E, Alqutaibi AY, Alhadainy H. Effect of Apical Patency on Postoperative Pain: A Meta-analysis. J Endod. 2018;44:1467–73.

Reason: Intervention

265. Deschamps-Lenhardt S, Martin-Cabezas R, Hannedouche T, Huck O. Association between periodontitis and chronic kidney disease: Systematic review and meta-analysis. Oral Dis. 2019;25:385–402.

Reason: Intervention

266. Ramaglia L, Guida A, Iorio-Siciliano V, Cuozzo A, Blasi A, Sculean A. Stage-specific therapeutic strategies of medication-related osteonecrosis of the jaws: a systematic review and meta-analysis of the drug suspension protocol. Clin Oral Invest. 2018;22:597–615.

Reason: Intervention

267. Porporatti AL, Costa YM, Réus JC, Stuginski-Barbosa J, Conti PCR, Velly AM, et al. Placebo and nocebo response magnitude on temporomandibular disorder-related pain: A systematic review and meta-analysis. J Oral Rehabil. 2019;46:862–82.

Reason: Intervention

268. Hou X-M, Su Z, Hou B-X. Post endodontic pain following single-visit root canal preparation with rotary vs reciprocating instruments: A meta-analysis of randomized clinical trials. BMC Oral Health. 2017;17:1.

Reason: Intervention

269. Del Fabbro M, Bucchi C, Lolato A, Corbella S, Testori T, Taschieri S. Healing of Postextraction Sockets Preserved With Autologous Platelet Concentrates. A Systematic Review and Meta-Analysis. J Oral Maxillofac Surg. 2017;75:1601–15.

Reason: Intervention

270. Segura-Egea JJ, Martín-González J, Cabanillas-Balsera D, Fouad AF, Velasco-Ortega E, López-López J. Association between diabetes and the prevalence of radiolucent periapical lesions in root-filled teeth: systematic review and meta-analysis. Clin Oral Invest. 2016;20:1133–41.

Reason: Intervention

271. Canellas JVDS, Ritto FG, Medeiros PJD. Evaluation of postoperative complications after mandibular third molar surgery with the use of platelet-rich fibrin: a systematic review and meta-analysis. Int J Oral Maxillofac Surg. 2017;46:1138–46.

Reason: Intervention

272. Rodríguez Sánchez F, Rodríguez Andrés C, Arteagoitia Calvo I. Does Chlorhexidine Prevent Alveolar Osteitis After Third Molar Extractions? Systematic Review and Meta-Analysis. J Oral Maxillofac Surg. 2017;75:901–14.

Reason: Intervention

273. Hong N, Yang H, Li J, Wu S, Li Y. Effect of preparation designs on the prognosis of porcelain laminate veneers: A systematic review and meta-analysis. Oper Dent. 2017;42:E197–213.

Reason: Intervention

274. Doornewaard R, Christiaens V, De Bruyn H, Jacobsson M, Cosyn J, Vervaeke S, et al. Long-Term Effect of Surface Roughness and Patients’ Factors on Crestal Bone Loss at Dental Implants. A Systematic Review and Meta-Analysis. Clin Implant Dent Relat Res. 2017;19:372–99.

Reason: Intervention

275. Clé-Ovejero A, Sánchez-Torres A, Camps-Font O, Gay-Escoda C, Figueiredo R, Valmaseda-Castellón E. Does 3-dimensional imaging of the third molar reduce the risk of experiencing inferior alveolar nerve injury owing to extraction?: A meta-analysis. J Am Dent Assoc. 2017;148:575–83.

Reason: Intervention and other study design

276. Altmann ASP, Collares FM, Leitune VCB, Samuel SMW. The effect of antimicrobial agents on bond strength of orthodontic adhesives: A meta-analysis of in vitro studies. Orthodont Craniofac Res. 2016;19:1–9.

Reason: Intervention

277. Zhang B, Mo Z, Du W, Wang Y, Liu L, Wei Y. Intensity-modulated radiation therapy versus 2D-RT or 3D-CRT for the treatment of nasopharyngeal carcinoma: A systematic review and meta-analysis. Oral Oncol. 2015;51:1041–6.

Reason: Not related to topic and intervention

278. Papageorgiou SN, Dimitraki D, Kotsanos N, Bekes K, van Waes H. Performance of pit and fissure sealants according to tooth characteristics: A systematic review and meta-analysis. J Dent. 2017;66:8–17.

Reason: Intervention

279. Al-Moraissi EA, Thaller SR, Ellis E. Subciliary vs. transconjunctival approach for the management of orbital floor and periorbital fractures: A systematic review and meta-analysis. J Cranio-Maxillofac Surg. 2017;45:1647–54.

Reason: Intervention

280. Cervera-Espert J, Pérez-Martínez S, Cervera-Ballester J, Peñarrocha-Oltra D, Peñarrocha-Diago M. Coronectomy of impacted mandibular third molars: A meta-analysis and systematic review of the literature. Med Oral Patol Oral Cir Bucal. 2016;21:e505–13.

Reason: Intervention

281. Okada EMP, Ribeiro LNS, Stuani MBS, Borsatto MC, Fidalgo TKDS, de Paula-Silva FWG, et al. Effects of chlorhexidine varnish on caries during orthodontic treatment: a systematic review and meta-analysis. Braz Oral Res. 2016;30.

Reason: Intervention

282. Wei J, Pei S, Zhu X. Comparison of (18)F-FDG PET/CT, MRI and SPECT in the diagnosis of local residual/recurrent nasopharyngeal carcinoma: A meta-analysis. Oral Oncol. 2016;52:11–7.

Reason: Not related to topic

283. Costa LA, Ribeiro CCC, Cantanhede LM, Santiago Júnior JF, de Mendonça MR, Pereira ALP. Treatments for intrusive luxation in permanent teeth: a systematic review and meta-analysis. Int J Oral Maxillofac Surg. 2017;46:214–29.

Reason: Intervention

284. Ata-Ali F, Ata-Ali J, Ferrer-Molina M, Cobo T, De Carlos F, Cobo J. Adverse effects of lingual and buccal orthodontic techniques: A systematic review and meta-analysis. Am J Orthod Dentofacial Orthop. 2016;149:820–9.

Reason: Intervention

285. Lee C-T, Huang Y-W, Zhu L, Weltman R. Prevalences of peri-implantitis and peri-implant mucositis: systematic review and meta-analysis. J Dent. 2017;62:1–12.

Reason: Intervention

286. Verweij JP, Houppermans PNWJ, Gooris P, Mensink G, van Merkesteyn JPR. Risk factors for common complications associated with bilateral sagittal split osteotomy: A literature review and meta-analysis. J Cranio-Maxillofac Surg. 2016;44:1170–80.

Reason: Intervention

287. Kung J, McDonagh M, Sedgley CM. Does articaine provide an advantage over lidocaine in patients with symptomatic irreversible pulpitis? A systematic review and meta-analysis. J Endod. 2015;41:1784–94.

Reason: Intervention

288. Moreno-Drada JA, García-Perdomo HA. Effectiveness of Antimicrobial Prophylaxis in Preventing the Spread of Infection as a Result of Oral Procedures: A Systematic Review and Meta-Analysis. J Oral Maxillofac Surg. 2016;74:1313–21.

Reason: Intervention

289. Steenen SA, van Wijk AJ, Becking AG. Bad splits in bilateral sagittal split osteotomy: systematic review and meta-analysis of reported risk factors. Int J Oral Maxillofac Surg. 2016;45:971–9.

Reason: Intervention

290. Gruner D, Paris S, Schwendicke F. Probiotics for managing caries and periodontitis: Systematic review and meta-analysis. J Dent. 2016;48:16–25.

Reason: Intervention

291. Al-Dajani M. Incidence, risk factors, and complications of schneiderian membrane perforation in sinus lift surgery: A meta-analysis. Implant Dent. 2016;25:409–15.

Reason: Intervention

292. Al-Moraissi EA, Perez D, Ellis E. Do patients with malocclusion have a higher prevalence of temporomandibular disorders than controls both before and after orthognathic surgery? A systematic review and meta-analysis. J Cranio-Maxillofac Surg. 2017;45:1716–23.

Reason: Intervention

293. Xu J-L, Xia R, Sun Z-H, Sun L, Min X, Liu C, et al. Effects of honey use on the management of radio/chemotherapy-induced mucositis: a meta-analysis of randomized controlled trials. Int J Oral Maxillofac Surg. 2016;45:1618–25.

Reason: Intervention

294. Toti P, Marchionni S, Menchini-Fabris GB, Marconcini S, Covani U, Barone A. Surgical techniques used in the rehabilitation of partially edentulous patients with atrophic posterior mandibles: A systematic review and meta-analysis of randomized controlled clinical trials. J Cranio-Maxillofac Surg. 2017;45:1236–45.

Reason: Intervention

295. Cardona A, Balouch A, Abdul MM, Sedghizadeh PP, Enciso R. Efficacy of chlorhexidine for the prevention and treatment of oral mucositis in cancer patients: a systematic review with meta-analyses. J Oral Pathol Med. 2017;46:680–8.

Reason: Intervention

296. Machado LA, do Nascimento RR, Ferreira DMTP, Mattos CT, Vilella OV. Long-term prognosis of tooth autotransplantation: a systematic review and meta-analysis. Int J Oral Maxillofac Surg. 2016;45:610–7.

Reason: Intervention

297. de Almeida VL, Lima IFP, Ziegelmann PK, Paranhos LR, de Matos FR. Impact of highly active antiretroviral therapy on the prevalence of oral lesions in HIV-positive patients: a systematic review and meta-analysis. Int J Oral Maxillofac Surg. 2017;46:1497–504.

Reason: Intervention

298. Marques AEM, Elias ST, Porporatti AL, Castilho RM, Squarize CH, De Luca Canto G, et al. mTOR pathway protein immunoexpression as a prognostic factor for survival in head and neck cancer patients: A systematic review and meta-analysis. J Oral Pathol Med. 2016;45:319–28.

Reason: Not related to topic

299. Armond ACV, Jalles Milani LM, de Fátima Barbosa Fonseca J, de Castro Martins C, Moreira Falci SG. Does the use of intra-alveolar chlorhexidine gel reduces the rate of alveolar osteitis, pain, edema and trismus after the extraction of lower third molars? A meta analysis. J Oral Maxillofacial Surg Med Pathol. 2017;29:491–8.

Reason: Intervention

300. Al-Hamed FS, Tawfik MA-M, Abdelfadil E, Al-Saleh MAQ. Efficacy of Platelet-Rich Fibrin After Mandibular Third Molar Extraction: A Systematic Review and Meta-Analysis. J Oral Maxillofac Surg. 2017;75:1124–35.

Reason: Intervention

301. Geminiani A, Tsigarida A, Chochlidakis K, Papaspyridakos PV, Feng C, Ercoli C. A meta-analysis of complications during sinus augmentation procedure. Quintessence Int. 2017;48:231–40.

Reason: Intervention

302. Teshome A. The efficacy of chlorhexidine gel in the prevention of alveolar osteitis after mandibular third molar extraction: A systematic review and meta-analysis. BMC Oral Health. 2017;17.

Reason: Intervention

303. Bohner LOL, Mukai E, Oderich E, Porporatti AL, Pacheco-Pereira C, Tortamano P, et al. Comparative analysis of imaging techniques for diagnostic accuracy of peri-implant bone defects: a meta-analysis. Oral Surg Oral Med Oral Pathol Oral Radiol. 2017;124:432-440.e5.

Reason: Intervention

304. Pang P, Li R-W, Shi J-P, Xu Z-F, Duan W-Y, Liu F-Y, et al. A comparison of mandible preservation method and mandibulotomy approach in oral and oropharyngeal cancer: A meta-analysis. Oral Oncol. 2016;63:52–60.

Reason: Intervention

305. García-Guerrero C, Guauque SQ, Molano N, Pineda GA, Nino-Barrera JL, Marín-Zuluaga DJ. Predictors of clinical outcomes in endodontic microsurgery: a systematic review and meta-analysis. G Ital Endodonzia. 2017;31:2–13.

Reason: Intervention

306. Joshi A, Goel M, Thorat A. Identifying the risk factors causing iatrogenic mandibular fractures associated with exodontia: a systemic meta-analysis of 200 cases from 1953 to 2015. Oral Maxillofac Surg. 2016;20:391–6.

Reason: Intervention

307. Erratum: Bioadhesive chlorhexidine gel for reduction of alveolar osteitis incidence: Systematic review and meta-analysis of randomized controlled trials (Dental Hypotheses (2014) 5:2 (35-40) DOI: 10.4103/2155-8213.133420). Dent Hypotheses. 2017;8:52.

Reason: Other study design

308. Karl M, Albrektsson T. Clinical performance of dental implants with a moderately rough (TiUnite) surface: A meta-analysis of prospective clinical studies. Int J Oral Maxillofac Implants. 2017;32:717–34.

Reason: Intervention

309. Di Girolamo M, Calcaterra R, Di Gianfilippo R, Arcuri C, Baggi L. Bone level changes around platform switching and platform matching implants: A systematic review with meta-analysis. ORAL Implantol. 2016;9:1–10.

Reason: Intervention

310. Szturz P, Budíková M, Vermorken JB, Horová I, Gál B, Raymond E, et al. Prognostic value of c-MET in head and neck cancer: A systematic review and meta-analysis of aggregate data. Oral Oncol. 2017;74:68–76.

Reason: Not related to topic

311. Deng Y, Zhu X, Zheng D, Yan P, Jiang H. Laser use in direct pulp capping: A meta-analysis. J Am Dent Assoc. 2016;147:935–42.

Reason: Intervention

312. Lin H-K, Fang C-E, Huang M-S, Cheng H-C, Huang T-W, Chang H-T, et al. Effect of maternal use of chewing gums containing xylitol on transmission of mutans streptococci in children: A meta-analysis of randomized controlled trials. Int J Paediatr Dent. 2016;26:35–44.

Reason: Intervention

313. Smith EA, Marshall JG, Selph SS, Barker DR, Sedgley CM. Nonsteroidal Anti-inflammatory Drugs for Managing Postoperative Endodontic Pain in Patients Who Present with Preoperative Pain: A Systematic Review and Meta-analysis. J Endod. 2017;43:7–15.

Reason: Intervention

314. Su Z, Duan Z, Pan W, Wu C, Jia Y, Han B, et al. Predicting extracapsular spread of head and neck cancers using different imaging techniques: A systematic review and meta-analysis. Int J Oral Maxillofac Surg. 2016;45:413–21.

Reason: Not related to topic

315. Chen H, Zhou N, Huang X, Song S. Comparison of morbidity after reconstruction of tongue defects with an anterolateral thigh cutaneous flap compared with a radial forearm free-flap: a meta-analysis. Br J Oral Maxillofac Surg. 2016;54:1095–101.

Reason: Intervention and other study design

316. De Geus JL, Wambier LM, Kossatz S, Loguercio AD, Reis A. At-home vs in-office bleaching: A systematic review and meta-analysis. Oper Dent. 2016;41:341–56.

Reason: Intervention

317. Wusiman P, Yarbag A, Wurouzi G, Mijiti A, Moming A. Three dimensional versus standard miniplate fixation in management of mandibular fractures: A systematic review and meta-analysis. J Cranio-Maxillofac Surg. 2016;44:1646–54.

Reason: Intervention

318. Jean S, Dionne P-L, Bouchard C, Giasson L, Turgeon AF. Perioperative Systemic Corticosteroids in Orthognathic Surgery: A Systematic Review and Meta-Analysis. J Oral Maxillofac Surg. 2017;75:2638–49.

Reason: Intervention

319. Wong AW-Y, Zhang S, Li SK-Y, Zhang C, Chu C-H. Clinical studies on core-carrier obturation: A systematic review and meta-analysis. BMC Oral Health. 2017;17.

Reason: Intervention

320. Arteagoitia M-I, Barbier L, Santamaría J, Santamaría G, Ramos E. Efficacy of amoxicillin and amoxicillin/clavulanic acid in the prevention of infection and dry socket after third molar extraction. A systematic review and meta-analysis. Med Oral Patol Oral Cir Bucal. 2016;21:e494–504.

Reason: Intervention

321. Papageorgiou SN, Dimitraki D, Coolidge T, Kotsanos N. Publication bias and small-study effects in pediatric dentistry meta-analyses. J Evid-Based Dent Pract. 2015;15:8–24.

Reason: Other study design

322. de Amorim RG, Leal SC, Frencken JE. Survival of atraumatic restorative treatment (ART) sealants and restorations: A meta-analysis. Clin Oral Invest. 2012;16:429–41.

Reason: Intervention

323. Kyzas PA, Saeed A, Tabbenor O. The treatment of mandibular condyle fractures: A meta-analysis. J Cranio-Maxillofac Surg. 2012;40:e438–52.

Reason: Intervention and other study design

324. De Luca Canto G, Singh V, Major MP, Witmans M, El-Hakim H, Major PW, et al. Diagnostic capability of questionnaires and clinical examinations to assess sleep-disordered breathing in children: A systematic review and meta-analysis. J Am Dent Assoc. 2014;145:165–78.

Reason: Not related to topic

325. Figueiredo FED, Martins-Filho PRS, Faria-E-Silva AL. Do metal post-retained restorations result in more root fractures than fiber post-retained restorations? A systematic review and meta-analysis. J Endod. 2015;41:309–16.

Reason: Intervention

326. Monje A, Chan H-L, Suarez F, Galindo-Moreno P, Wang H-L. Marginal bone loss around tilted implants in comparison to straight implants: A meta-analysis. Int J Oral Maxillofac Implants. 2012;27:1576–83.

Reason: Intervention

327. Foresta E, Torroni A, Di Nardo F, De Waure C, Poscia A, Gasparini G, et al. Pleomorphic adenoma and benign parotid tumors: Extracapsular dissection vs superficial parotidectomy - Review of literature and meta-analysis. Oral Surg Oral Med Oral Pathol Oral Radiol. 2014;117:663–76.

Reason: Intervention

328. Rakhshan V. Meta-analysis and systematic review of factors biasing the observed prevalence of congenitally missing teeth in permanent dentition excluding third molars. Prog Orthod. 2013;14:1–12.

Reason: Other study design

329. Panitvisai P, Parunnit P, Sathorn C, Messer HH. Impact of a Retained Instrument on Treatment Outcome: A Systematic Review and Meta-analysis. J Endod. 2010;36:775–80.

Reason: Intervention

330. Faot F, Nascimento GG, Bielemann AM, Campão TD, Leite FRM, Quirynen M. Can peri-implant crevicular fluid assist in the diagnosis of peri-implantitis? A systematic review and meta-analysis. J Periodontol. 2015;86:631–45.

Reason: Intervention

331. Papadopoulos MA, Gkiaouris I. A critical evaluation of meta-analyses in orthodontics. Am J Orthod Dentofacial Orthop. 2007;131:589-599.e7.

Reason: Other study design

332. Jiang Y-M, Zhu X -d., Qu S. Incidence of osteoradionecrosis in patients who have undergone dental extraction prior to radiotherapy: A systematic review and meta-analysis. J Oral Maxillofacial Surg Med Pathol. 2014;26:269–75.

Reason: Intervention

333. Peng L, Ye L, Tan H, Zhou X. Outcome of Root Canal Obturation by Warm Gutta-Percha versus Cold Lateral Condensation: A Meta-analysis. J Endod. 2007;33:106–9.

Reason: Intervention

334. Chrcanovic BR. Surgical versus non-surgical treatment of mandibular condylar fractures: A meta-analysis. Int J Oral Maxillofac Surg. 2015;44:158–79.

Reason: Intervention

335. Motamedi MRK, Khazaei S. Bioadhesive chlorhexidine gel for reduction of alveolar osteitis incidence: Systematic review and meta-analysis of randomized controlled trials. Dent Hypotheses. 2014;5:35–40.

Reason: Intervention

336. Hoshijima H, Kuratani N, Takeuchi R, Shiga T, Masaki E, Doi K, et al. Effects of oral hygiene using chlorhexidine on preventing ventilator-associated pneumonia in critical-care settings: A meta-analysis of randomized controlled trials. J Dent Sci. 2013;8:348–57.

Reason: Intervention and other study design

337. Kalsi R, Vandana KL, Prakash S. Effect of local drug delivery in chronic periodontitis patients: A meta-analysis. J Ind Soc Periodontol. 2011;15:304–9.

Reason: Intervention

338. Nixdorf DR, Moana-Filho EJ, Law AS, McGuire LA, Hodges JS, John MT. Frequency of Persistent Tooth Pain after Root Canal Therapy: A Systematic Review and Meta-Analysis. J Endod. 2010;36:224–30.

Reason: Intervention

339. Peng L, Ye L, Guo X, Tan H, Zhou X, Wang C, et al. Evaluation of formocresol versus ferric sulphate primary molar pulpotomy: A systematic review and meta-analysis. Int Endod J. 2007;40:751–7.

Reason: Intervention

340. Zhou C, Sun B. The prognostic role of the cancer stem cell marker aldehyde dehydrogenase 1 in head and neck squamous cell carcinomas: A meta-analysis. Oral Oncol. 2014;50:1144–8.

Reason: Not related to topic and other study design

341. Strietzel FP, Reichart PA, Kale A, Kulkarni M, Wegner B, Küchler I. Smoking interferes with the prognosis of dental implant treatment: A systematic review and meta-analysis. J Clin Periodontol. 2007;34:523–44.

Reason: Intervention

342. Setzer FC, Kohli MR, Shah SB, Karabucak B, Kim S. Outcome of endodontic surgery: A meta-analysis of the literature - Part 2: Comparison of endodontic microsurgical techniques with and without the use of higher magnification. J Endod. 2012;38:1–10.

Reason: Intervention

343. Papageorgiou SN, Papadelli AP, Koidis PT, Petridis HP. The effect of prosthetic margin location on caries susceptibility. A systematic review and meta-analysis. Brit Dent J. 2013;214:617–24.

Reason: Intervention

344. Kolokitha O-E, Kaklamanos EG, Papadopoulos MA. Prevalence of nickel hypersensitivity in orthodontic patients: A meta-analysis. Am J Orthod Dentofacial Orthop. 2008;134:722.e1-722.e12.

Reason: Other study design

345. Saulle R, Semyonov L, Mannocci A, Careri A, Saburri F, Ottolenghi L, et al. Human papillomavirus and cancerous diseases of the head and neck: A systematic review and meta-analysis. Oral Dis. 2015;21:417–31.

Reason: Not related to topic

346. Tan C, Liu Y, Li W, Liu J, Chen L. Transcutaneous neuromuscular electrical stimulation can improve swallowing function in patients with dysphagia caused by non-stroke diseases: A meta-analysis. J Oral Rehabil. 2013;40:472–80.

Reason: Intervention and other study design

347. Sood AJ, Fox NF, O’Connell BP, Lovelace TL, Nguyen SA, Sharma AK, et al. Salivary gland transfer to prevent radiation-induced xerostomia: A systematic review and meta-analysis. Oral Oncol. 2014;50:77–83.

Reason: Intervention

348. Al-Moraissi EÀ, El-Sharkawy TM, El-Ghareeb TI, Chrcanovic BR. Three-dimensional versus standard miniplate fixation in the management of mandibular angle fractures: A systematic review and meta-analysis. Int J Oral Maxillofac Surg. 2014;43:708–16.

Reason: Intervention

349. Zhao Z, Ge J, Sun Y, Tian L, Lu J, Liu M, et al. Is E-cadherin immunoexpression a prognostic factor for head and neck squamous cell carcinoma (HNSCC)? A systematic review and meta-analysis. Oral Oncol. 2012;48:761–7.

Reason: Not related to topic and intervention

350. Xu J-L, Sun L, Liu C, Sun Z-H, Min X, Xia R. Effect of oral contraceptive use on the incidence of dry socket in females following impacted mandibular third molar extraction: a meta-analysis. Int J Oral Maxillofac Surg. 2015;44:1160–5.

Reason: Intervention

351. Gaudin E, Seidel L, Bacevic M, Rompen E, Lambert F. Occurrence and risk indicators of medication-related osteonecrosis of the jaw after dental extraction: A systematic review and meta-analysis. J Clin Periodontol. 2015;42:922–32.

Reason: Intervention

352. Wang X, San Y, Sun J, Zhou H, Li X, Zhang Z, et al. Validation of the Chinese version of ID-Migraine in medical students and systematic review with meta-analysis concerning its diagnostic accuracy. J Oral Facial Pain Headache. 2015;29:265–78.

Reason: Not related to topic

353. Jamali Z, Aminabadi NA, Attaran R, Pournagiazar F, Oskouei SG, Ahmadpour F. MicroRNAs as prognostic molecular signatures in human head and neck squamous cell carcinoma: A systematic review and meta-analysis. Oral Oncol. 2015;51:321–31.

Reason: Intervention

354. Monje A, Alcoforado G, Padial-Molina M, Suarez F, Lin G-H, Wang H-L. Generalized aggressive periodontitis as a risk factor for dental implant failure: A systematic review and meta-analysis. J Periodontol. 2014;85:1398–407.

Reason: Intervention

355. Sun R, Tang X, Yang Y, Zhang C. 18FDG-PET/CT for the detection of regional nodal metastasis in patients with head and neck cancer: A meta-analysis. Oral Oncol. 2015;51:314–20.

Reason: Not related to topic and other study design

356. Atieh MA, Alsabeeha NHM, Faggion Jr. CM, Duncan WJ. The frequency of peri-implant diseases: A systematic review and meta-Analysis. J Periodontol. 2013;84:1586–98.

Reason: Intervention

357. Tsesis I, Faivishevsky V, Fuss Z, Zukerman O. Flare-ups after Endodontic Treatment: A Meta-analysis of Literature. J Endod. 2008;34:1177–81.

Reason: Intervention and other study design

358. Chrcanovic BR. Locking versus non-locking plate fixation in the management of mandibular fractures: A meta-analysis. Int J Oral Maxillofac Surg. 2014;43:1243–50.

Reason: Intervention and other study design

359. Bozini T, Petridis HP, Tzanas K, Garefis P. A meta-analysis of prosthodontic complication rates of implant-supported fixed dental prostheses in edentulous patients after an observation period of at least 5 years. Int J Oral Maxillofac Implants. 2011;26:304–18.

Reason: Intervention

360. Sunnak R, Johal A, Fleming PS. Is orthodontics prior to 11 years of age evidence-based? A systematic review and meta-analysis. J Dent. 2015;43:477–86.

Reason: Intervention

361. Laleman I, Detailleur V, Slot DE, Slomka V, Quirynen M, Teughels W. Probiotics reduce mutans streptococci counts in humans: A systematic review and meta-analysis. Clin Oral Invest. 2014;18:1539–52.

Reason: Intervention

362. Al-Moraissi EA, Ellis E. Surgical management of anterior mandibular fractures: A systematic review and meta-analysis. J Oral Maxillofac Surg. 2014;72:2507.e1-2507.e11.

Reason: Intervention

363. Beyari M-M, Strain D, Li C-S, Lamfon H-A. Conflict of interest reporting in dentistry meta-analyses: A systematic review. J Clini Exp Dent. 2014;6:e280–5.

Reason: Other study design and not related to topic

364. Liu W, Yin W, Zhang R, Li J, Zheng Y. Diagnostic value of panoramic radiography in predicting inferior alveolar nerve injury after mandibular third molar extraction: A meta-analysis. Aust Dent J. 2015;60:233–9.

Reason: Intervention and other study design

365. Ata-Ali J, Ata-Ali F. Do antibiotics decrease implant failure and postoperative infections? A systematic review and meta-analysis. Int J Oral Maxillofac Surg. 2014;43:68–74.

Reason: Intervention

366. Ramanauskaite A, Baseviciene N, Wang H-L, Tözüm TF. Effect of history of periodontitis on implant success: Meta-analysis and systematic review. Implant Dent. 2014;23:687–96.

Reason: Intervention

367. Al-Moraissi EA, Ellis E. What method for management of unilateral mandibular angle fractures has the lowest rate of postoperative complications? a systematic review and meta-analysis. J Oral Maxillofac Surg. 2014;72:2197–211.

Reason: Intervention

368. Van Rijkom HM, Truin GJ, Van ’t Hof MA. A Meta-Analysis of Clinical Studies on the Caries-Inhibiting Effect of Fluoride Gel Treatment. Caries Res. 1998;32:83–92.

Reason: Intervention

369. Kim M-R, Graber TM, Viana MA. Orthodontics and temporomandibular disorder: A meta-analysis. Am J Orthod Dentofacial Orthop. 2002;121:438–46.

Reason: Intervention and other study design

370. Burke SP, Silveira AM, Goldsmith LJ, Yancey JM, Van Stewart A, Scarfe WC. A meta-analysis of mandibular intercanine width in treatment and postretention. Angle Orthod. 1998;68:53–60.

Reason: Intervention and other study design

371. Kojima K, Inamoto K, Nagamatsu K, Hara A, Nakata K, Morita I, et al. Success rate of endodontic treatment of teeth with vital and nonvital pulps. a meta-analysis. Oral Surg Oral Med Oral Pathol Oral Radiol Endod. 2004;97:95–9.

Reason: Intervention and other study design

372. Daraqel B, Mheissen S, Li J, Khan H, Allan S, Zheng L. The effect of early versus delayed space closure on the rate of orthodontic tooth movement: a systematic review and meta-analysis. EUROPEAN JOURNAL OF ORTHODONTICS. 2023. https://doi.org/10.1093/ejo/cjad015.

Reason: Intervention

373. Li J, Zheng L, Daraqel B, Liu J, Hu Y. Treatment Outcome of Regenerative Endodontic Procedures for Necrotic Immature and Mature Permanent Teeth: A Systematic Review and Meta-Analysis Based on Randomised Controlled Trials. ORAL HEALTH & PREVENTIVE DENTISTRY. 2023;21:141–52.

Reason: Intervention

374. Chen J, Zhou Z, Liao Z, Zhang J, Yan J, Li W. Comparative Clinical Evaluation of Trapezoidal, Envelope, and Tunnel Type Coronally Advanced Flap in the Treatment of Gingival Recession: A Network Meta-Analysis of Randomized Clinical Trials. INTERNATIONAL JOURNAL OF PERIODONTICS & RESTORATIVE DENTISTRY. 2023;43:E61–71.

Reason: Intervention

375. Tang C, Du Q, Luo J, Peng L. Simultaneous placement of short implants (<= 8 mm) versus standard length implants (>= 10 mm) after sinus floor elevation in atrophic posterior maxillae: a systematic review and meta-analysis. INTERNATIONAL JOURNAL OF IMPLANT DENTISTRY. 2022;8.

Reason: Intervention

376. Keyhan S, Ramezanzade S, Yazdi R, Valipour M, Fallahi H, Shakiba M, et al. Prevalence of complications associated with polymer-based alloplastic materials in nasal dorsal augmentation: a systematic review and meta-analysis. MAXILLOFACIAL PLASTIC AND RECONSTRUCTIVE SURGERY. 2022;44.

Reason: Intervention

377. De Mendoza I, Setien-Olarra A, Garcia-De la Fuente A, Aguirre-Urizar J, Marichalar-Mendia X. Role of proinflammatory mutations in peri-implantitis: systematic review and meta-analysis. INTERNATIONAL JOURNAL OF IMPLANT DENTISTRY. 2022;8.

Reason: Intervention

378. Meng N, Liu Q, Dong Q, Gu J, Yang Y. Effects of probiotics on preventing caries in preschool children: a systematic review and meta-analysis. JOURNAL OF CLINICAL PEDIATRIC DENTISTRY. 2022;47:85–100.

Reason: Intervention

379. Abdulrahman B, Alanazi A, Alanazi A, Idrees F, Abuabah A, El Mansy I, et al. NATURAL THERAPEUTIC AGENTS IN THE TREATMENT OF RECURRENT APHTHOUS ULCER: A SYSTEMATIC REVIEW AND META-ANALYSIS. ANNALS OF DENTAL SPECIALTY. 2022;10:78–86.

Reason: Intervention

380. Faggion C, Menne M, Pandis N. Prediction intervals should be included in meta-analyses published in dentistry. EUROPEAN JOURNAL OF ORAL SCIENCES. 2021;129.

Reason: Other study design

381. Khater A, Al-hamed F, Safwat E, Hamouda M, Shehata M, Scarano A. EFFICACY OF HEMOSTATIC AGENTS IN ENDODONTIC SURGERY: A SYSTEMATIC REVIEW AND NETWORK META-ANALYSIS. JOURNAL OF EVIDENCE-BASED DENTAL PRACTICE. 2021;21.

Reason: Intervention

382. Stanek J, Riad A, Slezakova S, Azar B, Klugarova J, Pokorna A, et al. Bar fracture of implant-retained overdenture: Protocol of a systematic review and meta-analysis. JOURNAL OF OSSEOINTEGRATION. 2021;13:89–94.

Reason: Intervention

383. Casado B, Pellizzer E, Maior J, Lemos C, Vasconcelos B, Moraes S. Laser Influence on Dental Sensitivity Compared to Other Light Sources Used During In-office Dental Bleaching: Systematic Review and Meta-analysis. OPERATIVE DENTISTRY. 2020;45:589–97.

Reason: Intervention

384. Stacchi C, Troiano G, Berton F, Lombardi T, Rapani A, Englaro A, et al. Piezoelectric bone surgery for lateral sinus floor elevation compared with conventional rotary instruments: A systematic review, meta-analysis and trial sequential analysis. INTERNATIONAL JOURNAL OF ORAL IMPLANTOLOGY. 2020;13:109–21.

Reason: Intervention

385. Costa R, Moraes S, Lemos C, SoutoMaior J, Vasconcelos B, Pellizzer E. Effect of Analgesic Drugs on Tooth Sensitivity Induced by In-office Dental Bleaching: A Systematic Review and Meta-analysis. OPERATIVE DENTISTRY. 2020;45:E66–76.

Reason: Intervention

386. Duttenhoefer F, Fuessinger M, Beckmann Y, Schmelzeisen R, Groetz K, Boeker M. Dental implants in immunocompromised patients: a systematic review and meta-analysis. INTERNATIONAL JOURNAL OF IMPLANT DENTISTRY. 2019;5.

Reason: Intervention

387. Akram Z, Javed F, Vohra F. Effect of waterpipe smoking on peri-implant health: A systematic review and meta-analysis. JOURNAL OF INVESTIGATIVE AND CLINICAL DENTISTRY. 2019;10.

Reason: Intervention

388. SoutoMaior J, de Moraes S, Lemos C, Vasconcelos B, Montes M, Pellizzer E. Effectiveness of Light Sources on In-Office Dental Bleaching: A Systematic Review and Meta-Analyses. OPERATIVE DENTISTRY. 2019;44:E105–17.

Reason: Intervention

389. Dank A, Aartman I, Wismeijer D, Tahmaseb A. Effect of dental implant surface roughness in patients with a history of periodontal disease: a systematic review and meta-analysis. INTERNATIONAL JOURNAL OF IMPLANT DENTISTRY. 2019;5.

Reason: Intervention

390. Serindere G, Ozveren N. Gingival Enlargement in Patients who Have Undergone Renal Transplants: A Meta-Analysis. JOURNAL OF DENTISTRY INDONESIA. 2018;25:114–20.

Reason: Intervention and other study design

391. Jordi C, Mukaddam K, Lambrecht J, Kuhl S. Membrane perforation rate in lateral maxillary sinus floor augmentation using conventional rotating instruments and piezoelectric device-a meta-analysis. INTERNATIONAL JOURNAL OF IMPLANT DENTISTRY. 2018;4.

Reason: Intervention and other study design

392. Monje A, Aranda L, Diaz K, Alarcon M, Bagramian R, Wang H, et al. Impact of Maintenance Therapy for the Prevention of Peri-implant Diseases: A Systematic Review and Meta-analysis. JOURNAL OF DENTAL RESEARCH. 2016;95:372–9.

Reason: Intervention

393. Brouwer F, Askar H, Paris S, Schwendicke F. Detecting Secondary Caries Lesions: A Systematic Review and Meta-analysis. JOURNAL OF DENTAL RESEARCH. 2016;95:143–51.

Reason: Intervention

394. Nematullah A, Alabousi A, Blanas N, Douketis J, Sutherland S. Dental Surgery for Patients on Anticoagulant Therapy with Warfarin: A Systematic Review and Meta-analysis. JOURNAL OF THE CANADIAN DENTAL ASSOCIATION. 2009;75:41-41I.

Reason: Intervention

List of excluded studies after Fulltext assessment with reasons for exclusion

1. Trindade D, Carvalho R, Machado V, Chambrone L, Mendes JJ, Botelho J. Prevalence of periodontitis in dentate people between 2011 and 2020: A systematic review and meta-analysis of epidemiological studies. J Clin Periodontol. 2023;50:604–26.

Reason: Intervention

2. Atieh MA, Guirguis M, Alsabeeha NHM, Cannon RD. The diagnostic accuracy of saliva testing for SARS-CoV-2: A systematic review and meta-analysis. Oral Dis. 2022;28:2347–61.

Reason: Not related to topic

3. Martins JNR, Nole C, Ounsi HF, Parashos P, Plotino G, Ragnarsson MF, et al. Worldwide Assessment of the Mandibular First Molar Second Distal Root and Root Canal: A Cross-sectional Study with Meta-analysis. J Endod. 2022;48:223–33.

Reason: Other study design

4. Gioco G, Patini R, Rupe C, Di Petrillo A, Di Giovanni A, Isola G, et al. Oral psoriasis and temporo-mandibular disorders: A systematic review and meta-analysis. Dent Cadmos. 2022;90:100–7.

Reason: Other language than english

5. Zhang W, Zou B, Liu J, Yuan D. Meta analysis of the relationship between the neutrophils/lymphocytes ratio and prognosis in patients with oral squamous cell carcinoma. J Prev Treat Stomatol Dis. 2022;30:191–9.

Reason: Other language than english

6. Chambers DW, Thakkar D. Consistency of orthodontists’ clinical decisions: A systematic review, meta-analysis, and theory development. Am J Orthod Dentofacial Orthop. 2022;161:497-509.e4.

Reason: Intervention

7. Kazemian E, Solinski M, Adams W, Moore M, Thorpe EJ. The role of perineural invasion in parotid malignancy outcomes: A systematic review and meta-analysis. Oral Oncol. 2022;130.

Reason: Intervention

8. Minervini G, Mariani P, Fiorillo L, Cervino G, Cicciù M, Laino L. Prevalence of temporomandibular disorders in people with multiple sclerosis: A systematic review and meta-analysis. Cranio J Craniomandibular Sleep Prac. 2022. https://doi.org/10.1080/08869634.2022.2137129.

Reason: Intervention

9. Sáenz-Ravello G, Matamala L, dos Santos NC, Cisternas P, Gamonal J, Fernandez A, et al. Healthy Dietary Patterns on Clinical Periodontal Parameters: A GRADE Compliant Systematic Review and Meta-analysis. Cur Oral Heal Rep. 2022;9:32–55.

Reason: Intervention

10. Gopal SK, Priyadharshini S, Poongodi V, Harsha Vardhan BG. Diagnostic efficacy of computed tomography and magnetic resonance imaging in detection of cervical lymph node metastasis among patients with oral cancer in India - Systematic review and meta-analysis. J Head Neck Physician Surg. 2022;10:132–41.

Reason: Other language than english

11. Martins JNR, Zhang Y, von Zuben M, Vargas W, Seedat HC, Santiago F, et al. Worldwide Prevalence of a Lingual Canal in Mandibular Premolars: A Multicenter Cross-sectional Study with Meta-analysis. J Endod. 2021;47:1253–64.

Reason: Other study design

12. Mallo Magariños M, Suárez Ajuria M, Marichalar Mendía X, Álvarez-Calderón Iglesias Ó, Chamorro Petronacci CM, García García A, et al. Diagnostic yield of sentinel lymph node biopsy in oral squamous cell carcinoma T1/T2-N0: systematic review and meta-analysis. Int J Oral Maxillofac Surg. 2021;50:1271–9.

Reason: Intervention

13. Suazo J. Environmental factors in non-syndromic orofacial clefts: A review based on meta-analyses results. Oral Dis. 2022;28:3–8.

Reason: Other study design

14. Caldeira PC, Soto AML, de Aguiar MCF, Martins CC. Tumor depth of invasion and prognosis of early-stage oral squamous cell carcinoma: A meta-analysis. Oral Dis. 2020;26:1357–65.

Reason: Intervention and other study design

15. Zeng B, Yang L, Liang Y-J, Lao X-M, Mei X-Y, Liao G-Q. Diagnostic value of intraoperative bone marrow assessment for bone margins in patients with head and neck squamous cell carcinoma: a systematic review and meta-analysis. Int J Oral Maxillofac Surg. 2020;49:1128–34.

Reason: Intervention

16. Sharma VK, Shukla NK, Chaturvedi TP, Singh S. Variables to predict spontaneous eruption of palatally displaced permanent canine after interceptive extraction of primary canine: A systematic review and meta-analysis. Int Orthod. 2021;19:25–36.

Reason: Intervention

17. Angst PDM, Maier J, dos Santos Nogueira R, Manso IS, Tedesco TK. Oral health status of patients with leukemia: a systematic review with meta-analysis. Arch Oral Biol. 2020;120.

Reason: Intervention

18. Papadiochou S, Papadiochos I, Perisanidis C, Papadogeorgakis N. Medical practitioners’ educational competence about oral and oropharyngeal carcinoma: a systematic review and meta-analysis. Br J Oral Maxillofac Surg. 2020;58:3–24.

Reason: Intervention

19. Mohideen K, Krithika C, Jeddy N, Bharathi R, Thayumanavan B, Sankari SL. Meta-analysis on risk factors of squamous cell carcinoma of the tongue in young adults. J Oral Maxillofac Pathol. 2019;23:450–7.

Reason: Other study design

20. Sachdev PK, Freeland-Graves J, Beretvas SN, Sanjeevi N. Zinc, Copper, and Iron in Oral Submucous Fibrosis: A Meta-Analysis. Int J Dent. 2018;2018.

Reason: Other study design

21. Farooq S, Khandavilli S, Dretzke J, Moore D, Nankivell PC, Sharma N, et al. Transoral tongue base mucosectomy for the identification of the primary site in the work-up of cancers of unknown origin: Systematic review and meta-analysis. Oral Oncol. 2019;91:97–106.

Reason: Intervention

22. Schwendicke F, Elhennawy K, El Shahawy O, Maher R, Gimenez T, Mendes FM, et al. Visual and radiographic caries detection: A tailored meta-analysis for two different settings, Egypt and Germany. BMC Oral Health. 2018;18.

Reason: Other study design

23. Ramos-García P, González-Moles M, González-Ruiz L, Ruiz-Ávila I, Ayén Á, Gil-Montoya JA. Prognostic and clinicopathological significance of cyclin D1 expression in oral squamous cell carcinoma: A systematic review and meta-analysis. Oral Oncol. 2018;83:96–106.

Reason: Intervention

24. Rocha MR, Lins L, Cattony AC. Lichen planus and hepatitis C virus association: A meta-analysis study. Rev Port Estomatol Med Dent Cir Maxilofac. 2018;59:2–9.

Reason: Not available

25. Klein Nulent TJW, Noorlag R, Van Cann EM, Pameijer FA, Willems SM, Yesuratnam A, et al. Intraoral ultrasonography to measure tumor thickness of oral cancer: A systematic review and meta-analysis. Oral Oncol. 2018;77:29–36.

Reason: Intervention

26. Jayaprakash C, Varghese VK, Bellampalli R, Radhakrishnan R, Ray S, Kabekkodu SP, et al. Hypermethylation of Death-Associated Protein Kinase (DAPK1) and its association with oral carcinogenesis - An experimental and meta-analysis study. Arch Oral Biol. 2017;80:117–29.

Reason: Other study design

27. Kumar R, Ahmed SS, Hashmi GS, Ansari MK, Rahman SA. Meta Analysis of Etiology and its Clinical and Radiological Correlation in Cases of Craniomaxillofacial Trauma. J Maxillofac Oral Surg. 2016;15:336–44.

Reason: Other study design

28. Ju J, Li Y, Chai J, Ma C, Ni Q, Shen Z, et al. The role of perineural invasion on head and neck adenoid cystic carcinoma prognosis: a systematic review and meta-analysis. Oral Surg Oral Med Oral Pathol Oral Radiol. 2016;122:691–701.

Reason: Intervention

29. Müller A, Hussein K. Meta-analysis of teeth from European populations before and after the 18th century reveals a shift towards increased prevalence of caries and tooth loss. Arch Oral Biol. 2017;73:7–15.

Reason: Other study design

30. Tang Q, Fu H, Qin B, Hu Z, Liu Y, Liang Y, et al. A possible link between rheumatoid arthritis and periodontitis: A systematic review and meta-analysis. Int J Periodontics Restorative Dent. 2016;37:79–86.

Reason: Not available

31. Papageorgiou SN, Papadopoulos MA, Athanasiou AE. Assessing small study effects and publication bias in orthodontic meta-analyses: A meta-epidemiological study. Clin Oral Invest. 2014;18:1031–44.

Reason: Not available

32. Zou Q-H, Li R-Q. Helicobacter pylori in the oral cavity and gastric mucosa: A meta-analysis. J Oral Pathol Med. 2011;40:317–24.

Reason: Other study design

33. Ravindran V, Ravindran Nair KS. Metaanalysis of Maxillofacial Trauma in the Northern Districts of Kerala: One Year Prospective Study. J Maxillofac Oral Surg. 2011;10:321–7.

Reason: Other study design

34. Jayaprakash V, Reid M, Hatton E, Merzianu M, Rigual N, Marshall J, et al. Human papillomavirus types 16 and 18 in epithelial dysplasia of oral cavity and oropharynx: A meta-analysis, 1985-2010. Oral Oncol. 2011;47:1048–54.

Reason: Other study design

35. Papadopoulos MA, Chatzoudi M, Karagiannis V. Assessment of characteristic features and dental anomalies accompanying tooth transposition: A meta-analysis. Am J Orthod Dentofacial Orthop. 2009;136:308.e1-308.e10.

Reason: Other study design

36. Blanco R, Colombo A, Suazo J. Maternal obesity is a risk factor for orofacial clefts: A meta-analysis. Br J Oral Maxillofac Surg. 2015;53:699–704.

Reason: Other study design

37. Papadopoulos MA, Chatzoudi M, Kaklamanosc EG. Prevalence of tooth transposition : A meta-analysis. Angle Orthod. 2010;80:275–85.

Reason: Other study design

38. Hua F, He H, Ngan P, Bouzid W. Prevalence of peg-shaped maxillary permanent lateral incisors: A meta-analysis. Am J Orthod Dentofacial Orthop. 2013;144:97–109.

Reason: Other study design

39. Corrêa-Faria P, Petti S. Are overweight/obese children at risk of traumatic dental injuries? A meta-analysis of observational studies. Dent Traumatol. 2015;31:274–82.

Reason: Other study design

40. Sánchez OH, Berrocal MIL, González JMM. Metaanalysis of the epidemiology and clinical manifestations of odontomas. Med Oral Patol Oral Cir Bucal. 2008;13:E730–4.

Reason: Other study design

41. Alvira-González J, Gay-Escoda C. Non-syndromic multiple supernumerary teeth: Meta-analysis. J Oral Pathol Med. 2012;41:361–6.

Reason: Other study design

42. Corica A, Caprioglio A. Meta-analysis of the prevalence of tooth wear in primary dentition. Eur J Paediatr Dent. 2014;15:385–8.

Reason: Other study design

43. Atieh MA. Accuracy of real-time polymerase chain reaction versus anaerobic culture in detection of Aggregatibacter actinomycetemcomitans and Porphyromonas gingivalis: A meta-analysis. J Periodontol. 2008;79:1620–9.

Reason: Other study design

44. Rakhshan V, Rakhshan H. Meta-analysis of congenitally missing teeth in the permanent dentition: Prevalence, variations across ethnicities, regions and time. Int Orthod. 2015;13:261–73.

Reason: Other study design

45. Termine N, Giovannelli L, Matranga D, Caleca MP, Bellavia C, Perino A, et al. Oral human papillomavirus infection in women with cervical HPV infection: New data from an Italian cohort and a metanalysis of the literature. Oral Oncol. 2011;47:244–50.

Reason: Other study design

46. Alvarez Amézaga J, Barbier Herrero L, Pijoan Del Barrio JI, Martín Rodríguez JC, Romo Simón L, Genolla Subirats J, et al. Diagnostic efficacy of sentinel node biopsy in oral squamous cell carcinoma. Cohort study and meta-analysis. Med Oral Patol Oral Cir Bucal. 2007;12:201–9.

Reason: Other study design

47. Xu G-Z, Guan D-J, He Z-Y. 18FDG-PET/CT for detecting distant metastases and second primary cancers in patients with head and neck cancer. A meta-analysis. Oral Oncol. 2011;47:560–5.

Reason: Other study design

48. Moles DR, Downer MC, Speight PM. Meta-analysis of measures of performance reported in oral cancer and precancer screening studies. Brit Dent J. 2002;192:340–4.

Reason: Other study design

49. Mattheeuws N, Dermaut L, Martens G. Has hypodontia increased in Caucasians during the 20th century? A meta-analysis. Eur J Orthod. 2004;26:99–103.

Reason: Other study design

50. Polder BJ, Van’t Hof MA, Van Der Linden FPGM, Kuijpers-Jagtman AM. A meta-analysis of the prevalence of dental agenesis of permanent teeth. Community Dent Oral Epidemiol. 2004;32:217–26.

Reason: Other study design

51. Miles PG, Vig PS, Weyant RJ, Forrest TD, Rockette Jr. HE. Craniofacial structure and obstructive sleep apnea syndrome--a qualitative analysis and meta-analysis of the literature. Am J Orthod Dentofacial Orthop. 1996;109:163–72.

Reason: Other study design

52. De Kanter RJAM, Battistuzzi PGFCM, Kayser AF, Truin GJ, Burgersdijk RCW, Van’t Hop MA, et al. Prevalence in the Dutch Adult Population and a Meta-analysis of Signs and Symptoms of Temporomandibular Disorder. J Dent Res. 1993;72:1509–18.

Reason: Other study design

53. Pastore A, Calura G, Carinci F. Stage grouping for head and neck cancer: a meta-analysis. Minerva Stomatol. 2001;50:285–98.

Reason: Other language than english

54. Haugejorden O. Using the DMF gender difference to assess the “major” role of fluoride toothpastes in the caries decline in industrialized countries: A meta-analysis. Community Dent Oral Epidemiol. 1996;24:369–75.

Reason: Other study design

55. Van Rijkom HM, Verdonschot EH. Factors involved in validity measurements of diagnostic tests for approximal caries - a meta-analysis. Caries Res. 1995;29:364–70.

Reason: Other study design

56. Miller CS, Johnstone BM. Human papillomavirus as a risk factor for oral squamous cell carcinoma: A meta-analysis, 1982-1997. Oral Surg Oral Med Oral Pathol Oral Radiol Endod. 2001;91:622–35.

Reason: Other study design

57. da Silva A, Muniz R, Lago M, da Silva E, Braz R. Clinical Efficacy of Mouthwashes with Potassium Salts in the Treatment of Dentinal Hypersensitivity: A Systematic Review and Meta-analysis. OPERATIVE DENTISTRY. 2023;48:33–50.

Reason: Intervention

58. Deng W, Peng W, Wang T, Chen J, Zhu S. Overexpression of MMPs Functions as a Prognostic Biomarker for Oral Cancer Patients: A Systematic Review and Meta-analysis. ORAL HEALTH & PREVENTIVE DENTISTRY. 2019;17:505–14.

Reason: Not available

59. Moles D, Downer M, Speight P. Meta-analysis of measures of performance reported in oral cancer and precancer screening studies. BRITISH DENTAL JOURNAL. 2002;192:340–4.

Reason: Other study design
